# Characterization of Early Disease Status in Treatment-Naive Male Paediatric Patients with Fabry Disease Enrolled in a Randomized Clinical Trial

**DOI:** 10.1371/journal.pone.0124987

**Published:** 2015-05-08

**Authors:** Frits A. Wijburg, Bernard Bénichou, Daniel G. Bichet, Lorne A. Clarke, Gabriela Dostalova, Alejandro Fainboim, Andreas Fellgiebel, Cassiano Forcelini, Kristina An Haack, Robert J. Hopkin, Michael Mauer, Behzad Najafian, C. Ronald Scott, Suma P. Shankar, Beth L. Thurberg, Camilla Tøndel, Anna Tylki-Szymańska, Uma Ramaswami

**Affiliations:** 1 Academic Medical Center, University Hospital of Amsterdam, Amsterdam, The Netherlands; 2 Genzyme Europe, Saint-Germain-en-Laye, France; 3 Hôpital du Sacré-Cœur de Montréal and University of Montreal, Montreal, QC, Canada; 4 University of British Columbia, Child and Family Research Institute, Vancouver, BC, Canada; 5 Charles University Prague, General University Hospital Prague, Prague, Czech Republic; 6 Hospital de Niños Ricardo Gutierrez, Hospital de Día Polivalente, Ciudad Autónoma de Buenos Aires, Argentina; 7 University Medical Center Mainz, Mainz, Germany; 8 Hospital São Vicente de Paulo, Passo Fundo, RS, Brazil; 9 Sanofi, Chilly-Mazarin, France; 10 Cincinnati Children's Hospital Medical Center, Cincinnati, Ohio, United States of America; 11 Departments of Pediatrics and Medicine, University of Minnesota, Minneapolis, Minnesota, United States of America; 12 Department of Pathology, University of Washington, Seattle, Washington, United States of America; 13 University of Washington School of Medicine, Seattle, Washington, United States of America; 14 Emory University School of Medicine, Decatur, Georgia, United States of America; 15 Department of Pathology, Genzyme, Framingham, Massachusetts, United States of America; 16 Department of Pediatrics, Haukeland University Hospital, Bergen, Norway; 17 Klinika Pediatrii, Żywienia i Chorób Metabolicznych Instytut “Pomnik – Centrum Zdrowia Dziecka”, Warsaw, Poland; 18 Royal Free Hospital, London, United Kingdom; Kaohsiung Medical University Hospital, TAIWAN

## Abstract

**Trial Design:**

This analysis characterizes the degree of early organ involvement in a cohort of oligo-symptomatic untreated young patients with Fabry disease enrolled in an ongoing randomized, open-label, parallel-group, phase 3B clinical trial.

**Methods:**

Males aged 5–18 years with complete α-galactosidase A deficiency, without symptoms of major organ damage, were enrolled in a phase 3B trial evaluating two doses of agalsidase beta. Baseline disease characteristics of 31 eligible patients (median age 12 years) were studied, including cellular globotriaosylceramide (GL-3) accumulation in skin (n = 31) and kidney biopsy (n = 6; median age 15 years; range 13–17 years), renal function, and glycolipid levels (plasma, urine).

**Results:**

Plasma and urinary GL-3 levels were abnormal in 25 of 30 and 31 of 31 patients, respectively. Plasma lyso-GL-3 was elevated in all patients. GL-3 accumulation was documented in superficial skin capillary endothelial cells (23/31 patients) and deep vessel endothelial cells (23/29 patients). The mean glomerular filtration rate (GFR), measured by plasma disappearance of iohexol, was 118.1 mL/min/1.73 m^2^ (range 90.4–161.0 mL/min/1.73 m^2^) and the median urinary albumin/creatinine ratio was 10 mg/g (range 4.0–27.0 mg/g). On electron microscopy, renal biopsy revealed GL-3 accumulation in all glomerular cell types (podocytes and parietal, endothelial, and mesangial cells), as well as in peritubular capillary and non-capillary endothelial, interstitial, vascular smooth muscle, and distal tubules/collecting duct cells. Lesions indicative of early Fabry arteriopathy and segmental effacement of podocyte foot processes were found in all 6 patients.

**Conclusions:**

These data reveal that in this small cohort of children with Fabry disease, histological evidence of GL-3 accumulation, and cellular and vascular injury are present in renal tissues at very early stages of the disease, and are noted before onset of microalbuminuria and development of clinically significant renal events (e.g. reduced GFR). These data give additional support to the consideration of early initiation of enzyme replacement therapy, potentially improving long-term outcome.

**Trial Registration:**

ClinicalTrials.gov NCT00701415

## Introduction

Fabry disease is a rare, debilitating, X-linked disorder caused by a deficiency of the lysosomal hydrolase α-galactosidase A (αGAL) leading to the accumulation of globotriaosylceramide (GL-3) [1]. Accumulation of GL-3 occurs in vascular endothelial and other cell types, leading to progressive organ damage resulting in a multisystemic disease [2,3]. The kidneys, heart, and brain are the most vulnerable organs. As the disease progresses, renal, cardiovascular, and cerebrovascular complications result in morbidity and, for many, early mortality [4–6]. Disease manifestations in childhood include acroparaesthesia, angiokeratoma, hypohidrosis, tinnitus/hearing difficulties, gastrointestinal (GI) symptoms, fatigue, and headache [7–9], all of which have a negative impact on quality of life (QoL).

Impairment of renal function is a prevalent complication that develops in the majority of men and in a subset of women with untreated Fabry disease [4,10–12]. A spectrum of Fabry disease-specific kidney lesions (GL-3 accumulation in a variety of renal cell types) and secondary pathology related to progression of kidney disease (i.e. glomerular sclerosis, interstitial fibrosis) have been observed even in the early clinical stages of Fabry nephropathy [12–16]. Clinical evidence of kidney involvement (e.g. microalbuminuria, proteinuria, reduced glomerular filtration rate [GFR]) has been reported in children and adolescents [7,9,12,15–18], but detailed characterization of renal disease very early in the natural history of Fabry disease has yet to be documented. The FIELD (Fabrazyme: Intervening Early at Low Dose) trial is an ongoing randomized, open-label, parallel-group, phase 3B study evaluating the long-term efficacy and safety of enzyme replacement therapy (ERT) with two low-dose treatment regimens of agalsidase beta in treatment-naive male paediatric patients with Fabry disease without clinically significant involvement of major organs (i.e. kidney, heart, and brain). The objective of this manuscript is to systematically report the baseline characteristics of this cohort of 31 treatment-naive male paediatric Fabry patients.

## Methods

The protocol for this trial and supporting CONSORT checklist are available as supporting information; see S1 CONSORT Checklist and S1 Protocol.

### Study Design

This is an ongoing randomized, open-label, parallel-group, phase 3B study to evaluate the long-term efficacy and safety of ERT. All patients and/or their parents/legal guardians provided written informed consent before any procedures were performed or protocol-specified treatments administered. Patients were randomized to two low-dose treatment regimens of agalsidase beta (Fabrazyme; Genzyme, a Sanofi company, Cambridge, MA, USA): 0.5 mg/kg every 2 weeks; or 1 mg/kg every 4 weeks for 5 years. This ongoing phase 3B study is being conducted in accordance with Good Clinical Practice as defined by the International Conference on Harmonization guidelines and the Declaration of Helsinki, and is registered at clinicaltrials.gov under the identifier NCT00701415. The protocol was approved by the following Institutional Review Boards and/or Independent Ethics Committees: Medical Ethics Committee AMC, Amsterdam, The Netherlands; National Research Ethics Service, Cambridgeshire 4 Research Ethics Committee, Norwich, United Kingdom; Bioethics Committee, Children's Memorial Health Institute, Warsaw, Poland; University of Bergen, Bergen, Norway; Ethics Committee of the General University Hospital, Prague, Czech Republic; Emory Institution Review Board, Atlanta, GA, USA; Ethics Committee for Research and Evaluation of Health Technologies, Montreal, Canada; Cincinnati Children’s Hospital Institutional Review, Cincinnati, OH, USA; Research Ethics Committee of Children’s Hospital Ricardo Gutierrez, Buenos Aires, Argentina; Teaching and Research Committee of General Children’s Hospital Ricardo Gutierrez, Buenos Aires, Argentina; Ethics Committee for Research in Humans from the Foundation University of Passo Fundo, Passo Fundo, Brazil; Western Institutional Review Board, Olympia, WA, USA; UBC and C&W Research Ethics Board, Vancouver, Canada.

The outcomes following treatment will be reported in a separate publication, once the study has been completed.

### Participants

Males aged between 5 and 18 years with a confirmed diagnosis of Fabry disease were eligible for inclusion. A diagnosis of Fabry disease was defined as a central laboratory-documented leucocyte αGAL activity of < 4 nmol/hr/mg leucocyte protein. In the absence of the preferred leucocyte assay, patients with documented plasma αGAL activity of < 1.5 nmol/hr/mL were eligible. Patients were required to have evidence of GL-3 accumulation documented by a plasma GL-3 level > 7.0 μg/mL or a urinary GL-3 level > 0.03 mg/mmol creatinine. Patients with any of the following characteristics were excluded: micro- or macroalbuminuria (first morning void urinary albumin/creatinine ratio > 30 mg/g in ≥ 2 of 3 consecutive samples, 1 week apart); GFR, measured by plasma disappearance of iohexol (iGFR), < 90 mL/min/1.73 m^2^; evidence of stroke or a transient ischaemic attack, or magnetic resonance imaging (MRI) scan demonstrating white matter hyperintensities > 2 mm in size on T2- or fluid-attenuated inversion recovery (FLAIR)-weighted images within the white matter or ganglia; severe and recurrent peripheral pain (i.e. pain episodes occurring more than once a week, with pain that influenced daily activities regardless of medication, for ≥ 3 months); evidence of left-ventricular hypertrophy (LVH) defined as left-ventricular posterior wall thickness (LVPWT) and/or inter-ventricular septum thickness (IVST) ≥ 2 standard deviations (SDs) compared to normal based on body surface area normal ranges [19]. Patients who had received prior specific treatment for Fabry disease or who had participated in any other study of an investigational drug within 30 days of participation in the present study were also excluded.

### Assessments

The following baseline assessments of disease were made at the screening visit, before randomization to treatment.

#### Plasma and urinary GL-3

Plasma and urine samples were assayed for GL-3 using tandem mass spectrometry. The upper limit of normal (ULN) was 7.0 μg/mL for the plasma assay [20] and < 0.030 mg/mmol creatinine for the urine assay. Plasma samples were also analysed for the isoform lyso-GL-3 (ULN 3 ng/mL).

#### Accumulation of GL-3 in the skin

GL-3 accumulation was determined from skin biopsy samples. A 3-mm punch biopsy was taken under local anaesthesia, and masked biopsy specimens, stained as previously described [21], were analysed using light microscopy (LM). Biopsy specimens were processed immediately after collection, and stored at 4°C in polystyrene vials containing 15 mL of 3% glutaraldehyde in 0.2 mol/L sodium cacodylate buffer (pH 7.3) until shipped in glutaraldehyde to the Genzyme Department of Pathology (Framingham, MA, USA). The level of GL-3 accumulation in skin capillary endothelial cells and, where present, deep vessel endothelial cells, deep vessel smooth muscle cells, and perineurial cells was scored as described previously by Thurberg et al. [21]. Scoring was based on a none, mild, moderate, and severe (0, 1, 2, and 3) scale. A GL-3 score of 0 (none/trace) was considered normal, and GL-3 scores of 1 (mild), 2 (moderate), and 3 (severe) were considered abnormal [21].

#### Renal function

GFR was measured using iGFR, based on 6-point sampling including a pre-injection time point [22]. GFR was also estimated from serum creatinine levels (eGFR) using age-appropriate formulae (eGFR_Bedside Schwartz_ for patients aged < 18 years or eGFR_CKD-EPI_ for patients aged 18 years) [23,24]. Median albuminuria and proteinuria values were obtained from 3 consecutive first morning urine voids, taken ≥ 1 week apart. Urine beta-2-microglobulin and retinol binding protein were assayed as biomarkers for renal tubular damage.

#### Evaluation of GL-3 and tissue damage in the kidney

Kidney biopsies were obtained from patients on an optional basis in order to assess the extent of histological renal involvement in this treatment-naive paediatric cohort. The method used for preparation of kidney biopsy samples has been previously reported by Thurberg et al. [25]. LM was used to assess the level of focal/global glomerulosclerosis.

Quantitative glomerular structural analysis was performed on masked kidney biopsy specimens using electron microscopy (EM). Fractional volume of GL-3 inclusions per cytoplasm of podocytes (Vv[Inc/PC]), endothelial cells (Vv[Inc/Endo]), and mesangial cells (Vv[Inc/Mes]); foot process width (evaluating podocyte effacement); and percentage endothelial fenestration were estimated using systematic, unbiased morphometric sampling, and quantitative methods as detailed elsewhere [16,26,27]. In addition, GL-3 inclusions in extraglomerular cells were assessed (not quantitatively). All EM measurements (with exception of arteriopathy) were done in uncompromised glomerular structures with intact Bowman’s capsule without obvious mechanical distortion (2–3 glomeruli per biopsy).

#### Other assessments

Additional assessments included audiology evaluations, listing of GI symptoms, and assessment of angiokeratoma, if present. Analysis of *GLA* mutation was performed at screening when the nucleotide sequence of the mutation was not available. QoL was assessed using the Pediatric Quality of Life Inventory (PedsQL) Generic Core Scales, Multidimensional Fatigue Scale, and the Paediatric Pain Scale (visual analogue scale). On the pain scale, a score of 0 indicated no pain or discomfort, and a score of 100 represented severe pain.

### Settings and Locations of Data Collection

Data — including audiology assessments, eye examination, echocardiography, and brain MRI scans — were collected at the 12 study sites. The listing of the study sites is provided in the Acknowledgments section. Analyses of GL-3 (plasma and urine samples), lyso-GL-3 (plasma samples), GFR, urinary protein, albumin and creatinine levels, as well as all histological assessments (LM for GL-3 scoring of skin biopsies, LM for focal/global glomerulosclerosis, and EM for kidney biopsy evaluations) were carried out centrally. Echocardiograms, electrocardiograms (ECGs) and MRI scans were also analysed centrally. *GLA* genotyping was carried out centrally only when not already available at the site.

### Statistical Analyses

No formal statistical sample size calculation was performed. The number of patients to be enrolled was determined on medical grounds as a compromise between the need to assess safety, efficacy, and pharmacokinetics in the largest population, and the limitation for recruitment of this paediatric population in the context of this orphan disease.

Baseline demographics and other patient characteristics were summarized descriptively. Regression analysis was performed to model the linear relationship between age and pain score. A p value of 0.05 was considered statistically significant.

## Results

### Patient Demographic Characteristics and Disposition

The study was initiated in May 2008, the first patient enrolled in September 2008 and the last patient started the study in May 2010; the study is ongoing until May 2015. Of the 44 patients screened, 10 did not meet the inclusion criteria and 3 withdrew consent prior to receiving treatment (S1 Fig). The primary reasons for screening failures were residual leucocyte or plasma αGAL activity above the protocol-defined cutoffs (n = 7), plasma or urine GL-3 accumulation below the protocol-defined cutoffs (n = 4), and echocardiographic evidence of LVH based on LVPWT and/or IVST ≥ 2 SD (n = 2). The two patients with evidence of LVH were brothers, aged 16 and 18 years at the time of screening. Two patients, one with residual αGAL activity (aged 18 years) and the 16-year-old boy with LVH, also had iGFR < 90 mL/min/1.73 m^2^. Therefore, 31 patients were randomized. The baseline demographics and clinical characteristics are summarized in Table 1. The median age at enrolment was 12.0 years (range 5–18 years).

**Table 1:** Demographics and clinical characteristics of treatment-naive paediatric patients with non-severe Fabry disease.^a^

Parameter	Patients (N = 31)
Male sex, n (%)	31 (100)
Median age at Fabry diagnosis (range), years	10 (0–17)
Median age at study enrolment (range), years	12 (5–18)
Patients aged 5–11 years, n (%)	15 (48)
Patients aged 12–18 years, n (%)	16 (52)
White ethnicity, n (%)	29 (94)
Mean heart rate (SD), bpm	75 (16)
Mean systolic blood pressure (SD), mmHg	111 (14)
Mean diastolic blood pressure (SD), mmHg	61 (9)
Family member with Fabry disease, n (%)	30 (97)
αGAL activity, n (%)	
Leucocyte αGAL activity < 4.0 nmol/hr/mg^b^	24 (77)
Plasma αGAL activity < 1.5 nmol/hr/mL	31 (100)
Mean GL-3 (SD)	
Plasma, μg/mL^c^	9.9 (2.9)
Urinary, μg/mmol creatinine^d^	600 (400)
Mean plasma lyso-GL-3 (range), ng/mL^e^	142.0 (3.1–211.5)
Abnormal hearing, n (%)	
Tinnitus	4 (13)
Hearing loss	6 (19)
GI symptoms, n (%)	
Abdominal pain	20 (65)
Diarrhoea	10 (32)
Nausea	9 (29)
Vomiting	5 (16)
Angiokeratomas, n (%)	15 (48)
Concomitant medication, n (%)	
≥ 1 concomitant medication during the study	19 (61)
Pain medications	14 (45)
Medications to manage GI symptoms	4 (13)

^a^Baseline demographics and clinical characteristics reported for the overall group. Baseline demographics and clinical characteristics for each of the treatment groups will be reported once the study has been completed (in the context of the outcomes of the study).

^b^Leucocyte information not available for 7 patients.

^c^Normal plasma GL-3: ≤ 7.0 μg/mL.

^d^Normal urinary GL-3: 1–30 μg/mmol creatinine.

^e^Normal plasma lyso-GL-3: ≤ 3 ng/mL.

αGAL = α-galactosidase A; bpm = beats per minute; GI = gastrointestinal; GL-3 = globotriaosylceramide; SD = standard deviation.

### *GLA* Mutations

The *GLA* mutations for all patients are presented in Table 2 [28–46]. Genotyping found missense mutations (n = 19), nonsense mutations (n = 9), and deletions (n = 3).

**Table 2:** *GLA* genotypes.

Nucleotide change	No. of patients	Codon change	Associated phenotype, as reported in the literature
c.157_160del_AACC	1	p.Asn52FsX67^a^	Novel
c.803T>C	1	p.Leu268Ser	Classic phenotype [28]
c.679C>T	2	p.Arg227X^a^	Classic phenotype [29]
c.1025G>A	1	p.Arg342Gln	Classic phenotype [30]
c.400delT	1	p.Tyr134Met_FsX31^a^	Not specified [31]
c.644A>G	1	p.Asn215Ser^b^	Cardiac variant phenotype [32]
c.950T>C	1	p.Ile317Thr	Classic phenotype [33] and cardiac variant phenotype [34]
c.802_805delTTAG	1	p.Leu268X^a^	Novel^b^
c.71G>A	3	p.Trp24X^a^	Novel
c.800T>G	1	p.Met267Arg	Classic phenotype [35]
c.277G>A	1	p.Asp93Asn	Classic phenotype [36]
c.982G>C	1	p.Gly328Arg	Classic phenotype [37]
c.748C>T	1	p.Gln250X^a^	Classic phenotype[38]
c.1042G>C	3	p.Ala348Pro	Classic phenotype [35]
c.613C>A	1	p.Pro205Thr	Classic phenotype [39]
c.101A>G	1	p.Asn34Ser	Classic phenotype [32]
c.874G>A	4	p.Ala292Thr	Not specified [40]
c.647A>G	2	p.Tyr216Cys	Classic phenotype [41]
c.1095delT	1	p.Tyr365fsX^a^	Classic phenotype [42]
c.1117G>C	1	p.Gly373Arg	Novel
c.658C>T	1	p.Arg220X^a^	Classic phenotype [43]
c.1235_6delCT	1	p.Thr412fs^c^	Classic phenotype [44–46]

^a^Signifies truncating (nonsense) mutation.

^b^This mutation could be identical to c.803delTAGT, previously described by Shabbeer et al. [33]

^c^Signifies frameshift mutation that extends protein sequence beyond natural stop codon.

### Plasma and Urinary GL-3, and Plasma Lyso-GL-3

The plasma GL-3 level was abnormal in 25 of 30 (83%) patients. Mean (SD) plasma GL-3 was 9.9 (2.9) μg/mL with values ranging from 4.6 to 16.8 μg/mL (Table 1). The urinary GL-3 level was abnormal in all 31 patients. The mean (SD) urinary GL-3 was 600 (400) μg/mmol of creatinine with values ranging from 100 to 1,600 μg/mmol (Table 1). Plasma lyso-GL-3 levels were elevated in all 31 patients, ranging from 3.1 to 211.5 ng/mL, with a mean of 142.0 ng/mL and median of 151.4 ng/mL. The patient with the lowest level had fulfilled all inclusion criteria and had a p.Asn215Ser mutation, which has been reported to be associated with the cardiac variant of Fabry disease [32]. Of note, this patient had GI symptoms and a family history of renal disease.

### GL-3 Accumulation in Skin Vascular Endothelium

**Fig 1:**
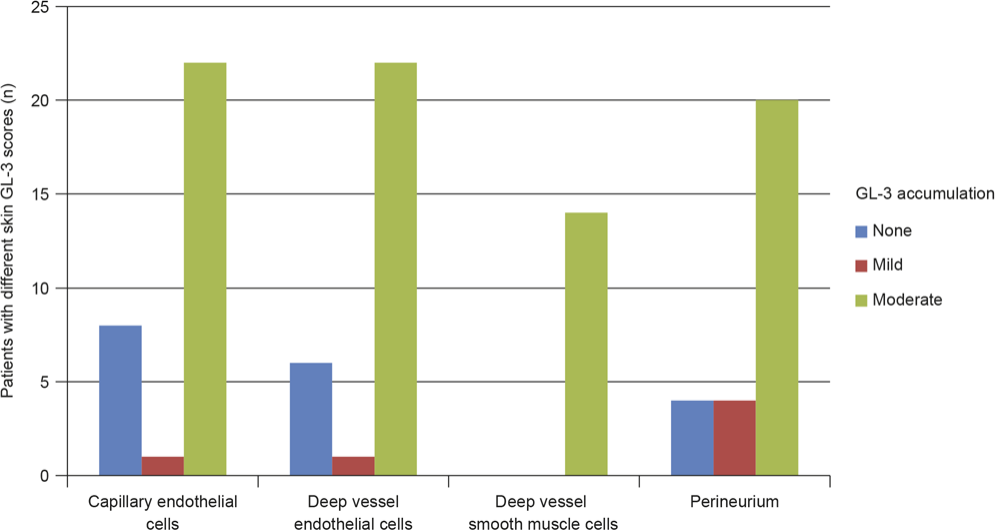
Skin GL-3 scores in treatment-naive male paediatric patients with Fabry disease. Data missing for deep vessel endothelial cells (n = 2), deep vessel smooth muscle cells (n = 17), and perineurium (n = 3); cell types not present in biopsy. GL-3 = globotriaosylceramide.

The GL-3 accumulation level in superficial skin capillary endothelial cells was scored as abnormal (> 0 scores) in 23 of 31 (74%) patients. All 23 patients with abnormal GL-3 scores had either mild or moderate accumulation (score of 1 or 2); none of the patients had evidence of severe GL-3 accumulation (Fig 1). Similarly, 23 of 29 (79%) patients who had data for GL-3 in deep vessel endothelial cells had mild or moderately abnormal GL-3 accumulations (Fig 1).

### Renal Function

**Fig 2:**
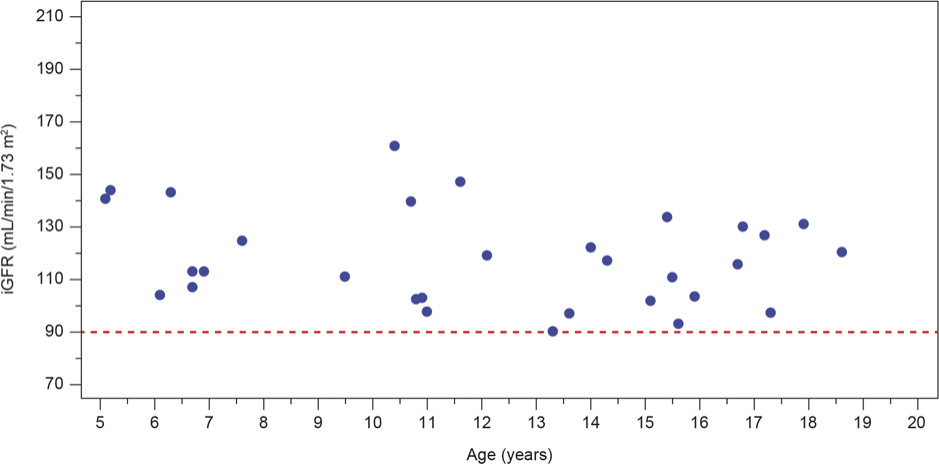
Levels of glomerular filtration rate measured by plasma disappearance of iohexol (iGFR) in treatment-naive male paediatric patients with Fabry disease. The red dashed line indicates the iGFR inclusion criterion for the study (90 mL/min/1.73 m^**2**^). iGFR = glomerular filtration rate as measured by plasma disappearance of iohexol.

**Fig 3:**
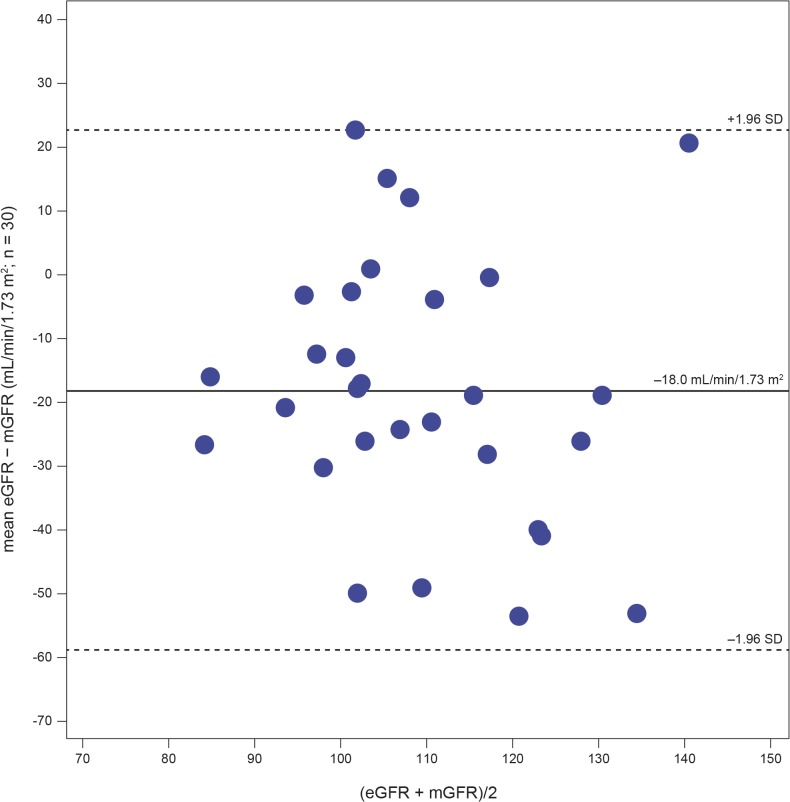
Bland–Altman plot showing the uncertainty for the eGFR Bedside Schwartz equation. GFR = glomerular filtration rate; eGFR = GFR estimated from serum creatinine levels; mGFR = measured GFR; SD = standard deviation.

The renal function findings are described in Table 3. As per inclusion criteria, the iGFR was ≥ 90 mL/min/1.73 m^2^ in all patients. The mean (SD) value for iGFR was 118.1 (18.1) mL/min/1.73 m^2^ (2 patients had high iGFR values of 147 and 161 mL/min/1.73 m^2^, respectively). iGFR values are shown in Fig 2. Age-appropriate equations did not accurately estimate GFR in this cohort of paediatric Fabry patients: the mean eGFR (SD) was 101.0 (16.6) mL/min/1.73 m^2^. The Bland–Altman plot (Fig 3) shows the uncertainty for the eGFR_Bedside Schwartz_ equation (n = 30). The bias (mean value of eGFR − measured GFR [mGFR]) was −18.0 ± 20.77 mL/min/1.73 m^2^ and the interquartile range for the difference between eGFR and mGFR values was 24.9 mL/min/1.73 m^2^. Percentages of the eGFR results within 30%, 20%, and 10% of the mGFR results (a measure of accuracy) were 86.7%, 56.7%, and 16.7%, respectively.

**Table 3:** Renal function.^a^

Parameter	Patients (N = 31)
iGFR, mL/min/1.73 m^2^	
Mean (SD)	118.1 (18.1)
Range	90.4–161.0
eGFR, mL/min/1.73 m^2^	
Mean (SD)	101.0 (16.6)
Range	71.0–151.0
Protein/creatinine ratio, mg/g creatinine^b^	
Median	92.7
Range	4.8–168.8
Albumin/creatinine ratio, mg/g creatinine^b^	
Median	10
Range	4.0–27.0

^a^Renal function data reported for the overall group. Renal function data for each of the treatment groups will be reported once the study has been completed (in the context of the outcomes of the study).

^b^To convert protein/creatinine ratio and albumin/creatinine ratio in mg/g to mg/mmol, divide by 8.84.

eGFR = GFR estimated from serum creatinine levels; GFR = glomerular filtration rate; iGFR = GFR measured by plasma disappearance of iohexol; SD = standard deviation.

As per inclusion criteria, urinary excretion of total protein and albumin was within the normal range in all patients. The median albumin/creatinine ratio was 10 mg/g. There were no abnormal results for beta-2-microglobulin, and all samples assayed were below the detectable range (< 0.3 mg/dL creatinine) for retinol-binding protein.

### Kidney Biopsy Findings

**Fig 4:**
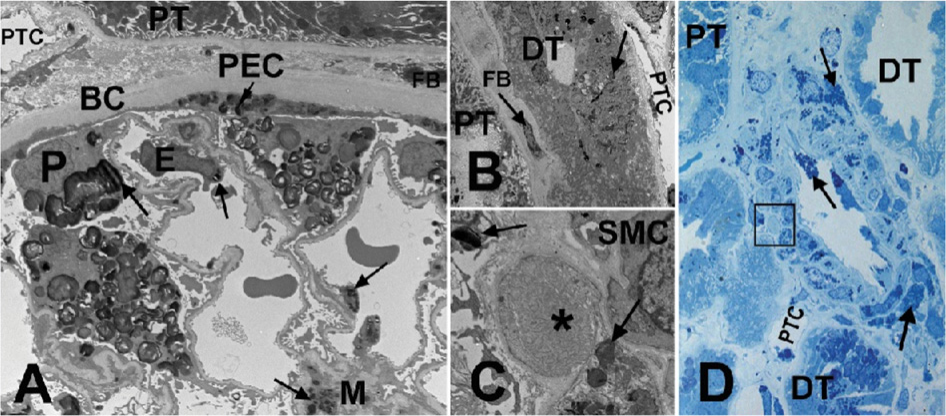
Kidney biopsy images from a male patient with Fabry disease. **(A)** EM of a glomerulus. Arrows show GL-3 inclusions in podocytes (P), endothelial cells (E), mesangial cells (M), and parietal epithelial cells (PEC). **(B)** EM of a distal tubule (DT) with GL-3 inclusions (arrows) accumulated in its epithelial cells, in contrast to the adjacent proximal tubule (PT) with no obvious GL-3 inclusion. **(C)** EM close-up of the square shown in **(D)**, displaying early Fabry arteriopathy (asterisk) focally replacing smooth muscle cells (SMC) of an arteriolar wall. Arrows show GL-3 inclusions in adjacent smooth muscle cells. **(D)** Methylene blue/Azure II (Richardson’s) stained semi-thin section of an arteriole. Note that the arteriopathy shown in **(C)** with higher magnification EM is not easily identifiable by LM (square). Arrows show GL-3 inclusions in endothelial cells and smooth muscle cells of the artery. BC = Bowman's capsule; DT = distal tubule; E = endothelial cells; EM = electron microscopy; FB = fibroblast; GL-3 = globotriaosylceramide; LM = light microscopy; M = mesangial cells; P = podocytes; PEC = parietal epithelial cells; PT = proximal tubule; PTC = peritubular capillary; SMC = smooth muscle cells. EM measurements of GL-3 deposition in glomerular cell types are shown in Table 4 [16]. Unbiased morphometric EM estimates of GL-3 in the three glomerular cell types including Vv (Inc/PC), Vv(Inc/Endo), and Vv(Inc/Mes) [16,27], as well as podocyte foot process width and percentage glomerular capillary endothelial fenestration [16,26,27] are tabulated in Table 4.

Optional kidney biopsies were performed in 6 patients aged between 13 and 17 years. No focal/global glomerulosclerosis was observed in any of the 6 kidney biopsies. Fig 4 is an example of the kidney biopsy images. One of the biopsies showed a glomerulus with cystic appearance and dilated Bowman's capsule, with a few capillary loops with adhesions to the Bowman's capsule, raising suspicion of being atubular. This glomerulus was excluded from the EM quantitative studies.

**Table 4:** Kidney biopsy findings by electron microscopy.

Case	Age (years)	Vv(Inc/PC)	Vv(Inc/Endo)	Vv(Inc/Mes)	%EF	FPW (nm)	Arteriopathy (yes/no)
1	13	0.25	0.13	0.04	44	558	Yes
2	17	0.30	0.09	0.06	61	593	Yes
3	15	0.31	0.17	0.03	56	615	Yes
4	15	0.29	0.12	0.05	52	626	Yes
5	15	0.27	0.12	0.05	57	744	Yes
6	17	0.31	0.15	0.06	56	671	Yes
Mean ± SD	15 ± 1.5	0.29 ± 0.02	0.13 ± 0.03	0.05 ± 0.01	54 ± 6	635 ± 65	Yes

Normal reference values in controls: FPW, 430 ± 61 nm; % EF, 61 ± 9 [16].

%EF = percentage glomerular capillary endothelial fenestration; FPW = foot process width; GL-3 = globotriaosylceramide; SD = standard deviation; Vv(Inc/Endo) = fractional volume of endothelial cells occupied by GL-3 inclusions; Vv(Inc/Mes) = fractional volume of mesangial cells occupied by GL-3 inclusions; Vv(Inc/PC) = fractional volume of podocytes occupied by GL-3 inclusions.

The presence of GL-3 inclusions with characteristic electron-dense lamellar appearance of myelin figures and zebra bodies was confirmed in these various cell types (Fig 4A and Fig 4B). GL-3 accumulation was found by EM in glomerular endothelial cells, mesangial cells, podocytes, and parietal epithelial cells in all 6 patients. All podocytes showed prominent accumulation of GL-3 inclusions, distinctly more abundant than in any other kidney cells. The size of the inclusions was variable, but in general, podocyte inclusions appeared to be larger than those of other cells. There was segmental effacement of foot processes over the glomerular capillary loops in all patients. In addition, GL-3 inclusions were observed in peritubular capillary endothelium, interstitial cells, non-capillary endothelial cells, vascular smooth muscle cells, and distal tubules/collecting duct cells (not quantitated).

EM revealed arteriopathy in all 6 biopsies (Fig 4C and Fig 4D), whereas LM showed mild Fabry arteriopathy only in 2 of 6 biopsies. The arteriopathy was characterized by focal replacement of arterial/arteriolar smooth muscle cells by waxy, hyaline-like material. One glomerulus showed segmental mesangiolysis and microaneurysm formation on EM, but no other features suggestive of a thrombotic microangiopathic process or any additional glomerulopathy other than Fabry nephropathy.

### Brain Morphology

Optional brain MRI scans were carried out on 21 of 31 patients: 19 were aged > 10 years and 2 were aged 6 years but did not require the use of an inhaled general anaesthetic. One patient was found to have a white matter lesion (volume 178 mm^3^) on T2/FLAIR MRI. As the presence of a white matter lesion > 2 mm was an exclusion criterion, this patient was wrongly included. However, following an *ad hoc* review conducted by the Data Monitoring Committee *a posteriori* (once the patient had been in the study for a substantial period), it was accepted that the patient could continue the study.

### Cardiac Abnormalities

As per inclusion criteria, cardiac morphology and function, assessed by echocardiography and ECG, were unremarkable. All patients had a LV ejection fraction > 60%, relative wall thickness < 0.45, and LV mass index < 50 g/m^2.7^.

### Hearing Abnormalities

Audiology examinations and audiometry (hearing) tests were normal for 25 of 31 (81%) patients. Four patients had tinnitus and six patients had a degree of hearing loss (Table 1) at the median age of 11.8 years (range 5–17 years). The type of hearing loss was conductive (n = 2), sensorineural (n = 2), or of mixed type (n = 2). Three of these six patients had hearing loss that was considered clinically significant. One patient had moderately severe unilateral sensorineural hearing loss, one had mild bilateral conductive hearing loss, and the third patient had moderate unilateral conductive hearing loss.

### Gastrointestinal Symptoms

The most frequent GI symptoms reported in this paediatric cohort were abdominal pain (n = 20; median age 13 years; range 5–18 years), diarrhoea (n = 10; median age 13.5 years; range 5–18 years), and nausea (n = 9; median age 13 years; range 6–17 years) (Table 1). These were mostly mild to moderate in severity, although 4 (13%) patients experienced severe abdominal pain. One of these patients had been the subject of a separate, independent report, due to the unusually severe GI manifestations [47].

### Angiokeratoma

Angiokeratomas were present in 15 patients. These were most commonly located on the posterior trunk (n = 6), umbilicus (n = 6), and genitalia (n = 5). The median age of patients with angiokeratoma was 14 years (range 10–17 years).

### Quality of Life

QoL was diminished and fatigue commonly reported in this paediatric patient cohort, as demonstrated by the average score of 68.8 out of 100 on the PedsQL Generic Core Scales and the PedsQL Multidimensional Fatigue Scale questionnaires. Using a visual analogue scale, the mean (SD) pain score was 13.9 (20.9) out of 100 in response to the question ‘How do you feel now?’, and 39.4 (36.0) out of 100 in response to the question ‘What was your worst pain this week?’.

For the question ‘How do you feel now?’, there was no linear relationship between age and pain score (p = 0.89), but for the question ‘What was your worst pain this week?’ the relationship reached statistical significance (p < 0.0001).

## Discussion

This paper reports early disease status in a cohort of 31 treatment-naive young males with Fabry disease without severe symptoms. Because they were enrolled in a clinical trial, the patients described in this analysis had extensive clinical and biochemical data available at baseline. This provided a unique opportunity to comprehensively characterize the early manifestations of disease in male children and adolescent patients prior to the development of clinically apparent renal disease. Currently, such information is scarce in the literature, but is of great importance for the community responsible for caring and treating patients with Fabry disease. An important clinical implication that can be drawn from our study is that, although accurate measurement of GFR and assessment of proteinuria/albuminuria are useful, cellular and tissue damage in the kidneys, in addition to extensive renal GL-3 accumulation, can be present prior to clinically apparent renal dysfunction.

As expected, mean GL-3 levels in plasma and urine were elevated, and all patients had elevated plasma lyso-GL-3 levels. The combined observations of complete enzyme deficiency, mutations described in the literature as associated with classical presentation (except for the patient with the p.Asn215Ser mutation)[28,35,41], and elevated plasma lyso-GL-3 suggest that these patients are likely to develop severe complications of Fabry disease in adulthood if not treated [48,49]. These data suggest that lyso-GL-3 has high diagnostic sensitivity in male paediatric patients with a classical presentation of Fabry disease.

This is the largest cohort of children with Fabry disease in which GFR was measured in a systematic, prospective, centralized manner [22]. As per the inclusion criteria, all patients in this study had normal values for iGFR (≥ 90 mL/min/1.73 m^2^). Age-appropriate equations were used to calculate eGFR. However, mGFR, considered the gold standard, was not predicted accurately by these equations. This corresponds with earlier findings showing wide variations from mGFR in the normal range in paediatric patients [18].

The data presented here might be indicative of disease progression at a relatively early age. Kidney biopsies, in all 6 young patients who underwent the optional biopsy, in addition to GL-3 accumulation in all cell types, showed evidence of injury to podocytes (i.e. foot process widening) and Fabry arteriopathy, despite the fact that in all patients the GFR and albuminuria were within the normal range and no severe Fabry symptoms were reported. However, it is noteworthy that despite being within the clinically accepted normal range, the median albumin/creatinine ratio (10 mg/g) was higher compared with reference values reported in healthy adolescents [50,51]. This emphasizes the importance of follow-up and monitoring of albumin/creatinine ratio at least annually in children with Fabry disease.

Similar to our findings, previous studies employing renal biopsies of children and adolescents with Fabry disease also revealed significant GL-3 accumulation. This was characterized by lamellar electron-dense material (myelin figures and zebra bodies) in various kidney cell types, including podocytes, endothelial cells, mesangial cells, parietal cells, distal tubular cells, interstitial, and vascular smooth muscle cells, in all untreated patients despite minimal or no albuminuria, and normal GFR [12,15]. A recent biopsy study of renal pathology in a cohort of 14 paediatric Fabry patients (median age 12 years) also revealed lamellar GL-3 inclusions in all the glomerular cell types of patients, including a 4-year-old boy [16]. GL-3 accumulation was most prominent in podocytes, and for the first time, age-dependent progressive accumulation of GL-3 within podocytes was demonstrated [16]. The narrow age range in the patients who had kidney biopsies in the current study resulted in only subtle variations in podocyte GL-3 density measurements.

Our findings of the EM quantitative analysis were similar to those already reported for male Fabry patients of the same age (data not shown) [27], indicating that the selection criteria for the FIELD study did not appear to select for a cohort with milder renal involvement. Similar to previously reported observations [12,15,16,52], segmental foot process effacement — a manifestation of podocyte injury — was observed in all biopsies. When compared with normal control biopsies from living kidney donors [16], the average foot process width in the 6 patients in whom biopsies were performed was significantly greater than in controls. This confirms the previous observation that podocyte injury is occurring in young patients even before clinically significant albuminuria or proteinuria manifests. Another important finding was the documentation of evidence of Fabry arteriopathy, indicating that Fabry-specific vascular lesions occur relatively early in the course of the disease. Fabry arteriopathy has been reported in 4 of 9 young patients aged ≤ 18 years by Tøndel et al. [15]. In the current study, LM detected Fabry arteriopathy in only 2 of 6 biopsies. EM examination, however, confirmed these lesions in the 2 patients, and revealed arteriopathy in another 4 patients, highlighting the importance of EM examination in detection of early vascular lesions. We also observed 2 previously undescribed glomerular lesions in 2 different biopsies, i.e. prominent segmental mesangiolysis without any other evidence of thrombotic microangiopathy, and a glomerulus with cystic appearance which raised suspicion of being atubular. Whether these lesions are of clinical significance or are related to Fabry disease is not known.

In this cohort of paediatric patients without severe symptoms, significant GL-3 accumulation was already present within multiple cell types in skin biopsies. The levels of GL-3 accumulation in skin capillary endothelial cells in this cohort are comparable with pre-treatment levels reported in adult patients [21,53,54]. A high proportion of these adults were shown to have moderate (grade 2) deposition of GL-3. In the only other study that has reported pre-treatment GL-3 levels in the skin capillary cells of paediatric patients, moderate or severe GL-3 deposits were reported in superficial dermal endothelial cells of 12 of 14 boys, including the 3 youngest patients, who were aged < 10 years when they started treatment with agalsidase beta [17]. Interestingly, the 4 patients with the lowest plasma lyso-GL-3 levels in the current cohort all had zero scores for GL-3 accumulation in superficial skin capillary endothelial cells (data not shown).

In the present cohort, pain levels, angiokeratoma, GI symptoms, brain morphology, cardiac morphology and function, and hearing status were similar to those reported in previous studies of untreated young patients [7,9,15,17,55]. However, it should be noted that the study excluded patients with overt or known cardiac or central nervous system Fabry pathology. A recent study of agalsidase beta in 10 children and adolescents with Fabry disease reported no clinical Fabry manifestations in the heart, kidney, or brain before treatment was initiated, citing pain as the major symptom in childhood Fabry disease. No skin or renal biopsies were carried out in that study [55]. An open-label study of agalsidase beta in a paediatric cohort [17] also found no clinically significant cardiac symptoms relating to Fabry disease before treatment was initiated, although GI symptoms (postprandial pain, nausea, or vomiting) were present in the majority of patients. In 2003, a survey of 35 paediatric patients reported the clinical characteristics of the disease in childhood. The principal symptoms identified included acroparaesthesia, GI symptoms, headache, tinnitus, and fatigue. Renal involvement (proteinuria) was reported in 13% of male patients [7]. Cardiac complications of Fabry disease may become apparent in childhood as subtle changes with slow but detectable progression over time [56].

In terms of QoL, the treatment-naive male children and adolescents with Fabry disease without severe symptoms described in our analysis had similar scores on the multidimensional fatigue scale and the generic core scales, as previously reported for children with underlying chronic disorders such as juvenile rheumatoid arthritis, cancer, and asthma [57].

The optimal time to initiate ERT remains controversial, although there is consensus that treatment should be initiated before irreversible organ damage has occurred. Two ERT preparations are commercially available for treatment of patients with Fabry disease: agalsidase beta (Fabrazyme) and agalsidase alfa (Replagal; Shire Human Genetic Therapies, Cambridge, MA, USA; not available in the United States). Studies in adult patients have shown that agalsidase beta treatment clears GL-3 accumulation in renal capillary endothelium. Continued reduction in podocyte GL-3 has also been observed in some adult patients with sustained agalsidase beta treatment [58], and ERT may slow the progressive decline in renal function [58–60]. Agalsidase beta at 1.0 mg/kg has been shown to slow progression to the composite clinical outcome of renal, cardiac, and cerebrovascular complications and death compared with placebo in adult patients with advanced disease [61].

In children and adolescents with Fabry disease, effective reduction or clearing of GL-3 accumulation in skin [17] and renal tissue [12] was observed, and improvements in pain, GI symptoms, and energy levels have been reported [17,55]. Importantly, a recent study in young patients (median age 16.5 years) showed that 5 years of ERT with either agalsidase alfa or agalsidase beta completely cleared GL-3 from mesangial and glomerular endothelial cells [12]. Clearance of GL-3 from podocytes was found to be dependent on the cumulative agalsidase dose, and pronounced podocyte GL-3 clearance was associated with an improvement of microalbuminuria and podocyte foot-process effacement. GFR was normal and stable throughout that study [12].

Our study has limitations. First, tissue samples from the kidney biopsies had low numbers of glomeruli (LM: 2–8 per sample; EM: 2–3 per sample); the number of glomeruli assessed by LM was lower than the number of glomeruli scored in studies using LM and the scoring system of the International Study Group of Fabry Nephropathy [12,14]. The lower number of glomeruli potentially limits the strength of the observations. However, in our study, optional kidney biopsies were conducted for 6 of the 31 enrolled patients, providing a good sample size in this paediatric population. Secondly, no age-matched controls were included in this study, preventing direct comparison of the findings in this paediatric cohort with renal structure and function in children without Fabry disease.

In conclusion, these observations in untreated male children and adolescents with Fabry disease show that significant signs of GL-3 accumulation, as well as cellular and vascular injury, are present in kidney biopsies in young patients without severe Fabry symptoms. For example, GL-3 accumulation was evident in multiple types of kidney cells, and podocyte foot process widening (indicative of podocyte injury) and Fabry arteriopathy were present in all biopsies despite normal kidney function and the absence of microalbuminuria/proteinuria. These observations add to the limited number of studies that have described the clinical manifestations and renal histopathology of Fabry disease in childhood and adolescence. Our findings highlight the need for careful and thorough clinical evaluation of all patients and for regular follow-up to monitor for early clinically significant organ involvement, such as early renal events. Kidney biopsy is useful in children diagnosed with Fabry disease to assess the level of organ involvement and to assist with treatment decisions.

## Supporting Information

S1 Checklist CONSORT checklist. (DOC)

S1 Fig CONSORT participant flow diagram. (TIF)

S1 Protocol Study NCT00701415 protocol. (PDF)
