# Supplementary figures and images for: Characterization of Early Disease Status in Treatment-Naive Male Paediatric Patients with Fabry Disease Enrolled in a Randomized Clinical Trial
Source: PLoS One. 2015 May 8;10(5):e0124987. doi: 10.1371/journal.pone.0124987 (PMC4425695; doi:10.1371/journal.pone.0124987)

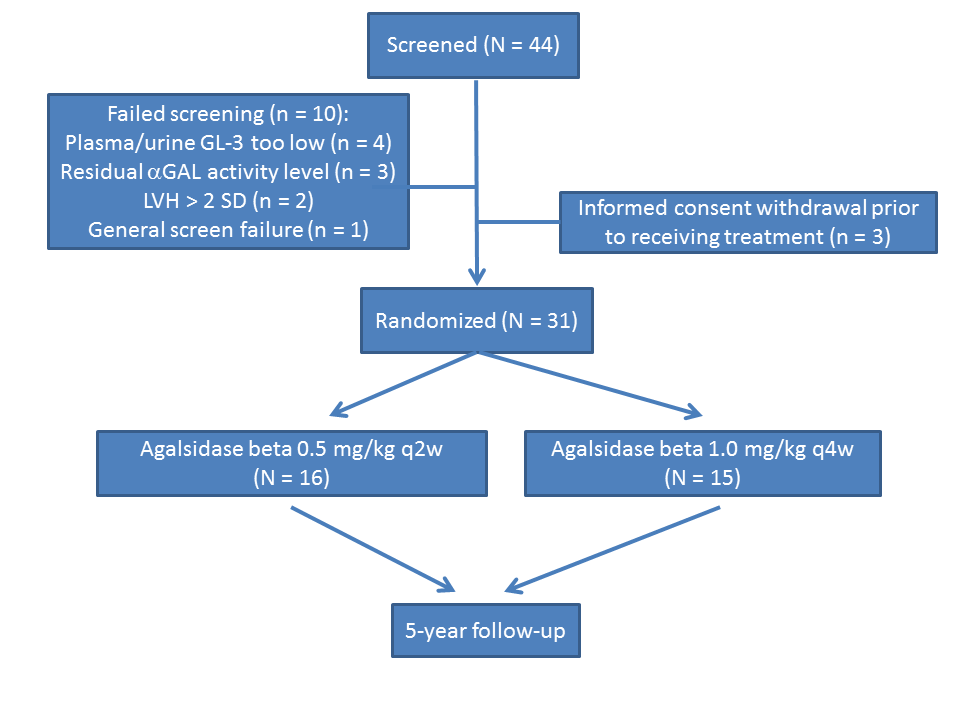

Supplement: S1 Fig — (TIF) [file pone.0124987.s002.tif]
