# Supplementary material for: Characterization of Early Disease Status in Treatment-Naive Male Paediatric Patients with Fabry Disease Enrolled in a Randomized Clinical Trial
Source: PLoS One. 2015 May 8;10(5):e0124987. doi: 10.1371/journal.pone.0124987 (PMC4425695; doi:10.1371/journal.pone.0124987)
Supplement: S1 Protocol — (PDF) [file pone.0124987.s003.pdf]

STUDY PROTOCOL

A Randomized, Multicenter, Multinational, Phase 3B, Open-Label, Parallel-Group Study of Fabrazyme® (agalsidase beta) in Treatment-Naive Male Pediatric Patients with Fabry Disease Without Severe Symptoms

Protocol Number: AGAL06207
EudraCT: 2007-005668-28

Final: 23 January 2008
Amendment 1: 04 December 2008
Amendment 2: 30 April 2010
Amendment 3: 25 June 2010

Global Study Director:	Lysa Gregoire, MS<br>Director, Clinical Research<br>Genzyme Corporation<br>+1 (617) 768-6560
Medical Monitor:	Bernard Bénichou, MD, PhD<br>Senior Medical Director, Clinical Research<br>Genzyme Europe<br>+33(0) 1 30 87 34 82
Americas Study Manager	Mollie Watkins<br>Manager, Clinical Research<br>Genzyme Corporation<br>+1 (617) 768-4497
European Study Manager:	Marta Mazur, MSc<br>Clinical Project Manager<br>Genzyme Europe, BV<br>+31(0) 35 699 8850
Statistician:	Bobbi Lemay, MPH<br>Senior Biostatistician, Biomedical Data Sciences and Informatics<br>Genzyme Corporation<br>+1 (617) 768-9141

This protocol was designed and will be conducted, recorded, and reported in compliance with the principles of Good Clinical Practice (GCP) guidelines. These guidelines are stated in United States (US) federal regulations as well as "Guidance for Good Clinical Practice," International Conference on Harmonization (ICH) of Technical Requirements for Registration of Pharmaceuticals for Human Use.

I have read and agree to abide by the requirements of this protocol.

Study Investigator Name (printed) ______________________________

Study Investigator Signature ________________________ Date ______

Signature Page for Sponsor's Representative

The following sponsor's representative has reviewed and approved the protocol entitled "A Randomized, Multicenter, Multinational, Phase 3B, Open-Label, Parallel-Group Study of Fabrazyme® (agalsidase beta) in Treatment-Naive Male Pediatric Patients with Fabry Disease Without Severe Symptoms" Amendment 3.

Bernard Bénichou, MD, PhD<br>Senior Medical Director, Clinical Research<br>Genzyme Europe	June 29th 2010<br>Date

1. SYNOPSIS

NAME OF COMPANY<br>Genzyme Corporation<br>500 Kendall Street<br>Cambridge, MA 02142<br><br>**NAME OF FINISHED PRODUCT**<br>Fabrazyme®<br><br>**NAME OF ACTIVE INGREDIENT**<br>Recombinant Human α-galactosidase A	**SUMMARY TABLE**<br>Referring to Part ……<br>of the Dossier:<br><br>Volume:<br><br>Page:<br><br>Reference:	**FOR NATIONAL AUTHORITY USE ONLY:**

TITLE: A Randomized, Multicenter, Multinational, Phase 3B, Open-Label, Parallel-Group Study of Fabrazyme (agalsidase beta) in Treatment-Naive Male Pediatric Patients with Fabry Disease Without Severe Symptoms

PROTOCOL NO.: AGAL06207

INVESTIGATOR STUDY CENTERS: Approximately 15 multinational sites will participate.

OBJECTIVES: The objectives of this open-label study are to evaluate the efficacy (GL-3 clearance), pharmacokinetics (PK), and safety parameters (including immunogenicity) for 2 alternative dose regimens of Fabrazyme (0.5 mg/kg every 2 weeks [q2w] and 1.0 mg/kg every 4 weeks [q4w]) in treatment-naive male pediatric patients (≥5 years to ≤18 years of age) with Fabry disease without severe symptoms.

METHODOLOGY: This will be a randomized, multicenter, multinational, open-label, parallel-group study to evaluate 2 alternative dose regimens of Fabrazyme (0.5 mg/kg q2w and 1.0 mg/kg q4w) in treatment-naive male pediatric patients with Fabry disease without severe symptoms. Approximately 35 male patients ≥5 and ≤18 years of age will receive treatment with Fabrazyme. Patients will be stratified by age at enrollment (5 to 11 years [children] and 12 to 18 years [adolescents]), to allow for a similar age representation in the 2 treatment arms, then randomized to receive intravenous (IV) infusions of Fabrazyme at a dose of 0.5 mg/kg q2w or 1.0 mg/kg q4w. Patients will be treated for 5 years (260 weeks). Patients who meet specific criteria may have the option of receiving infusions at home after Week 28/Month 6/Year 0.5 for the q2w infusion patients and Week 52/Year 1 for the q4w infusion patients. An independent Data Monitoring Committee (DMC) will oversee safety and dose increase due to disease progression. In cases of documented and significant progression of Fabry disease, the dose of Fabrazyme may be increased to the approved dosing regimen of 1.0 mg/kg q2w in the patients concerned, after consultation with the DMC and agreement of the Sponsor. Patients switched to 1.0 mg/kg q2w will continue to be evaluated in the study. After participating in the study, patients are encouraged to enter the Fabry Registry.

NUMBER OF SUBJECTS: Approximately 35 treatment-naive male pediatric patients will be enrolled.

DIAGNOSIS/INCLUSION CRITERIA: Patients who meet the following inclusion criteria will be eligible for enrollment in this study:

1. The patient and/or patient's parent(s)/legal guardian(s) must provide written informed assent/consent prior to any protocol-related procedures being performed.
2. The patient must have a confirmed diagnosis of Fabry disease as documented by leukocyte α-Galactosidase A (αGAL) activity of <4 nmol/hr/mg leukocyte (preferred assay; results from a central laboratory). If the leukocyte αGAL activity assay is difficult to obtain, the patient may be enrolled based on documented plasma αGAL <1.5 nmol/hr/mL, with the agreement of the Medical Monitor (results from a central laboratory).
3. The patient must have evidence of globotriaosylceramide (GL-3) accumulation as documented by plasma GL-3 (>7.0 µg/mL) or urinary GL-3 (>0.03 mg GL-3/mmol creatinine) levels (results from a central laboratory).
4. The patient must be male ≥5 and ≤18 years of age.

NAME OF COMPANY<br>Genzyme Corporation<br>500 Kendall Street<br>Cambridge, MA 02142<br><br>NAME OF FINISHED PRODUCT<br>Fabrazyme®<br><br>NAME OF ACTIVE INGREDIENT<br>Recombinant Human α-galactosidase A	SUMMARY TABLE<br>Referring to Part ……<br>of the Dossier:<br><br>Volume:<br><br>Page:<br><br>Reference:	FOR NATIONAL AUTHORITY USE ONLY:

EXCLUSION CRITERIA: Patients who meet any of the following exclusion criteria will not be eligible for enrollment in this study:

1. Patient has albuminuria (first morning void urinary albumin/creatinine ratio >30 mg/g on at least 2 out of 3 consecutive samples, each at least 1 week apart).
2. Patient has a $GFR_{iohexol}$ <90 mL/min/1.73 m^2. In case of properly documented low protein intake, values as low as 80 mL/min/1.73 m^2 may be acceptable, after consultation with the Medical Monitor.
3. Patient has documented evidence of stroke or transient ischemic attack (TIA) or, if brain magnetic resonance imaging (MRI) has been performed, bright lesions >2 mm on T2- or fluid attenuated inversion recovery (FLAIR)-weighted images within the white matter or the basal ganglia.
4. Patient has severe and recurrent acroparesthesia, judged by the physician as frequent (more than once a week) pain episodes for at least 3 months that influence daily activities, irrespective of medication.
5. Patient has an end-diastolic left ventricular posterior wall thickness (LVPWTd) and/or an end-diastolic interventricular septum thickness (IVSTd) ≥2 standard deviations (SD) compared to normal (based on body surface area [BSA] normal ranges from Kampmann, et al 2000) as read at the study site.
6. Patient has received prior treatment specific to Fabry Disease.
7. Patient has participated in a study employing an investigational drug within 30 days of the start of their participation in this study.
8. Patient has any medical condition or extenuating circumstance, which, in the opinion of the Study Investigator, could interfere with study compliance.
9. Patient has any medical condition or extenuating circumstance, e.g., diabetes mellitus, which, in the opinion of the Study Investigator, could interfere with the interpretation of study results.
10. Patient is on treatment with angiotensin converting enzyme inhibitors/angiotensin receptor blockers (ACEIs/ARBs).
11. Patient has any contra-indication mentioned in the labeling of Fabrazyme and/or iohexol (Omnipaque).
12. Patient or parent(s)/legal guardian(s) is unwilling to comply with the requirements of the protocol.

DOSE/ROUTE/REGIMEN: Patients will be treated with Fabrazyme 0.5 mg/kg q2w or 1.0 mg/kg q4w (up to 131 or 66 infusions, respectively). The infusions will be administered at an initial rate of no more than 15 mg/hr. After 8 infusions and after patient tolerance has been established, the infusion rate can be increased by 5 mg/hr at each subsequent visit. For patients who are randomized to receive 0.5 mg/kg q2w, the total infusion time should not be less than 45 minutes and for patients who are randomized to receive 1.0 mg/kg q4w the total infusion time should not be less than 90 minutes. The infusions can either be infused at a constant rate or titrated. Patients are required to be observed in the study site or at home for approximately 1 hour after each infusion. In cases of documented and significant progression of Fabry disease, the dose of Fabrazyme may be increased to the approved dosing regimen of 1.0 mg/kg q2w in the patients concerned, after consultation with the DMC and agreement of the Sponsor. These patients will continue to be evaluated in the study.

STUDY DURATION: Each patient will be treated for 5 years (260 weeks).

REFERENCE TREATMENT: Not applicable.

CRITERIA FOR EVALUATION:

Efficacy Endpoints:

Primary Efficacy Endpoint: The primary efficacy endpoint will be histological evaluation of GL-3 inclusions in the superficial skin vascular endothelium conducted using light microscopy (LM) after randomization and prior to the first infusion on Day 1, Week 52/Year 1, Week 156/Year 3, and Week 260/Year 5 or at early withdrawal.

NAME OF COMPANY<br>Genzyme Corporation<br>500 Kendall Street<br>Cambridge, MA 02142<br><br>NAME OF FINISHED PRODUCT<br>Fabrazyme®<br><br>NAME OF ACTIVE INGREDIENT<br>Recombinant Human α-galactosidase A	SUMMARY TABLE<br>Referring to Part ……<br>of the Dossier:<br><br>Volume:<br><br>Page:<br><br>Reference:	FOR NATIONAL AUTHORITY USE ONLY:

Secondary Efficacy Endpoints: The secondary efficacy endpoints will be the effect of Fabrazyme treatment on GL-3 clearance in plasma and urine collection measured at Screening and every 3 months for the first year (through Week 52/Year 1) and every 6 months thereafter or at early withdrawal.

Exploratory Efficacy Endpoints:

1. Renal Function: GFR will be calculated at Screening and once a year by iohexol plasma clearance. Estimated GFR will also be calculated at Screening and every 6 months using an age appropriate method. Three first morning void urine samples will be obtained (each at least 1 week apart) at Screening and every 6 months for assessment of the total protein/creatinine and albumin/creatinine ratios, and retinol binding protein (RBP) and β_2-microglobulin levels. All samples will be sent to a central laboratory.
2. Audiology: Standard hearing examinations and audiograms (assessed by a head, ears, eyes, nose, and throat [HEENT] specialist or equivalent) will be performed at Screening and once a year or at early withdrawal.
3. Body Mass Index (BMI): BMI will be calculated from weight and height values collected at Screening, every 3 months through Week 52/Year 1, and every 6 months thereafter or at early withdrawal.
4. Magnetic Resonance Imaging (MRI): An optional MRI (without contrast agent) of the brain may be performed at Screening, Week 132/Year 2.5 and Week 260/Year 5 or at early withdrawal and sent to a central laboratory.
5. Echocardiography: Echocardiography parameters will be measured at Screening and once a year or at early withdrawal (read at a central laboratory) and will include an evaluation of standard cardiac dimensions, calculated ejection fraction (EF), and valve abnormalities. Tissue doppler imaging (TDI) parameters (optional; based on the site's technical capabilities) will also be collected to evaluate mitral annulus velocities.
6. Retinal Imaging: Optional retinal digital imaging photographs (from a retinal photo of each eye) may be taken at Screening, Week 132/Year 2.5 and Week 260/Year 5 or at early withdrawal.
7. Angiokeratoma: Angiokeratoma and any changes thereof will be fully described at each physical examination. Digital photograph(s) of angiokeratoma will be taken at Screening, Week 132/Year 2.5, and Week 260/Year 5 and at any new occurrence/change at any time.
8. Gastrointestinal (GI) Symptoms: GI symptoms will be evaluated at Day 1, every 3 months through Week 52/Year 1 and every 6 months thereafter or at early withdrawal.
9. Quality of Life: Age-appropriate self-report instruments designed to measure the core dimensions of health and role functioning (PedsQL™ Pediatric or Young Adult Quality of Life Inventory), fatigue (PedsQL™ Multidimensional Fatigue Scale™) and pain (Pediatric Pain Questionnaire™ [PPQ™] or Brief Pain Inventory (Short Form) (BPI [SF]) if 18 years or older) will be administered at Day 1 and every 6 months thereafter or at early withdrawal.
10. Kidney biopsy: An optional kidney biopsy may be conducted after randomization but prior to Day 1 and at Week 260/Year 5 for histological evaluation or at early withdrawal. An additional kidney biopsy will be recommended prior to dose increase, if justified, based on renal findings as defined below.
11. Biomarkers: Blood and urine samples for assessing exploratory biomarkers will be collected at Screening, Day 1, and every 3 months through Week 52/Year 1 and every 6 months thereafter or at early withdrawal and sent to a central laboratory. No genetic testing will be performed on these samples.
12. Comparison: Natural history patient cohorts may be used as comparator groups, as appropriate.

<table>
<tr><td>NAME OF COMPANY
Genzyme Corporation
500 Kendall Street
Cambridge, MA 02142

NAME OF FINISHED PRODUCT
Fabrazyme®

NAME OF ACTIVE INGREDIENT
Recombinant Human α-galactosidase A</td><td>SUMMARY TABLE
Referring to Part ……
of the Dossier:

Volume:

Page:

Reference:</td><td>FOR NATIONAL AUTHORITY USE ONLY:</td></tr>
<tr><td colspan="3">Criteria for Increasing the Study Drug Dose: Any of the following may warrant a Fabrazyme dose increase to 1 mg/kg q2w, after consultation with the DMC and agreement of the Sponsor:
• Insufficient clearance (a score of 2 or more) of GL-3 from skin vascular endothelium (superficial) after Screening.
• Stroke or TIA or, if a brain MRI has been performed, the development of bright lesions >2 mm on T2- or FLAIR-weighted images within the white matter or the basal ganglia (from the central laboratory).
• Albuminuria (first morning void urinary albumin/creatinine ratio >30 mg/g on at least 2 out of 3 consecutive samples, each at least 1 week apart) (from the central laboratory).
• Echocardiography findings (from central reading center):
  o Patients ≤18 years of age: LVPWTd ≥2 mm above 2 SD more than normal and/or IVSTd ≥2 mm above 2 SD more than normal (using BSA normal ranges from Kampmann, et al, 2000)
  o Patients >18 years old: left ventricular mass index (LVMI) >50 $g/m^{2.7}$ and/or any parietal wall thickness >12 mm.
• Other parameters indicating Fabry disease is progressing and/or too advanced to keep the patient at low dose Fabrazyme.
Pharmacokinetics: Blood samples will be collected from patients at all sites that have the capability of collecting and processing samples for PK analyses, in accordance with local regulations. Blood samples for PK profiles of Fabrazyme will be collected at Day 1 and at Week 52/Year 1. Samples will be collected at 0 minutes (pre-dose), every hour after the start of the infusion, at the infusion end, and at 15 minutes, 30 minutes, 1 hour, 2 hours, 4 hours, 6 hours, and 8 hours post-infusion and sent to sent to a central laboratory.
Safety: Safety will be evaluated based on physical examinations (performed at Screening and every 3 months through Week 52/Year 1 and every 6 months thereafter); vital signs (measured every visit, pre-infusion and post-infusion); clinical laboratory tests (serum chemistry, hematology, and urinalysis) (performed at Screening and every 3 months through Week 52/Year 1, and once a year thereafter and sent to a central laboratory); electrocardiograms (ECGs) (performed at Screening, Week 52/Year 1, and once a year thereafter and sent to a central laboratory); adverse events (AEs) and concomitant medications and therapies. Serum to determine Immunoglobulin G (IgG) antibody titers to Fabrazyme will be collected on Day 1, every 4 weeks through Week 28/Year 0.5, then every 3 months through Week 52/Year 1 (ie, twice), and every 6 months thereafter and sent to sent to a central laboratory.</td></tr>
<tr><td colspan="3">STATISTICAL METHODS:
Efficacy: Efficacy analyses will be performed on randomized patients receiving one or more infusions of Fabrazyme (full analysis set [FAS] population). The Per Protocol (PP) population will be FAS patients at least 80% compliant with drug dosing and without major protocol violations. The primary endpoint of GL-3 inclusions in the skin vascular endothelium of the skin will be scored as 0 (normal) or 1, 2, or 3 (abnormal). A binomial matched pair procedure will be used to compute a p-value for each treatment group based on shifts from Baseline to the timepoint for a zero-nonzero score. Shift tables and a listing of scores by patient will be produced and graphs may be presented as appropriate. Efficacy data may by summarized stratified by IgG area under the curve (AUC)/titers, as appropriate. Frequencies and percentages will be presented by treatment group for categorical variables. Continuous variables will be summarized by treatment group using descriptive statistics (N, mean, SD, median, range) and/or by summarizing the change from last observation prior to treatment to each visit. As appropriate, changes from Baseline will be summarized by treatment group and graphs may be presented.</td></tr>
</table>

NAME OF COMPANY<br>Genzyme Corporation<br>500 Kendall Street<br>Cambridge, MA 02142<br><br>NAME OF FINISHED PRODUCT<br>Fabrazyme®<br><br>NAME OF ACTIVE INGREDIENT<br>Recombinant Human α-galactosidase A	SUMMARY TABLE<br>Referring to Part ……<br>of the Dossier:<br><br>Volume:<br><br>Page:<br><br>Reference:	FOR NATIONAL AUTHORITY USE ONLY:
**Pharmacokinetics:** All concentration data will be summarized using descriptive statistics at each visit and timepoint by treatment group. All concentrations less than the limit of quantification will be set to missing. Fabrazyme pharmacokinetics will be characterized using compartmental methods within the framework of a nonlinear mixed effects model. For each patient, plasma concentration-time data will be graphically presented. Similarly, graphs of the mean plasma concentration-time profiles and graphs with combined individual plasma concentration-time profiles will be produced by treatment group.<br>**Safety:** All randomized patients who receive at least 1 infusion of Fabrazyme will be included in the safety population. Selected safety parameters will be provided to the DMC and to Global Patient Safety and Risk Management (GPS-RM) on a regular basis. AEs will be summarized by treatment group. Summaries include AEs overall, by severity, by relationship to study medication, infusion-associated reactions (IARs), events leading to discontinuation, and serious adverse events (SAEs). Other safety parameters include vital signs (heart rate, blood pressure, respiratory rate, body temperature), physical examination, laboratory parameters (hematology, chemistry and urinalysis), ECG, and antibody development assessments. Safety variables will be summarized using descriptive statistics (N, mean, SD, median, range for continuous variables; N, % for categorical variables). Change from last pre-treatment observation to timepoint (and/or shifts from last pre-treatment observation to timepoint) will be presented by treatment group as appropriate. Graphical displays may be presented as appropriate.<br>**Planned Interim Analysis:** After all patients have been treated for 1 year, relevant clinical, PK, and safety parameters (including GL-3 clearance evaluation in plasma, urine, and skin; AEs; SAEs; vital signs; IgG titers; and study drug exposure data) may be analyzed.		

PROPRIETARY AND CONFIDENTIAL

2. TABLE OF CONTENTS

1. SYNOPSIS 4
2. TABLE OF CONTENTS 9
3. ABBREVIATIONS AND TERMS 13
4. INTRODUCTION 15
4.1 Summary of Benefits 16
4.2 Summary of Risk 17
5. STUDY OBJECTIVES 19
6. INVESTIGATIONAL PLAN 20
6.1 Study Design 20
6.2 Discussion of Study Design Including Choice of Comparator Group 22
7. PATIENT POPULATION AND SELECTION 23
7.1 Inclusion Criteria 23
7.2 Exclusion Criteria 23
7.3 Home Infusion Criteria 24
7.4 Patient Withdrawal 25
7.4.1 Patient Withdrawal 25
7.4.2 Study or Site Termination 26
7.5 Patient Screening Log 26
8. TREATMENTS 28
8.1 Treatments Administered 28
8.2 Investigational Product 29
8.2.1 Packaging and Labeling 30
8.2.2 Study Treatment Preparation 30
8.2.3 Drug Storage 30
8.2.4 Study Drug Accountability 30
8.3 Method of Assigning Patients to Treatment 30
8.4 Dose Selection 31
8.5 Blinding and Randomization 31
8.6 Treatment Compliance 31
9. EFFICACY, SAFETY, AND PHARMACOKINETIC VARIABLES 33
9.1 Efficacy, Safety, and Pharmacokinetic Measurements Assessed and Study Flowchart 33
9.1.1 Screening Assessments 38
9.1.2 Day One 39
9.1.3 Every-Two-Week Visits 39
9.1.4 Every-Four-Week Visits 40
9.1.5 Week 12/Month 3 41
9.1.6 Week 28/Month 6/Year 0.5 42
9.1.7 Week 40/Month 9 43
9.1.8 Week 52/Year 1 44
9.1.9 Week 80/Year 1.5 45
9.1.10 Week 104/Year 2 46
9.1.11 Week 132/Year 2.5 47
9.1.12 Week 156/Year 3 48

9.1.13 Week 184/Year 3.5 49
9.1.14 Week 208/Year 4 50
9.1.15 Week 236/Year 4.5 51
9.1.16 Week 260/Year 5 (Study Completion) or Early Withdrawal 52
9.1.17 Safety Follow-up 53
9.2 Efficacy Measurements 53
9.2.1 Evaluation of Skin Biopsies 53
9.2.2 Evaluation of Kidney Biopsies 53
9.2.3 GL-3 Evaluations 54
9.2.4 Renal Function 54
9.2.4.1 Glomerular Filtration Rate 55
9.2.4.2 Protein Excretion 55
9.2.5 Ear Function 56
9.2.6 Brain Abnormalities 56
9.2.7 Echocardiograph 57
9.2.8 Retinal Imaging 57
9.2.9 Angiokeratoma 58
9.2.10 Gastrointestinal Symptoms 58
9.2.11 Quality of Life Questionnaires 58
9.2.12 Biomarkers 59
9.2.13 Criteria for Increasing the Study Drug Dose 59
9.3 Pharmacokinetic Profiles 59
9.4 Safety Measurements 60
9.4.1 Physical Examination 60
9.4.2 Vital Signs and Weight 60
9.4.3 Clinical Laboratory Evaluations 61
9.4.3.1 Clinical Laboratory Assessments 61
9.4.3.2 IgG Antibody Testing 62
9.4.3.3 IgE Antibody, Serum Tryptase and Complement Activation Testing 62
9.4.3.4 Skin Testing 63
9.4.3.5 Immune Complex and Inhibitory Antibody Testing 63
9.4.4 Electrocardiogram 63
9.4.5 Adverse Events 64
9.4.5.1 Infusion-Associated Reactions 65
9.4.5.2 Serious Adverse Events 65
9.4.5.3 Adverse Event, Infusion-Associated Reaction, and Serious Adverse Event Reporting 66
9.4.6 Concomitant Medications 67
9.4.6.1 Medications Affecting Creatinine 67
9.4.6.2 Angiotensin Converting Enzyme Inhibitors/Angiotensin Receptor Blockers Therapy 68

9.4.7 Concomitant Therapies 68
9.4.8 Pregnancy 68
9.4.9 Safety Follow-Up 68
9.5 Other Assessments 69
9.5.1 Alpha-Galactosidase A 69
9.5.2 Genotyping 69
9.6 Data Monitoring Committee 69
10. DATA COLLECTION, QUALITY ASSURANCE, AND MANAGEMENT 70
10.1 Recording of Data 70
10.2 Data Quality Assurance 70
10.3 Data Management 70
11. STATISTICAL METHODS AND PLANNED ANALYSES 71
11.1 Study Populations 71
11.2 Power and Sample Size 71
11.3 Missing or Invalid Data 71
11.4 Demographics and Baseline Characteristics 71
11.5 Patient Accountability 71
11.6 Study Drug Treatment Usage and Compliance 71
11.7 Objectives and Endpoints 72
11.7.1 Efficacy Endpoints 72
11.7.1.1 Primary Efficacy Endpoint 72
11.7.1.2 Secondary Efficacy Endpoints 73
11.7.1.3 Exploratory Efficacy Endpoints 73
11.7.2 Pharmacokinetic Endpoints 74
11.7.3 Safety Endpoints 75
11.7.3.1 Vital Signs and Physical Examinations 75
11.7.3.2 Clinical Laboratory Parameters 75
11.7.3.3 Electrocardiogram Measurements 76
11.7.3.4 Antibody Development to Fabrazyme 76
11.7.3.5 Adverse Events 76
11.7.3.6 Concomitant Medications and Therapies 76
11.8 Planned Interim Analysis 76
12. SPECIAL REQUIREMENTS AND PROCEDURES 77
12.1 Institutional and Ethical Review 77
12.2 Changes to the Conduct of the Study or Protocol 77
12.3 Investigator's Responsibilities 77
12.3.1 Patient Informed Assent/Consent 77
12.3.2 Case Report Forms 78
12.3.3 Record Retention 78
12.3.4 Monitoring 78
12.3.5 Materials Control 79
12.3.5.1 Receipt of Clinical Supplies 79
12.3.5.2 Disposition of Unused Clinical Supplies 79
12.3.5.3 Tracking and Disposition of Clinical Supplies 79

Proprietary and Confidential

12.3.5.4 Product Handling and Complaints Reporting 79
12.3.6 Warnings, Precautions, Contraindications 80
12.3.7 Clinical Study Report 80
12.3.8 Disclosure of Data 80
13. REFERENCES 81
14. APPENDICES 84
14.1 Notification Procedures and Guidelines for the Management of Infusion-Associated Reactions 85
14.2 Suggested Guidelines for Management of Infusion-Associated Reactions During the Event 86
14.2.1 Suggested Management of Mild Reactions 87
14.2.2 Suggested Management of Moderate Reactions 87
14.2.3 Suggested Management of Severe Reactions 88
14.2.4 Management of Recurrent Infusion-Associated Reactions 88
14.2.5 Routine Antibody Sampling and Sample Collection and Skin Testing in case of Infusion-Associated Reactions 89
14.3 Tanner Stages 92

LIST OF IN-TEXT TABLES

Table 9-1 Schedule of Study Events for Protocol AGAL06207 Screening Through Week 52/Year 1 34
Table 9-2 Schedule of Study Events for Protocol AGAL06207 Week 54 Through Week 260/Year 5 36

LIST OF IN-TEXT FIGURES

Figure 1 Study Design 21

3. ABBREVIATIONS AND TERMS

Aa	Late diastolic
ACEI/ARB	Angiotensin converting enzyme inhibitor/angiotensin receptor blocker
ACLS	advanced cardiopulmonary resuscitation
AE	Adverse event
αGAL	α-Galactosidase A
AUC	Area under the plasma concentration-time curve
$AUC_{0-\infty}$	Area under the plasma concentration-time curve extrapolated to infinity
BMI	Body mass index
BPI (SF)	Brief Pain Inventory (Short Form)
BP	Blood pressure
BSA	Body surface area
BUN	Blood urea nitrogen
CBC	Complete blood count
CHO	Chinese hamster ovary
CIC	Circulating immune complexes
CFR	Code of Federal Regulations
CI	Confidence interval
CL	Clearance
C_{max}	Maximum plasma concentration
c/o	complains of
CPRS	Clinical Pharmacy Research Services
CS	Clinically significant
DMC	Data Monitoring Committee
DNA	Deoxyribonucleic acid
E	Transmitral
Ea	Early diastolic
ECG	Electrocardiogram
eCRF	Electronic case report form
EDC	Electronic data capture
EF	Ejection fraction
eGFR	Estimated glomerular filtration rate
EMA	European Medicines Agency
ERT	Enzyme replacement therapy
EU	European Union
FAS	Full Analysis Set
FIELD	Fabrazyme®: Intervening Early at a Lower Dose
FLAIR	Fluid attenuated inversion recovery
GCP	Good clinical practice
GFR	Glomerular filtration rate
GI	Gastrointestinal
GL-3	Globotriaosylceramide
GPS-RM	Global Patient Safety and Risk Management (formerly Pharmacovigilance)
Hct	Hematocrit
HEENT	Head, ears, eyes, nose, and throat
Hgb	Hemoglobin
HR	Heart rate
IAR	Infusion-associated reaction
IB	Investigator brochure

ICH	International Conference on Harmonization
ID	Identification
IEC	Independent Ethics Committee
IgE	Immunoglobulin E
IgG	Immunoglobulin G
IRB	Institutional Review Board
IV	Intravenous
IVSTd	End-diastolic interventricular septum thickness
LLT	Lower level term
LM	Light microscopy
LRT	Likelihood ratio test
LVDd	End diastolic left ventricular diameter
LVMI	Left ventricular mass index
LVPWTd	End-diastolic left ventricular posterior wall thickness
MDRD	Modification of Diet in Renal Disease Study
MedDRA	Medical Dictionary for Regulatory Activities
MR angio	Magnetic resonance angiography
MRI	Magnetic resonance imaging
MW	Mean wall (thickness)
NCS	Not clinically significant
NTE	Not to exceed
PK	Pharmacokinetics
PNF	Pregnancy notification form
PO	orally
POF	Pregnancy outcome form
PP	Per protocol
PPQ	Pediatric Pain Questionnaire
PT	Preferred term
q2w	Every 2 weeks
q4w	Every 4 weeks
RBC	Red blood cell
RBP	Retinol Binding Protein
REB	Research Ethics Board
r-hαGAL	Recombinant human α-Galactosidase A (agalsidase beta, Fabrazyme)
Sa	Systolic
SAE	Serious adverse event/serious adverse experience
SC	Subcutaneously
SD	Standard deviation
SGOT	Serum glutamic oxaloacetic transaminase
SGPT	Serum glutamic pyruvic transaminase
SmPC	Summary of Product Characteristics
SOC	System organ class
SOM	Study Operations Manual
SOP	Standard Operating Procedures
TDI	Tissue doppler imaging
TIA	Transient ischemic attack
T_{max}	Time to maximum plasma concentration
US	United States
Vss	Steady-state volume of distribution
WBC	White blood cell

4. INTRODUCTION

The lysosomal hydrolase enzyme, α-galactosidase A (αGAL), is responsible for the metabolism of globotriaosylceramide (GL-3), the enzyme's major glycosphingolipid substrate. In Fabry disease, a familial[1,2,3] (X-linked) inherited deficiency of α-GAL leads to widespread deposition of GL-3, and to a lesser extent, other α-galactoside-containing glycolipids, in the heart[4], kidney[5] liver[6], skin[7], and intestines.[8] This process causes damage to endothelial, perithelial and smooth-muscle cells of the vascular system, glomerular and tubular cells of the kidney, myocardial cells and valvular fibrocytes, epithelial cells of the cornea and ganglion cells of the dorsal root and autonomic nervous system, as well as cortical and brain-stem structures.[9] Therefore, the disease presents as a multi-system disorder, clinical features being typical but highly variable in affected individuals.[10]

Tissue GL-3 accumulation has been documented in children early in life. Previous case reports describing GL-3 in fetal corneal and renal tissue[11] support the fact that extensive accumulation is already significant at the second trimester of gestation. Extensive storage and very low αGAL activities have been observed in the placental tissue of a mother heterozygous for Fabry disease that gave birth to a hemizygous son.[12]

Childhood manifestations of Fabry disease often include, but are not limited to, angiokeratoma, excruciating acral pain, paraesthesia, hypohidrosis, corneal and lenticular opacities, gastrointestinal (GI) symptoms, and elevated urinary protein excretion rate.[13] Although unusual, strokes have been reported in children with Fabry disease.[14,15] Pediatric patients with Fabry disease have a significantly decreased quality of life compared with their healthy peers.[16] The frequently unrecognized symptom complex in pediatric Fabry patients often delays the diagnosis until later in life when cardiac,[17] cerebrovascular,[18] and renal[19] complications of GL-3 accumulation have occurred.[13] The burden of disease, as assessed by the number of organ systems affected, increases with age. Left untreated, progressive glycolipid deposition leads to failure of target organs commonly resulting in death in the third to fifth decades of life.[20]

Because Fabry disease patients have deficient αGAL enzymatic activity, the most direct therapeutic approach is to provide the deficient enzyme through enzyme replacement therapy (ERT). Genzyme Corporation has developed and manufactured a highly purified form of the recombinant human α-galactosidase A (r-hαGAL; Fabrazyme®, agalsidase beta), which is synthesized using recombinant deoxyribonucleic acid (DNA) technology. Fabrazyme has been approved for the treatment of patients with Fabry disease in several markets, including the European Union (EU), United States (US), Australia, Canada, and Japan.

Previous clinical studies with Fabrazyme have shown that early initiation of ERT is essential to obtain the best therapeutic benefit[21,22]; however, the most advantageous dose and the age at which therapy should start are currently unidentified. Early therapeutic initiation, to reduce existing and prevent further GL-3 accumulation, is expected to reduce and/or prevent the progression of a disease that leads to significant morbidity. Due to the treatment regimen burden, e.g., lengthy time of each infusion and the infusion frequency, the current recommended course of therapy (Fabrazyme 1.0 mg/kg every 2 weeks [q2w]) may discourage treatment of children who still present few symptoms of Fabry disease.

This study, (FIELD [Fabrazyme: Intervening Early at a Lower Dose], Protocol AGAL06207), is a randomized, multicenter, multinational, open-label, parallel-group study to examine the efficacy, pharmacokinetics (PK), and safety of 2 alternative low-dose regimens in treatment-naive patients ≥5 and ≤18 years of age with Fabry disease without severe symptoms. This study will assess the effect of Fabrazyme on lowering GL-3 levels in skin, plasma, and urine as well as explore its ability to prevent the occurrence of clinical symptoms. The benefits of these dose regimens are not known. Natural history patient cohorts may be used as comparator groups, as appropriate.

4.1 Summary of Benefits

Pre-clinical proof of concept studies have provided evidence that Fabrazyme is transported into the lysosomes of the skin, liver, kidney, heart, and spleen cells and that the recombinant enzyme metabolizes accumulated GL-3.

A Phase 1/2 dose-ranging study in 15 adults (Protocol FB9702-01), using 0.3 mg/kg, 1.0 mg/kg, or 3.0 mg/kg either every 14 days or every 48 hours for 5 doses, resulted in marked tissue and dose dependent plasma reductions of GL-3. Some patients reported less pain, improved quality of life, and increased sweating. In a Phase 3 study (Protocol AGAL-1-002-98) in 58 adult patients using approximately 1.0 mg/kg q2w and its extension (Protocol AGAL-005-99),[23] Fabrazyme treatment reduced glycosphingolipid deposits from the capillary endothelium of key organs, including the kidney and the heart. The long-term (5-year) follow-up showed stabilization of kidney function in most patients and documented the long-term safety of the treatment.[24] A Phase 4 study (Protocol AGAL-008-00) demonstrated that 1.0 mg/kg q2w of Fabrazyme slowed the progression of Fabry disease in adults as manifested by the reduction of significant renal, cardiac, and cerebrovascular clinical event rates.

A low dose maintenance study (Protocol AGAL-017-01) evaluated the efficacy of 1.0 mg/kg q2w Fabrazyme for 24 weeks followed by a dose of 0.3 mg/kg q2w for 18 months to maintain clearance of GL-3 from the interstitial capillary endothelium of the kidney in 21 adults with

Fabry disease. During the low dose maintenance period, most patients maintained the GL-3 clearance achieved at the end of the 1 mg/kg q2w treatment period. Inadequate clearance and/or recurrence of elevated plasma and urine GL-3 values have been reported in patients who have developed immunoglobulin G (IgG) antibodies.[25,26,27] It is hypothesized that at the lower dose, IgG antibodies to r-hαGAL may play a more pronounced role on the efficacy (GL-3 clearance) in some patients, which would warrant reversion to the higher dose to maintain adequate cellular clearance. Due to the limitations of the study design, no definitive conclusion regarding the clinical efficacy of the dose maintenance regimen could be drawn.

4.2 Summary of Risk

Fabrazyme is a highly purified form of human αGAL, which has been made by recombinant DNA technology. It is well known that immune response may occur following treatment with exogenous human proteins. Although occurring infrequently, serious allergic reactions including anaphylaxis have occurred in patients who have taken medications manufactured by this technology.

In the setting of an endogenous enzyme deficiency, infusion of an exogenous recombinant enzyme to patients naïve to the normal enzyme is expected to frequently cause antibody response to the enzyme. As part of an integrated summary of safety, adverse event (AE) information was pooled from the following 7 studies in which a Fabrazyme dose of 1 mg/kg q2w was used for the duration of the study: Protocols AGAL-006-99, AGAL-007-99, AGAL-1-002-98, AGAL-005-99, AGAL-008-00, AGAL02503, and AGAL-016-01. Safety information based on this pooled analysis showed that the majority (84%) of male patients who received Fabrazyme in completed clinical studies have developed IgG antibodies to Fabrazyme. However, continued treatment with Fabrazyme has not been precluded by IgG antibody development and no significant impact has been observed on clinical efficacy parameters at 1 mg/kg q2w.

The most common drug-related AEs across these 7 pooled clinical studies were infusion-associated reactions (IARs), defined for these studies as drug-related AEs occurring on the day of the infusion. Most IARs were mild to moderate in intensity and non-serious. The AE profile of Fabrazyme in pediatric patients was generally similar to that in adult patients. Most patients who experienced IARs were successfully managed using standard medical practices, such as reduction in infusion rate and/or pre-medication with non-steroidal anti-inflammatory drugs, antipyretics, antihistamines, and/or corticosteroids.

Across these 7 studies, a total of 78 patients experienced 230 Serious Adverse Events (SAEs); the majority of which were unrelated to Fabrazyme therapy. A total of 15 patients experienced 27 Fabrazyme-related SAEs. The drug-related SAEs were mainly IARs and

included angioedema, urticaria, hypotension, hypertension, tachycardia, throat tightness, chest discomfort, pain in extremity, pyrexia, chills, malaise, and nasal congestion. One patient had a reported serious IAR of anaphylactic shock; however, a review of all available information by a consultant allergist suggests the clinical scenario, as reported, did not constitute anaphylactic shock (refer to the Investigator's Brochure (IB) or approved product information for details). A total of 9 patients were identified as having serum immunoglobulin E (IgE) antibodies or a skin test positive to Fabrazyme. At least 8 of these 9 patients have been able to receive subsequent Fabrazyme infusions as part of the original clinical study, in the Rechallenge study (Protocol AGAL-019-01), or commercial use.

The current IB or other approved product information such as the Summary of Product Characteristics (SmPC) provides preclinical, clinical, and post-marketing information regarding the safety and efficacy of Fabrazyme.

Studies have not been completed to assess the potential effects of Fabrazyme on impairment of fertility in humans.

5. STUDY OBJECTIVES

The objectives of this open-label study are to evaluate the efficacy (GL-3 clearance), PK, and safety parameters (including immunogenicity) for 2 alternative dose regimens of Fabrazyme (0.5 mg/kg every 2 weeks [q2w] and 1.0 mg/kg every 4 weeks [q4w]) in treatment-naive male pediatric patients (≥5 years to ≤18 years of age) with Fabry disease without severe symptoms.

6. INVESTIGATIONAL PLAN

6.1 Study Design

This will be a randomized, multicenter, multinational, open-label, parallel-group study to evaluate 2 alternative dose regimens of Fabrazyme (0.5 mg/kg q2w and 1.0 mg/kg q4w) in treatment-naive male pediatric patients with Fabry disease without severe symptoms (see Figure 1).

Approximately 35 male patients ≥5 and ≤18 years of age will receive treatment with Fabrazyme. Patients will be stratified by age at enrollment (5 to 11 years [children] and 12 to 18 years [adolescents]) to allow for a similar age representation between the 2 treatment arms. Patients will then be randomized to receive intravenous (IV) infusions of Fabrazyme at a dose of 0.5 mg/kg q2w or 1.0 mg/kg q4w (up to 131 or 66 infusions, respectively). Patients who meet specific criteria may have the option of receiving infusions at home after Week 28/Month 6/ Year 0.5 for q2w infusion patients and Week 52/Year 1 for q4w infusion patients (see Section 7.3).

The magnetic resonance imaging (MRI) of the brain, kidney biopsy, and retinal imaging are considered optional in this protocol, in order to allow enrollment of patients for whom any of these tests may be unsuitable. The Study Investigator is strongly encouraged to complete these and all required tests with each patient. In this small study, it is important to make every effort to collect as full a spectrum of data as possible for each patient exposed to drug.

Patients will be monitored for Fabry disease progression during the study. In cases of documented and significant progression of Fabry disease, the dose of Fabrazyme may be increased to the approved dosing regimen of 1.0 mg/kg q2w in the patients concerned, after consultation with the Data Monitoring Committee (DMC) and agreement of the Sponsor (see Section 9.2.13). Any patient switched to 1.0 mg/kg q2w will continue to be evaluated in the study.

Each patient will be treated for 5 years (260 weeks) after which patients are encouraged to enroll in the Fabry Registry. The Fabry Registry is a global, observational, and voluntary program for patients with Fabry disease, intended to explore and define the natural course and clinical characteristics of the disease as well as to track and characterize response to treatments.

PROPRIETARY AND CONFIDENTIAL

Figure 1 Study Design

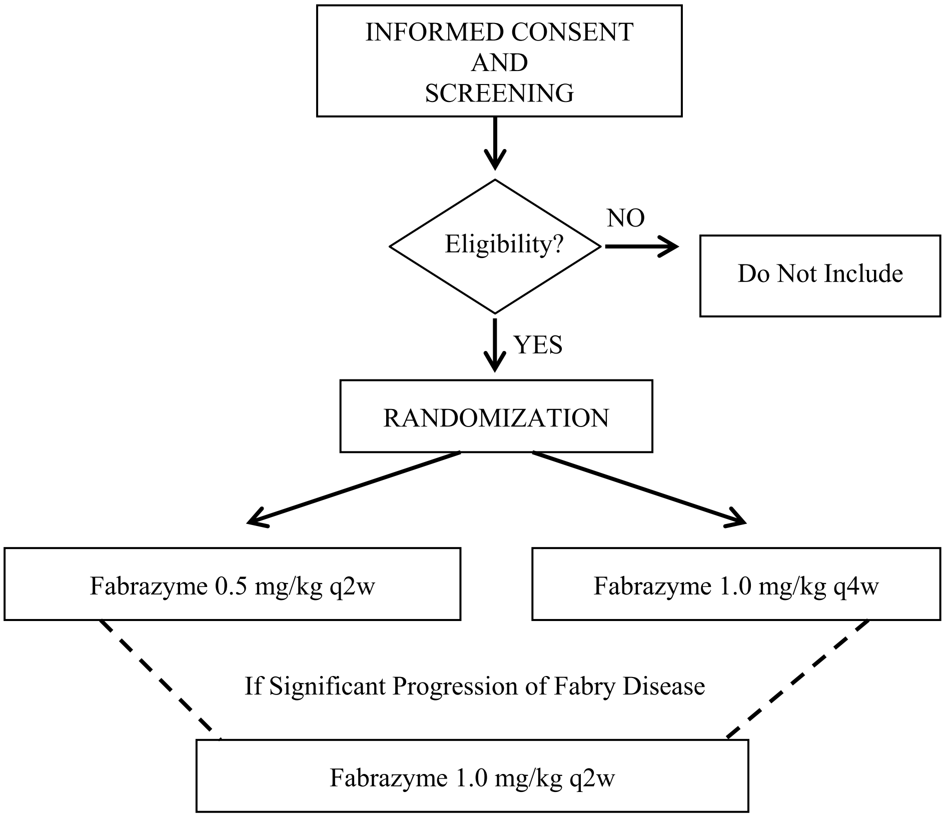

6.2 Discussion of Study Design Including Choice of Comparator Group

Fabry disease is a very rare condition and progresses very slowly; therefore, it is extremely challenging to design a clinical outcome study. This is particularly true in children, in whom a very long study would likely be required to ascertain a measurable difference in clinical parameters such as glomerular filtration rate (GFR) or left ventricular mass index (LVMI). A placebo-controlled study is not desirable in the pediatric population, especially when therapy is available. Therefore, the main objectives are evaluating GL-3 clearance (efficacy), safety, and PK of 2 low-dose regimens that may be suitable in this population.

It is accepted that not all eligible patients are willing/able to undergo kidney biopsy. Therefore, the primary endpoint will be another surrogate marker: the GL-3 clearance from the vascular endothelium of the skin. All other parameters, especially clinical efficacy parameters, are of an exploratory nature. Natural history patient cohorts may be used as comparator groups, as appropriate.

7. PATIENT POPULATION AND SELECTION

Approximately 15 multinational centers will participate in this clinical study.

7.1 Inclusion Criteria

Patients who meet the following inclusion criteria will be eligible for enrollment in this study:

1. The patient and/or patient's parent(s)/legal guardian(s) must provide written informed assent/consent prior to any protocol-related procedures being performed.
2. The patient must have a confirmed diagnosis of Fabry disease as documented by leukocyte αGAL of <4 nmol/hr/mg leukocyte (preferred assay; results from a central laboratory). If the leukocyte αGAL activity assay is difficult to obtain, the patient may be enrolled based on documented plasma αGAL <1.5 nmol/hr/mL, with the agreement of the Medical Monitor (results from a central laboratory).
3. The patient must have evidence of GL-3 accumulation as documented by plasma GL-3 (>7.0 µg/mL) or urinary GL-3 (>0.03 mg GL-3/mmol creatinine) levels (results from a central laboratory).
4. The patient must be male ≥5 and ≤18 years of age.

7.2 Exclusion Criteria

Patients who meet any of the following exclusion criteria will not be eligible for enrollment in this study:

1. Patient has albuminuria (first morning void urinary albumin/creatinine ratio >30 mg/g on at least 2 out of 3 consecutive samples, each at least 1 week apart).
2. Patient has a $GFR_{iohexol}$ <90 mL/min/1.73 m2. In case of properly documented low protein intake, values as low as 80 mL/min/1.73 m2 may be acceptable, after consultation with the Medical Monitor (see Section 9.2.4.1).
3. Patient has documented evidence of stroke or transient ischemic attack (TIA) or, if brain MRI has been performed, bright lesions >2 mm on T2- or fluid attenuated inversion recovery (FLAIR)-weighted images within the white matter or the basal ganglia.
4. Patient has severe and recurrent acroparesthesia, judged by the physician as frequent (more than once a week) pain episodes for at least 3 months that influence daily activities, irrespective of medication.
5. Patient has an end-diastolic left ventricular posterior wall thickness (LVPWTd) and/or an end-diastolic interventricular septum thickness (IVSTd) ≥2 standard deviations (SD) compared to normal based on body surface area (BSA) normal ranges[28] as read at the study site.
6. Patient has received prior treatment specific to Fabry Disease.

PROPRIETARY AND CONFIDENTIAL

7. Patient has participated in a study employing an investigational drug within 30 days of the start of their participation in this study.
8. Patient has any medical condition or extenuating circumstance, which, in the opinion of the Study Investigator, could interfere with study compliance.
9. Patient has any medical condition or extenuating circumstance, e.g., diabetes mellitus, which, in the opinion of the Study Investigator, could interfere with the interpretation of study results.
10. Patient is on treatment with angiotensin converting enzyme inhibitors/angiotensin receptor blockers (ACEIs/ARBs).
11. Patient has any contra-indication mentioned in the labeling of Fabrazyme and/or iohexol (Omnipaque™).
12. Patient or parent(s)/legal guardian(s) is unwilling to comply with the requirements of the protocol.

7.3 Home Infusion Criteria

Patients who meet specific criteria may have the option of receiving study treatment at home after Week 28/Month 6/Year 0.5 for q2w and Week 52/Year 1 for q4w infusion patients. These patients may receive infusions at home if no other study evaluations are scheduled. Patients are required to return to their Study Investigator for study evaluations and, when so doing, will receive their Fabrazyme infusion at the study site.

Specific criteria for home infusion are:

- The Study Investigator, Medical Monitor, and Global Patient Safety and Risk Management (GPS-RM) representative must agree that home infusion is appropriate for the patient and provide written approval.
- The patient must be clinically stable (per the Study Investigator) prior to infusions with
  - ➢ No IARs suggestive of a hypersensitivity reaction (regardless of severity) within the last 6 infusions. Determination of whether an IAR is suggestive of a hypersensitivity reaction will be based on the medical judgment of the Study Investigator or his/her designee.
  - ➢ No worsening of mild IARs unrelated to hypersensitivity reaction within the last 6 infusions.
- The patient must be at the same infusion rate scheme for a minimum of 4 infusions prior to transfer. No infusion rate increases will be allowed while a patient is receiving infusions in the home setting.
- The patient must have no ongoing (not yet recovered) SAEs.

- The patient and parent(s)/legal guardian(s) must be willing and able to comply with home infusion procedures.
- Home infusion infrastructure, resources, and procedures must be established and available according to the applicable regional regulations.

In addition, information on the patient's seroconversion status (if possible) and use of pre-medications within the previous 2 months will be reviewed by the Study Investigator, Medical Monitor, and GPS-RM representative prior to approving the patient for home infusions. If pre-medications need to be changed after transition to home infusions, this will be discussed with the Medical Monitor.

Patients experiencing an IAR (i.e., drug-related AEs occurring on the day of the infusion; during the infusion or following completion of the infusion) may subsequently receive pre-treatment according to the guidelines provided in Appendix 14.2.4. If a patient is approved for home infusions and subsequently experiences an IAR during a home infusion, the home infusion nurse will immediately report the details of the IAR to the Study Investigator (or his/her designee), regardless of the IAR severity. The Study Investigator will assess the severity of the IAR and the relationship to Fabrazyme. Testing for serum tryptase, plasma complement, IgG, and/or IgE will be performed, if appropriate, as described in Section 9.4.3.2, Section 9.4.3.3, and Section 14.2.5). Patients experiencing a moderate or severe IAR will return to the study site for their next infusions until the patient meets the home infusion criteria again, and a written approval has been given for home infusions. Homecare representatives will work with the site personnel to ensure data is transferred and transcribed to the study site in an accurate and timely manner based on procedures described in the country- or site-specific home infusion manual.

Refer to the Investigational Product Handling Manual for further details.

If it is deemed appropriate to increase the Fabrazyme dose to 1 mg/kg q2w (see Section 9.2.13), the patient will return to the study site for infusions until the above criteria are again met.

7.4 Patient Withdrawal

7.4.1 Patient Withdrawal

Patients and/or parent(s)/legal guardian(s) are free to withdraw assent/consent and discontinue participation in the study at any time, and without prejudice to further treatment. A patient's participation in the study may be discontinued at any time at the discretion of the Study Investigator. The following may be justifiable reasons for the Study Investigator to remove a patient from the study:

- The patient and/or parent(s)/legal guardian(s) is uncooperative, including failure to appear at study visits;
- The patient was erroneously included in the study;
- The patient suffers an intolerable AE;
- The study is terminated by the Sponsor; or
- The patient refuses clinical study material administration.

If a patient or parent/legal guardian decides to discontinue participation in the study, he or she should be contacted by the Study Investigator in order to obtain information about the reason(s) for discontinuation and collection of any potential AEs. Whenever possible the patient should return to the study site for the final clinical assessments as specified in Section 9.1.16. The Study Investigator will describe the reason for discontinuation on the appropriate electronic case report form (eCRF). The Study Investigator will encourage the patient to enroll in the Fabry Registry.

7.4.2 Study or Site Termination

If the Sponsor, Study Investigator, Clinical Monitor, or regulatory authorities discover conditions during the study that indicate that the study or study site should be terminated, this action may be taken after appropriate consultation between the Sponsor, Study Investigator, and Clinical Monitor. Conditions that may warrant termination of the study include, but are not limited to the following:

- The discovery of an unexpected, serious, or unacceptable risk to patients enrolled in the study;
- The decision on the part of the Sponsor to suspend or discontinue testing, evaluation, or development of the study drug;
- Failure of the Study Investigator to comply with pertinent regulatory authority regulations;
- Submission of knowingly false information from the research facility to the Sponsor, Clinical Monitor, or regulatory authorities; or
- Insufficient adherence to protocol requirements.

Study termination and follow-up will be performed in compliance with the conditions set forth in applicable local regulations and Sponsor Standard Operating Procedures (SOPs).

7.5 Patient Screening Log

Each study site will be instructed to maintain a log of each patient considered for the study, including patients who are not entered into the study. For all patients screened the following will be recorded:

- The dates of Screening,

- Patient initials,
- The reason for not entering the patient into the study, if applicable,
- The date of assent/consent (if applicable), and
- The patient identification (ID) number.

8. TREATMENTS

8.1 Treatments Administered

Patients will be stratified by age and randomized to treatment with Fabrazyme either 0.5 mg/kg q2w or 1.0 mg/kg q4w (up to 131 or 66 infusions, respectively). Following the first infusion on Day 1, subsequent infusions will be administered relative to Day 1 ±7 days for all patients. For patients randomized to q2w infusions, no 2 infusions should be administered less than 7 days apart. All infusions will initially be administered at the study site. After Week 28/Month 6/Year 0.5 (q2w infusion patients) or Week 52/Year 1 (q4w infusion patients), patients who meet the criteria in Section 7.3 may have the option of receiving infusions at home, but are required to return to their Study Investigator for study evaluations.

The infusions will be administered at an initial rate of no more than 15 mg/hr via an appropriate IV infusion device. If medically appropriate, a semi-permanent IV access port may be inserted for infusions.

After 8 infusions and after patient tolerance has been established, the infusion rate can be increased by 5 mg/hr at each subsequent visit. For patients who are randomized to receive 0.5 mg/kg q2w, the total infusion time should not be less than 45 minutes and for patients who are randomized to receive 1.0 mg/kg q4w the total infusion time should not be less than 90 minutes. The infusions can either be infused at a constant rate or titrated.

Detailed information regarding dosing and administration is provided in the Investigational Product Handling Manual.

Patients are required to remain at the study site for approximately 1 hour after the infusion is completed and the Study Investigator determines the patient is stable. For those patients infused at home, the home infusion nurse must observe the patient for approximately 1 hour after completion of the infusion and until the home infusion nurse determines the patient is stable. All applicable regional regulations and procedures must be followed.

Prior to each infusion, the patient will be assessed by the Study Investigator or designee (or the home infusion nurse for those patients infused at home) to determine if the patient is free of acute illness and is clinically stable to receive the infusion. If the patient is determined to be acutely ill on the scheduled day of infusion, the infusion may be postponed, and the Medical Monitor will be notified as soon as possible.

Patients may be pre-treated with acetaminophen/paracetamol. In addition, patients may be pre-treated with an antihistamine or steroid approved by the Study Investigator. Patients who have experienced symptoms not suggestive of IgE-mediated hypersensitivity (such as fever,

chills, or rigors) may benefit from the administration of ibuprofen instead of acetaminophen/paracetamol (see Appendix 14.2).

In cases of documented and significant progression of Fabry disease (see Section 9.2.13), the dose of Fabrazyme may be increased to the approved dosing regimen of 1.0 mg/kg q2w for the patient concerned, after consultation with the DMC and agreement of the Sponsor. The 1.0 mg/kg infusions should be started at the most recently-tolerated infusion rate. Any patient switched to 1.0 mg/kg q2w will continue to be evaluated in the study. Any patient on home infusion will return to the study site for infusions until they again meet the home infusion criteria.

8.2 Investigational Product

Description:	recombinant human α-galactosidase A (r-hαGAL; Fabrazyme) is produced by mammalian cell culture technology using a Chinese Hamster Ovary (CHO) cell line.
Chemical Name:	recombinant human α-galactosidase A (r-hαGAL)
CAS Registry Number:	104138-64-9
USAN (WHO/INN):	agalsidase beta
Chemical Structure:	r-hαGAL consists of 2 identical subunits. The full-length cDNA for each subunit encodes a polypeptide of 429 amino acids. The mature subunit is a polypeptide of 398 amino acids. The mature protein is glycosylated.
Molecular Weight:	r-hαGAL has a homodimeric structure with a molecular weight of approximately 100 kDa.

Fabrazyme is supplied in single-use, clear Type I 5 mL (5 mg) and 20 mL (35 mg) glass vials.

Each 20 mL (35 mg) vial contains 37 mg of agalsidase beta as well as 222 mg mannitol, 20.4 mg sodium phosphate monobasic monohydrate, and 59.2 mg sodium phosphate dibasic heptahydrate. Following reconstitution as directed, 35 mg of agalsidase beta (7 mL) may be extracted from each vial.

Each 5 mL (5 mg) vial contains 5.5 mg of agalsidase beta as well as 33.0 mg mannitol, 3.0 mg sodium phosphate monobasic monohydrate, and 8.8 mg sodium phosphate dibasic heptahydrate. Following reconstitution as directed, 5 mg of agalsidase beta (1 mL) may be extracted from each vial.

8.2.1 Packaging and Labeling

Packaging and labeling of the investigational product for all clinical sites will be coordinated by Clinical Pharmacy Research Services (CPRS). Label text will minimally include the contents of the vial (i.e., Fabrazyme 35 mg or 5 mg), lot number, storage conditions, the Sponsor's name and address, and other text as required by region. Under no circumstance will the study medication be used other than as directed by the protocol.

8.2.2 Study Treatment Preparation

The investigational product, Fabrazyme, will be supplied in either 5 mL (5 mg/vial) or 20 mL vials (35 mg/vial) as a lyophilized preparation. Each 5 mg vial and 35 mg vial will be reconstituted with 1.1 mL and 7.2 mL of sterile water for injection, respectively. The reconstituted dose will then be further diluted with a 0.9% sodium chloride solution to a final total volume appropriate for the child's weight. The patient will be weighed at each visit and that weight will be used to calculate the infusion dose to be administered at that visit. The weight taken at the previous visit may be used if current weight is not available at the time of dose preparation. Dilution guidelines are provided in the Investigational Product Handling Manual and must be followed.

8.2.3 Drug Storage

All clinical drug supplies must be kept in a secure, temperature-controlled location with restricted access at 2° to 8°C.

8.2.4 Study Drug Accountability

The Study Investigator or designee must maintain an adequate record of the receipt and distribution of all study medication using a drug accountability form. This form must be available for inspection at any time. The investigational product may only be used in accordance with this approved protocol and must not be used for any other purpose. Investigational product may only be used for patients who have provided written informed assent/consent to participate in this study and meet all study entrance criteria and who do not meet any of the withdrawal criteria during the study.

If any unused material remains at the site upon completion of the study, the pharmacy will be instructed to destroy or return the material to the Sponsor.

8.3 Method of Assigning Patients to Treatment

Upon confirmation that the patient meets all eligibility criteria and completion of the Screening assessments, eligible patients will be randomized as described in Section 8.5.

Approximately 35 patients will be enrolled. Each patient will be assigned a 4-digit ID number. The first 2 digits will correspond to the pre-assigned study site number (e.g., 01XX,

02XX, etc.), while the second 2 digits will correspond to the sequential order in which each patient is screened at that particular study site (e.g., first patient screened at first study site = 0101, first patient screened at second study site = 0201, etc.).

8.4 Dose Selection

Many pediatric patients with Fabry Disease are not treated with ERT. A major reason for this is the significant burden of treatment, which consists of every 2 weeks IV infusions taking 2 to 4 hours. Decreasing this burden in the pediatric population might allow more of these patients to have access to treatment at an earlier stage of this progressive disease and prior to the onset of irreversible damage.

The Phase 1/2 study (Protocol FB9702-01) showed a dose dependent GL-3 clearance from various body compartments, especially when comparing plasma GL-3 clearance obtained with 0.3, 1 and 3 mg/kg q2w Fabrazyme infusions. The dose-maintenance study (Protocol AGAL-017-01) showed that (after 6 months treatment with 1.0 mg/kg q2w), a lower dose of Fabrazyme (0.3 mg/kg q2w) was not sufficient to maintain biomarker control in all patients and 6 of the 21 patients showed significant re-accumulation of GL-3 in the skin capillary endothelium. It is believed that a higher (0.5 mg/kg) dose would provide a wider margin for confidence in efficacy while still providing the convenience in this population.

The second alternative dosing regimen (1.0 mg/kg q4w) provides the same overall quantity of drug as the first treatment arm, but explores the convenience of less frequent dosing. This dosing regimen could lead to a substantial change in the way children with Fabry Disease are managed.

8.5 Blinding and Randomization

This is an open-label study; therefore, no blinding techniques will be used.

Patients will be stratified by age (5 to 11 years [children] and 12 to 18 years [adolescents]) to allow for a similar age representation between the 2 treatment arms. Eligible patients will then be randomized in a 1:1 ratio to receive an IV infusion of either 0.5 mg/kg q2w or 1.0 mg/kg q4w. A randomization scheme will be developed by the Sponsor. See the Investigational Product Handling Manual for details of the randomization procedure. Patients will be assigned a randomization number after they complete Screening and are deemed eligible for enrollment.

8.6 Treatment Compliance

The patient's compliance with the treatment regimen will be monitored. Reasons for missed or incomplete infusions will be clearly documented.

Noncompliance is defined as missing 3 consecutive infusions for patients on the q2w regimen and 2 consecutive infusions for patients on the q4w regimen or more than 20% of infusions (i.e., more than 1 out of any consecutive 5 scheduled infusions) for any patient. As they are identified, the Study Investigator should discuss noncompliant patients with the Medical Monitor.

PROPRIETARY AND CONFIDENTIAL

9. EFFICACY, SAFETY, AND PHARMACOKINETIC VARIABLES

9.1 Efficacy, Safety, and Pharmacokinetic Measurements Assessed and Study Flowchart

Table 9-1 (Screening through Week 52/Year 1) and Table 9-2 (Week 54 through Study Completion/Week 260/Year 5/Early Withdrawal) summarizes the schedule of study events at each visit. Weeks/Years for each study visit will be based on the number of calendar days relative to enrollment in the study (Day 1).

Study assessment visits will be scheduled based on the date of the first infusion (i.e., Day 1). Between Day 1 and Week 52/Year 1, study assessment visits have a ±7 day window, and after the Year 1 visit, the study assessment visits have a ±14 day window. Infusions will be administered ±7 days relative to Day 1; for patients randomized to q2w infusions, no 2 infusions should be administered less than 7 days apart. The Medical Monitor should be consulted regarding any infusions that may be missed or delayed beyond the specified time windows and to determine whether an alternate infusion date should be scheduled. Subsequent infusions should be scheduled to progressively return to the original visit and infusion schedules.

Table 9-1 Schedule of Study Events for Protocol AGAL06207 Screening Through Week 52/Year 1

	Screening[1]	Day 1	Every 2 Weeks[2,3]	Every 4 Weeks[2,3]	Week 12[2]/ Month 3	Week 28[2]/ Month 6 Year 0.5	Week 40[2]/ Month 9	Week 52[2]/ Year 1
**Written Informed Assent/Consent**	X							
**Inclusion/Exclusion Criteria**	X							
**Fabry Medical History**	X							
**Medical/Surgical History**	X							
**Physical Examination (includes height)**	X				X	X	X	X
**Vital Signs (includes weight)[4]**	X	X	X	X	X	X	X	X
**Hematology and Chemistry Laboratory Tests[5]**	X				X	X	X	X
**αGAL Activity[5]**	X							
**Genotyping for αGAL Gene Mutation[5,6]**	X							
**Plasma GL-3[5]**	X				X	X	X	X
**$GFR_{iohexol}$[5,7]**	X							X
**24-Hour Urine Collection for Low Protein Intake (optional) [5,8]**	X							
**Serum for r-hαGAL IgG Antibody Titers[5,9]**		X		X	X	X	X	X
**Biomarkers[5,10]**	X	X			X	X	X	X
**Urinalysis[5]**	X				X	X	X	X
**First Morning Urine for Total Protein, Albumin, RBP, β_2-microglobulin, and Creatinine[5,11]**	X					X		X
**Urine GL-3[5]**	X				X	X	X	X
**Audiology Examination**	X							X
**Skin Biopsy[5,12]**	X							X
**Kidney Biopsy (optional)[5,13]**	X							
**MRI of Brain (optional)[5]**	X							
**Echocardiograph[5]**	X							X
**Retinal Imaging (optional)[5]**	X							
**Angiokeratoma Digital Photograph(s)[14]**	X							
**GI Symptoms Assessment**		X			X	X	X	X
**PedsQL Questionnaires[15] or BPI (SF)[16]**		X				X		X
**ECG[5]**	X							X
**Enrollment/Randomization**	X							
**Pre-treatment with Antipyretic and/or Antihistamine (optional)**		X	X	X	X	X	X	X
**Infusion of Fabrazyme**		X	X	X	X	X	X	X
**PK Testing[5, 17]**		X						X
**AE Assessment**	←→ **CONTINUOUS MONITORING** ←→							
**Concomitant Medications/Therapies Review**	←→ **CONTINUOUS MONITORING** ←→							

[1] Screening procedures must be completed prior to Day 1 and must be performed within 45 days of Day 1.

[2] Between Day 1 and Week 52/Year 1, study assessment visits have a ±7 day window, and after the Year 1 visit, the study assessment visits have a ±14 day window. Infusions will be administered ±7 days relative to Day 1; for patients randomized to q2w infusions, no 2 infusions should be administered less than 7 days apart. The Medical Monitor should be consulted regarding any infusions that may be missed or delayed beyond the specified time windows and to determine whether an alternate infusion date should be scheduled. Subsequent infusions scheduled to progressively return according to the original visit and infusion schedules.

[3] Patients randomized to q4w infusions **DO NOT** come to the study site for the q2w visits.

[4] Vital signs (blood pressure [BP], heart rate [HR], respiratory rate, and temperature) are assessed both before and 1 hour ±15 minutes after completion of each infusion. Weight is measured before each infusion.

[5] These tests are analyzed/read at a central laboratory.

[6] If genotype is unavailable, a sample is to be sent to a central laboratory for testing.

[7] Iohexol plasma clearance testing must not be performed on the same day as a Fabrazyme infusion or PK sampling.

[8] A 24-hour urine collection for low protein intake may be obtained for determination of protein intake in patients who may have a low $GFR_{iohexol}$ result due to low protein intake (see Section 9.2.4.1).

[9] Antibody collections for IgG analysis will be collected prior to infusions at Day 1 and every 4 weeks for all patients for the first 6 months of the study. Thereafter, they will be collected every 3 months (i.e., twice) and thereafter every 6 months. Refer to Section 9.4.3.2, Section 9.4.3.3, and Section 14.2.5 regarding testing for IgG, IgE, serum tryptase, and/or plasma complement activation for patients who experience moderate to severe or recurrent IARs.

[10] Biomarkers will be assessed from blood and urine samples. No genetic testing will be performed on these samples.

[11] Collect 3 urine samples, each at least 1 week apart. (***Note***: all 3 samples must be collected regardless of the results and collected prior to visit infusion.)

[12] A skin biopsy will be performed at any time after randomization and prior to the first infusion on Day 1.

[13] Optional kidney biopsy will be performed after randomization but prior to Day 1. An additional kidney biopsy is recommended prior to dose increase if justified based on renal findings as defined in the dose increase section.

[14] Angiokeratoma and any changes thereof will be fully described at each physical examination. Digital photograph(s) of angiokeratoma (if present) will be taken at Screening and at any new occurrence/change at any time.

[15] All patients will complete age-appropriate versions of the PedsQL Pediatric or Young Adult Quality of Life Inventory, the PedsQL Multidimensional Fatigue Scale, and the PedsQL Pediatric Pain Questionnaire (PPQ) or Brief Pain Inventory (Short Form) (BPI [SF]).

[16] Patients will switch from the PedsQL Pediatric Quality of Life Inventory to the PedsQL Young Adult QL Inventory and from PedsQL PPQ to the BPI (SF) questionnaire upon reaching 18 years of age.

[17] Samples scheduled from 0 minutes (pre-dose) through 1 hour post-infusion must be collected within ±5 minutes of the nominal timepoint, and samples scheduled from 2 to 8 hours post-infusion must be collected within ±10 minutes of the nominal timepoint.

Table 9-2 Schedule of Study Events for Protocol AGAL06207 Week 54 Through Week 260/Year 5

	Every 2 Weeks[1,2]	Every 4 Weeks[1,2]	Week 80/ Year 1.5[1]	Week 104/ Year 2[1]	Week 132/ Year 2.5[1]	Week 156/ Year 3[1]	Week 184/ Year 3.5[1]	Week 208/ Year 4[1]	Week 236/ Year 4.5[1]	Study Completion[3] Week 260/Year 5[1]/ Early Withdrawal[4]
**Physical Examination (including height)**			X	X	X	X	X	X	X	X
**Vital Signs (including weight)[5]**	X	X	X	X	X	X	X	X	X	X
**Hematology and Chemistry Laboratory Tests[6,7]**				X		X		X		X
**Serum Creatinine[6,7] (for eGFR)**			X		X		X		X	
**Plasma GL-3[6]**			X	X	X	X	X	X	X	X
**$GFR_{iohexol}$[6,8]**				X		X		X		X
**Serum for r-hαGAL IgG Antibody Titers[6,9]**			X	X	X	X	X	X	X	X
**Biomarkers[6,10]**			X	X	X	X	X	X	X	X
**Urinalysis[6]**				X		X		X		X
**First Morning Urine for Total Protein, Albumin, RBP, β_2-Microglobulin, and Creatinine[6,11]**			X	X	X	X	X	X	X	X
**Urine GL-3[6]**			X	X	X	X	X	X	X	X
**Audiology Examination**				X		X		X		X
**Skin Biopsy[6]**						X				X
**Kidney biopsy (optional)[6,12]**										X
**MRI of brain (optional)[6]**					X					X
**Echocardiograph[6]**				X		X		X		X
**Retinal Imaging (optional)[6]**					X					X
**Angiokeratoma Digital Photograph(s)[13]**					X					X
**GI Symptoms**			X	X	X	X	X	X	X	X
**PedsQL Questionnaires[14] or BPI (SF)[15]**			X	X	X	X	X	X	X	X
**ECG[6]**				X		X		X		X
**Pretreatment with Antipyretic and/or Antihistamine (optional)**	X	X	X	X	X	X	X	X	X	X
**Infusion of Fabrazyme**	X	X	X	X	X	X	X	X	X	X
**AE Assessment**	←——— CONTINUOUS MONITORING ———→									
**Concomitant Medications/ Therapies Review**	←——— CONTINUOUS MONITORING ———→									

[1] Infusions will be administered ±7 days relative to Day 1; for patients randomized to q2w infusions, no 2 infusions should be administered less than 7 days apart. The Medical Monitor should be consulted regarding any infusions that may be missed or delayed beyond the specified time windows and to determine whether an alternate infusion date should be scheduled. Subsequent infusions scheduled to progressively return be administered according to the original visit and infusion schedules.
[2] Patients randomized to q4w infusions **DO NOT** come to the study site for the q2w visits.
[3] If possible, a follow-up telephone call to assess AEs and concomitant medication/therapy use will occur approximately 4 weeks after all procedures have been completed. After Study Completion (Week 260/Year 5), patients will be encouraged to enter the Fabry registry.
[4] If possible, this visit should occur within 28 days following the patient's early withdrawal from the study.
[5] Vital signs (blood pressure [BP], heart rate [HR], respiratory rate, and temperature) are taken both before and 1 hour ±15 minutes after completion of each infusion. Weight is measured before each infusion.
[6] These tests are analyzed/read at a central laboratory.
[7] Serum creatinine will be measured every 6 months throughout the study. It is included in the serum chemistry panel at the yearly visits.
[8] Iohexol plasma clearance testing must not be performed on the same day as a Fabrazyme infusion.
[9] Antibody collections for IgG analysis will be collected prior to infusions every 6 months. Refer to Section 9.4.3.2, Section 9.4.3.3, and Section 14.2.5 regarding testing for IgG, IgE, serum tryptase, and/or plasma complement activation for patients who experience moderate to severe or recurrent IARs.
[10] Biomarkers will be assessed from blood and urine samples. No genetic testing will be performed on these samples.
[11] Collect 3 urine samples, each at least 1 week apart. (***Note***: all 3 samples must be collected regardless of the results and collected prior to visit infusion.)
[12] Optional kidney biopsy will be performed after randomization but prior to Day 1. An additional kidney biopsy is recommended prior to dose increase if justified based on renal findings.
[13] Angiokeratoma and any changes thereof will be fully described at each physical examination. Digital photograph(s) of angiokeratoma (if present) will be taken at Week 132/Year 2.5, and Week 260/Year 5 and at any new occurrence/change at any time.
[14] All patients will complete age-appropriate versions of the PedsQL Pediatric or Young Adult Quality of Life Inventory, the PedsQL Multidimensional Fatigue Scale, and the PedsQL PPQ or BPI (SF).
[15] Patients will switch from the PedsQL Pediatric Quality of Life Inventory to the PedsQL Young Adult QL Inventory and from PedsQL PPQ to the BPI (SF) questionnaire upon reaching 18 years of age.

9.1.1 Screening Assessments

The following Screening assessments must be completed within 45 days prior to Day 1. Patients who meet all inclusion/exclusion criteria (as defined by Section 7.1 and Section 7.2) will be enrolled into the study after all required procedures are completed.

- Obtain written informed assent/consent from parent(s)/legal guardian(s) and study participant (if appropriate) prior to any study-related procedures.
- Review inclusion and exclusion criteria.
- Obtain Fabry medical history.
- Obtain medical/surgical history.
- Conduct a physical examination and measure height and describe angiokeratoma (if present) and any changes thereof.
- Conduct a vital signs assessment and measure weight.
- Collect blood samples for clinical laboratory hematology and chemistry assessments.
- Collect leukocyte and plasma samples for αGAL activity.
- Collect a sample for genotyping (if result not already available).
- Collect blood and urine samples for biomarker assessment.
- Collect plasma sample for plasma GL-3.
- Conduct injection and sample collection for GFR calculation via iohexol plasma clearance.
- Collect a 24-hour urine sample to determine protein intake (optional).
- Collect a urine sample for routine urinalysis.
- Collect 3 consecutive first voided morning urine samples, each at least 1 week apart, for total protein, albumin, retinol binding protein (RBP), β_2-microglobulin, and creatinine levels. (***Note:*** all 3 samples must be collected regardless of the results and collected prior to visit infusion.)
- Collect a urine sample for urine GL-3.
- Conduct the audiology examination.
- Obtain a skin biopsy after randomization and prior to the first infusion on Day 1.
- Obtain a kidney biopsy (optional) after randomization but prior to Day 1.
- Perform brain MRI (optional).
- Perform an echocardiograph.
- Take retinal imaging photographs (optional).
- Take angiokeratoma photograph(s), if present.
- Perform an electrocardiogram (ECG).
- Enroll/randomize to either q2w or q4w infusions.
- Collect AEs from the time of signing informed assent/consent.

- Record concomitant medications/therapies, from the time of signing informed assent/consent.

9.1.2 Day One

The following assessments/treatment will be completed at Day 1 (all assessments begin prior to the Fabrazyme infusion):

- Conduct vital signs assessments. Measurement of blood pressure [BP], heart rate [HR], respiratory rate, and temperature will be performed before the infusion and 1 hour ±15 minutes after the completion of the infusion.
- Measure weight before the infusion. (***Note***: the weight measurement of the current visit will be used to calculate the infusion dose of Fabrazyme [in mg] to be administered at that visit. The weight at the preceding visit may be used if current weight is not available at the time of dose preparation.)
- Collect serum for predose r-hαGAL IgG antibody titers.
- Collect blood and urine samples for biomarker assessment.
- Assess GI symptoms.
- Administer age-appropriate self-report instruments: PedsQL Pediatric or Young Adult Quality of Life Inventory, fatigue (PedsQL Multidimensional Fatigue Scale) and pain (PedsQL Pediatric Pain Questionnaire [PPQ] or Brief Pain Inventory (Short Form) (BPI [SF]) if 18 years or older).
- Pre-treat with an antipyretic/antihistamine (optional).
- Administer infusion of Fabrazyme.
- Obtain samples for PK.
- Assess AEs.
- Record concomitant medications/therapies.

Patients are required to remain at the study site for approximately 1 hour after the infusion and until the Study Investigator determines the patient is stable.

9.1.3 Every-Two-Week Visits

The following assessments/treatment will be completed at each visit, i.e., q2w (all assessments begin prior to the Fabrazyme infusion):

- Conduct vital signs assessments. Measurement of BP, HR, respiratory rate, and temperature will be performed before the infusion and 1 hour ±15 minutes after the completion of the infusion.
- Measure weight before the infusion. (***Note***: the weight measurement of the current visit will be used to calculate the infusion dose of Fabrazyme [in mg] to be administered at

that visit. The weight at the preceding visit may be used if current weight is not available at the time of dose preparation.)

- Pre-treat with an antipyretic/antihistamine (optional).
- Administer infusion of Fabrazyme for those patients randomized to q2w infusions.
- Assess AEs.
- Record concomitant medications/therapies.

For patients randomized to q2w infusions, these visits start after Day 1 and occur q2w days for the total study duration of 260 weeks/5 years as illustrated below.

Week	(D1)	2	4	6	8	10	12	14	16	18	20	22	24	26
Month							3							
Week		28	30	32	34	36	38	40	42	44	46	48	50	52
Month		6						9						
Year		0.5												1
Week		54	56	58	60	62	64	66	68	70	72	74	76	78
Year														
Week		80	82	84	86	88	90	92	94	96	98	100	102	104
Year		1.5												2
Week		106	108	110	112	114	116	118	120	122	124	126	128	130
Year														
Week		132	134	136	138	140	142	144	146	148	150	152	154	156
Year		2.5												3
Week		158	160	162	164	166	168	170	172	174	176	178	180	182
Year														
Week		184	186	188	190	192	194	196	198	200	202	204	206	208
Year		3.5												4
Week		210	212	214	216	218	220	222	224	226	228	230	232	234
Year														
Week		236	238	240	242	244	246	248	250	252	254	256	258	260
Year		4.5												5

Month 3 and X.5 Year Visits are adjusted to match the visit timepoints of the q4w patients. D = Day

Patients are required to remain at the study site for approximately 1 hour after the infusion and until the Study Investigator determines the patient is stable.

9.1.4 Every-Four-Week Visits

The following assessments/treatment will be completed q4w (all assessments begin prior to the Fabrazyme infusion):

- Conduct vital signs assessments. Measurement of BP, HR, respiratory rate, and temperature will be performed before the infusion and 1 hour ±15 minutes after the completion of the infusion.

- Measure weight before the infusion. (***Note***: the weight measurement of the current visit will be used to calculate the infusion dose of Fabrazyme [in mg] to be administered at that visit. The weight at the preceding visit may be used if current weight is not available at the time of dose preparation.)
- Obtain serum for r-hαGAL antibody titers (every 4 weeks for the first 6 months (through Week 28/Year 0.5), then every 3 months for 6 months (i.e., twice; through Week 52/Year 1) and then every 6 months thereafter.
- Pretreat with an antipyretic/antihistamine (optional).
- Administer infusion of Fabrazyme for all patients.
- Assess AEs.
- Record concomitant medications/therapies.

Patients randomized to q4w infusions will **not** come to the study site on the q2w visits occurring between the q4w infusion visits. For patients randomized to q4w infusions, these visits start after Day 1 and occur q4w days for the total study duration of 260 weeks/5 years as illustrated below.

Week	(D1)	4	8	12	16	20	24	28	32	36	40	44	48	52
Month				3				6			9			
Year								0.5						1
Week		56	60	64	68	72	76	80	84	88	92	96	100	104
Year								1.5						2
Week		108	112	116	120	124	128	132	136	140	144	148	152	156
Year								2.5						3
Week		160	164	168	172	176	180	184	188	192	196	200	204	208
Year								3.5						4
Week		212	216	220	224	228	232	236	240	244	248	252	256	260
Year								4.5						5

D = Day

Patients are required to remain at the study site for approximately 1 hour after the infusion and until the Study Investigator determines the patient is stable.

9.1.5 Week 12/Month 3

The following assessments /treatment will be completed at 12 weeks for all patients (Week 12/Month 3) (all assessments begin prior to the Fabrazyme infusion):

- Conduct a physical examination and measure height.
- Conduct vital signs assessments. Measurement of BP, HR, respiratory rate, and temperature will be performed before the infusion and 1 hour ±15 minutes after the completion of the infusion.

- Measure weight before the infusion. (***Note***: the weight measurement of the current visit will be used to calculate the infusion dose of Fabrazyme [in mg] to be administered at that visit. The weight at the preceding visit may be used if current weight is not available at the time of dose preparation.)
- Collect blood samples for clinical laboratory hematology and chemistry assessments.
- Collect plasma sample for plasma GL-3.
- Collect serum for r-hαGAL antibody titers.
- Collect blood and urine samples for biomarker assessment.
- Collect a urine sample for routine urinalysis.
- Collect a urine sample for urine GL-3.
- Assess GI symptoms.
- Pre-treat with an antipyretic/antihistamine (optional).
- Administer infusion of Fabrazyme.
- Assess AEs.
- Record concomitant medications/therapies.

Patients are required to remain at the study site for approximately 1 hour after the infusion and until the Study Investigator determines the patient is stable.

9.1.6 Week 28/Month 6/Year 0.5

The following assessments/treatment will be completed at 6 months for all patients (Week 28/Month 6/Year 0.5) (all assessments begin prior to the Fabrazyme infusion):

- Conduct a physical examination and measure height.
- Conduct vital signs assessments. Measurement of BP, HR, respiratory rate, and temperature will be performed before the infusion and 1 hour ±15 minutes after the completion of the infusion.
- Measure weight before the infusion. (***Note***: the weight measurement of the current visit will be used to calculate the infusion dose of Fabrazyme [in mg] to be administered at that visit. The weight at the preceding visit may be used if current weight is not available at the time of dose preparation.)
- Collect blood samples for clinical laboratory hematology and chemistry assessments.
- Collect plasma sample for plasma GL-3.
- Collect serum for r-hαGAL antibody titers.
- Collect blood and urine samples for biomarker assessment.
- Collect a urine sample for routine urinalysis.
- Collect 3 consecutive first voided morning urine samples, each at least 1 week apart, for total protein, albumin, retinol binding protein (RBP), β_2-microglobulin, and creatinine

levels. (***Note:*** all 3 samples must be collected regardless of the results and collected prior to visit infusion.)

- Collect a urine sample for urine GL-3.
- Assess GI symptoms.
- Administer age-appropriate self-report instruments: PedsQL Pediatric or Young Adult Quality of Life Inventory, fatigue (PedsQL Multidimensional Fatigue Scale) and pain (PedsQL PPQ or BPI [SF] if 18 years or older).
- Pre-treat with an antipyretic/antihistamine (optional).
- Administer infusion of Fabrazyme.
- Assess AEs.
- Record concomitant medications/therapies.

Patients are required to remain at the study site for approximately 1 hour after the infusion and until the Study Investigator determines the patient is stable.

9.1.7 Week 40/Month 9

The following assessments/treatment will be completed at 40 weeks for all patients (Week 40/Month 9) (all assessments begin prior to the Fabrazyme infusion):

- Conduct a physical examination and measure height.
- Conduct vital signs assessments. Measurement of BP, HR, respiratory rate, and temperature will be performed before the infusion and 1 hour ±15 minutes after the completion of the infusion.
- Measure weight before the infusion. (***Note***: the weight measurement of the current visit will be used to calculate the infusion dose of Fabrazyme [in mg] to be administered at that visit. The weight at the preceding visit may be used if current weight is not available at the time of dose preparation.)
- Collect blood samples for clinical laboratory hematology and chemistry assessments.
- Collect plasma sample for plasma GL-3.
- Collect serum for r-hαGAL antibody titers.
- Collect blood and urine samples for biomarker assessment.
- Collect a urine sample for routine urinalysis.
- Collect a urine sample for urine GL-3.
- Assess GI symptoms.
- Pre-treat with an antipyretic/antihistamine (optional).
- Administer infusion of Fabrazyme.
- Assess AEs.
- Record concomitant medications/therapies.

Patients are required to remain at the study site for approximately 1 hour after the infusion and until the Study Investigator determines the patient is stable. For those q2w infusion patients infused at home, the home infusion nurse must observe the patient for approximately 1 hour after completion of the infusion and until the home infusion nurse determines the patient is stable. All applicable regional regulations and procedures must be followed.

9.1.8 Week 52/Year 1

The following assessments/treatment will be completed at 1 year for all patients (Week 52/Year 1) (all assessments begin prior to the Fabrazyme infusion):

- Conduct a physical examination and measure height.
- Conduct vital signs assessments. Measurement of BP, HR, respiratory rate, and temperature will be performed before the infusion and 1 hour ±15 minutes after the completion of the infusion.
- Measure weight before the infusion. (***Note***: the weight measurement of the current visit will be used to calculate the infusion dose of Fabrazyme [in mg] to be administered at that visit. The weight at the preceding visit may be used if current weight is not available at the time of dose preparation.)
- Collect blood samples for clinical laboratory hematology and chemistry assessments.
- Collect plasma sample for plasma GL-3.
- Conduct injection and sample collection for GFR calculation via iohexol plasma clearance (must not be on the same day as the Fabrazyme infusion and PK sampling).
- Collect serum for r-hαGAL antibody titers.
- Collect blood and urine samples for biomarker assessment.
- Collect a urine sample for routine urinalysis.
- Collect 3 consecutive first voided morning urine samples, each at least 1 week apart, for total protein, albumin, RBP, β_2-microglobulin, and creatinine levels. (***Note:*** all 3 samples must be collected regardless of the results and collected prior to visit infusion.)
- Collect a urine sample for urine GL-3.
- Conduct the audiology examination.
- Obtain a skin biopsy.
- Perform an echocardiograph.
- Assess GI symptoms.
- Administer age-appropriate self-report instruments: PedsQL Pediatric or Young Adult Quality of Life Inventory, fatigue (PedsQL Multidimensional Fatigue Scale) and pain (PedsQL PPQ or BPI [SF] if 18 years or older).
- Perform an ECG.
- Pre-treat with an antipyretic/antihistamine (optional).

- Administer infusion of Fabrazyme.
- Obtain samples for PK
- Assess AEs.
- Record concomitant medications/therapies.

Patients are required to remain at the study site for approximately 1 hour after the infusion and until the Study Investigator determines the patient is stable. For those patients infused at home, the home infusion nurse must observe the patient for approximately 1 hour after completion of the infusion and until the home infusion nurse determines the patient is stable. All applicable regional regulations and procedures must be followed.

9.1.9 Week 80/Year 1.5

The following assessments/treatment will be completed at 1.5 years (Week 80/Year 1.5) for all patients (all assessments begin prior to the Fabrazyme infusion):

- Conduct a physical examination and measure height.
- Conduct vital signs assessments. Measurement of BP, HR, respiratory rate, and temperature will be performed before the infusion and 1 hour ±15 minutes after the completion of the infusion.
- Measure weight before the infusion. (***Note***: the weight measurement of the current visit will be used to calculate the infusion dose of Fabrazyme [in mg] to be administered at that visit. The weight at the preceding visit may be used if current weight is not available at the time of dose preparation.)
- Collect a blood sample for serum creatinine.
- Collect plasma sample for plasma GL-3.
- Collect serum for r-hαGAL antibody titers.
- Collect blood and urine samples for biomarker assessment.
- Collect 3 consecutive first voided morning urine samples, each at least 1 week apart, for total protein, albumin, RBP, β_2-microglobulin, and creatinine levels. (***Note:*** all 3 samples must be collected regardless of the results and collected prior to visit infusion.)
- Collect a urine sample for urine GL-3.
- Assess GI symptoms.
- Administer age-appropriate self-report instruments: PedsQL Pediatric or Young Adult Quality of Life Inventory, fatigue (PedsQL Multidimensional Fatigue Scale) and pain (PedsQL PPQ or BPI [SF] if 18 years or older).
- Pre-treat with an antipyretic/antihistamine (optional).
- Administer infusion of Fabrazyme.
- Assess AEs.
- Record concomitant medications/therapies.

Patients are required to remain at the study site for approximately 1 hour after the infusion and until the Study Investigator determines the patient is stable. For those patients infused at home, the home infusion nurse must observe the patient for approximately 1 hour after completion of the infusion and until the home infusion nurse determines the patient is stable. All applicable regional regulations and procedures must be followed.

9.1.10 Week 104/Year 2

The following assessments/treatment will be completed at 2 years (Week 104/Year 2) for all patients (all assessments begin prior to the Fabrazyme infusion):

- Conduct a physical examination and measure height.
- Conduct vital signs assessments. Measurement of BP, HR, respiratory rate, and temperature will be performed before the infusion and 1 hour ±15 minutes after the completion of the infusion.
- Measure weight before the infusion. (***Note***: the weight measurement of the current visit will be used to calculate the infusion dose of Fabrazyme [in mg] to be administered at that visit. The weight at the preceding visit may be used if current weight is not available at the time of dose preparation.)
- Collect blood samples for clinical laboratory hematology and chemistry assessments.
- Collect plasma sample for plasma GL-3.
- Conduct injection and sample collection for GFR calculation via iohexol plasma clearance (must not be on the same day as the Fabrazyme infusion).
- Collect serum for r-hαGAL antibody titers.
- Collect blood and urine samples for biomarker assessment.
- Collect a urine sample for routine urinalysis.
- Collect 3 consecutive first voided morning urine samples, each at least 1 week apart, for total protein, albumin, RBP, β_2-microglobulin, and creatinine levels. (***Note:*** all 3 samples must be collected regardless of the results and collected prior to visit infusion.)
- Collect a urine sample for urine GL-3.
- Conduct an audiology examination.
- Perform an echocardiograph.
- Assess GI symptoms.
- Administer age-appropriate self-report instruments: PedsQL Pediatric or Young Adult Quality of Life Inventory, fatigue (PedsQL Multidimensional Fatigue Scale) and pain (PedsQL PPQ or BPI [SF] if 18 years or older).
- Perform an ECG.
- Pre-treat with an antipyretic/antihistamine (optional).
- Administer infusion of Fabrazyme.
- Assess AEs.

- Record concomitant medications/therapies.

Patients are required to remain at the study site for approximately 1 hour after the infusion and until the Study Investigator determines the patient is stable. For those patients infused at home, the home infusion nurse must observe the patient for approximately 1 hour after completion of the infusion and until the home infusion nurse determines the patient is stable. All applicable regional regulations and procedures must be followed.

9.1.11 Week 132/Year 2.5

The following assessments/treatment will be completed at 2.5 years for all patients (Week 132/Year 2.5) (all assessments begin prior to the Fabrazyme infusion):

- Conduct a physical examination and measure height.
- Conduct vital signs assessments. Measurement of BP, HR, respiratory rate, and temperature will be performed before the infusion and 1 hour ±15 minutes after the completion of the infusion.
- Measure weight before the infusion. (***Note***: the weight measurement of the current visit will be used to calculate the infusion dose of Fabrazyme [in mg] to be administered at that visit. The weight at the preceding visit may be used if current weight is not available at the time of dose preparation.)
- Collect a blood sample for serum creatinine.
- Collect plasma sample for plasma GL-3.
- Collect serum for r-hαGAL antibody titers.
- Collect blood and urine samples for biomarker assessment.
- Collect 3 consecutive first voided morning urine samples, each at least 1 week apart, for total protein, albumin, RBP, β_2-microglobulin, and creatinine levels. (***Note:*** all 3 samples must be collected regardless of the results and collected prior to visit infusion.)
- Collect a urine sample for urine GL-3.
- Perform a brain MRI (optional).
- Take retinal imaging photographs (optional).
- Take angiokeratoma photograph(s), if present.
- Assess GI symptoms.
- Administer age-appropriate self-report instruments: PedsQL Pediatric or Young Adult Quality of Life Inventory, fatigue (PedsQL Multidimensional Fatigue Scale) and pain (PedsQL PPQ or BPI [SF] if 18 years or older).
- Pre-treat with an antipyretic/antihistamine (optional).
- Administer infusion of Fabrazyme.
- Assess AEs.
- Record concomitant medications/therapies.

Patients are required to remain at the study site for approximately 1 hour after the infusion and until the Study Investigator determines the patient is stable. For those patients infused at home, the home infusion nurse must observe the patient for approximately 1 hour after completion of the infusion and until the home infusion nurse determines the patient is stable. All applicable regional regulations and procedures must be followed.

9.1.12 Week 156/Year 3

The following assessments/treatment will be completed at 3 years for all patients (Week 156/Year 3) (all assessments begin prior to the Fabrazyme infusion):

- Conduct a physical examination and measure height.
- Conduct vital signs assessments. Measurement of BP, HR, respiratory rate, and temperature will be performed before the infusion and 1 hour ±15 minutes after the completion of the infusion.
- Measure weight before the infusion. (***Note***: the weight measurement of the current visit will be used to calculate the infusion dose of Fabrazyme [in mg] to be administered at that visit. The weight at the preceding visit may be used if current weight is not available at the time of dose preparation.)
- Collect blood samples for clinical laboratory hematology and chemistry assessments.
- Collect plasma sample for plasma GL-3.
- Conduct injection and sample collection for GFR calculation via iohexol plasma clearance (must not be on the same day and must be prior to the Fabrazyme infusion).
- Collect serum for r-hαGAL antibody titers.
- Collect blood and urine samples for biomarker assessment.
- Collect a urine sample for routine urinalysis.
- Collect 3 consecutive first voided morning urine samples, each at least 1 week apart, for total protein, albumin, RBP, β_2-microglobulin, and creatinine levels. (***Note:*** all 3 samples must be collected regardless of the results and collected prior to visit infusion.)
- Collect a urine sample for urine GL-3.
- Conduct an audiology examination.
- Obtain a skin biopsy.
- Perform an echocardiograph.
- Assess GI symptoms.
- Administer age-appropriate self-report instruments: PedsQL Pediatric or Young Adult Quality of Life Inventory, fatigue (PedsQL Multidimensional Fatigue Scale) and pain (PedsQL PPQ or BPI [SF] if 18 years or older).
- Perform an ECG.
- Pre-treat with an antipyretic/antihistamine (optional).
- Administer infusion of Fabrazyme.

- Assess AEs.
- Record concomitant medications/therapies.

Patients are required to remain at the study site for approximately 1 hour after the infusion until the Study Investigator determines the patient is stable. For those patients infused at home, the home infusion nurse must observe the patient for approximately 1 hour after completion of the infusion and until the home infusion nurse determines the patient is stable. All applicable regional regulations and procedures must be followed.

9.1.13 Week 184/Year 3.5

The following assessments/treatment will be completed at 3.5 years (Week 184/Year 3.5) for all patients (all assessments begin prior to the Fabrazyme infusion):

- Conduct a physical examination and measure height.
- Conduct vital signs assessments. Measurement of BP, HR, respiratory rate, and temperature will be performed before the infusion and 1 hour ±15 minutes after the completion of the infusion.
- Measure weight before the infusion. (***Note***: the weight measurement of the current visit will be used to calculate the infusion dose of Fabrazyme [in mg] to be administered at that visit. The weight at the preceding visit may be used if current weight is not available at the time of dose preparation.)
- Collect a blood sample for serum creatinine.
- Collect plasma sample for plasma GL-3.
- Collect serum for r-hαGAL antibody titers.
- Collect blood and urine samples for biomarker assessment.
- Collect 3 consecutive first voided morning urine samples, each at least 1 week apart, for total protein, albumin, RBP, β_2-microglobulin, and creatinine levels. (***Note:*** all 3 samples must be collected regardless of the results and collected prior to visit infusion.)
- Collect a urine sample for urine GL-3.
- Assess GI symptoms.
- Administer age-appropriate self-report instruments: PedsQL Pediatric or Young Adult Quality of Life Inventory, fatigue (PedsQL Multidimensional Fatigue Scale) and pain (PedsQL PPQ or BPI [SF] if 18 years or older).
- Pre-treat with an antipyretic/antihistamine (optional).
- Administer infusion of Fabrazyme.
- Assess AEs.
- Record concomitant medications/therapies.

Patients are required to remain at the study site for approximately 1 hour after the infusion and until the Study Investigator determines the patient is stable. For those patients infused

at home, the home infusion nurse must observe the patient for approximately 1 hour after completion of the infusion and until the home infusion nurse determines the patient is stable. All applicable regional regulations and procedures must be followed.

9.1.14 Week 208/Year 4

The following assessments/treatment will be completed at 4 years (Week 208/Year 4) for all patients (all assessments begin prior to the Fabrazyme infusion):

- Conduct a physical examination and measure height.
- Conduct vital signs assessments. Measurement of BP, HR, respiratory rate, and temperature will be performed before the infusion and 1 hour ±15 minutes after the completion of the infusion.
- Measure weight before the infusion. (***Note***: the weight measurement of the current visit will be used to calculate the infusion dose of Fabrazyme [in mg] to be administered at that visit. The weight at the preceding visit may be used if current weight is not available at the time of dose preparation.)
- Collect blood samples for clinical laboratory hematology and chemistry assessments.
- Collect plasma sample for plasma GL-3.
- Conduct injection and sample collection for GFR calculation via iohexol plasma clearance (must not be on the same day as the Fabrazyme infusion).
- Collect serum for r-hαGAL antibody titers.
- Collect blood and urine samples for biomarker assessment.
- Collect a urine sample for routine urinalysis.
- Collect 3 consecutive first voided morning urine samples, each at least 1 week apart, for total protein, albumin, RBP, β_2-microglobulin, and creatinine levels. (***Note:*** all 3 samples must be collected regardless of the results and collected prior to visit infusion.)
- Collect a urine sample for urine GL-3.
- Conduct the audiology examination.
- Perform an echocardiograph.
- Assess GI symptoms.
- Administer age-appropriate self-report instruments: PedsQL Pediatric or Young Adult Quality of Life Inventory, fatigue (PedsQL Multidimensional Fatigue Scale) and pain (PedsQL PPQ or BPI [SF] if 18 years or older).
- Perform an ECG.
- Pre-treat with an antipyretic/antihistamine (optional).
- Administer infusion of Fabrazyme.
- Assess AEs.
- Record concomitant medications/therapies.

Patients are required to remain at the study site for approximately 1 hour after the infusion and until the Study Investigator determines the patient is stable. For those patients infused at home, the home infusion nurse must observe the patient for approximately 1 hour after completion of the infusion and until the home infusion nurse determines the patient is stable. All applicable regional regulations and procedures must be followed.

9.1.15 Week 236/Year 4.5

The following assessments/treatment will be completed at 4.5 years (Week 236/Year 4.5) for all patients (all assessments begin prior to the Fabrazyme infusion):

- Conduct a physical examination and measure height.
- Conduct vital signs assessments. Measurement of BP, HR, respiratory rate, and temperature will be performed before the infusion and 1 hour ±15 minutes after the completion of the infusion.
- Measure weight before the infusion. (***Note***: the weight measurement of the current visit will be used to calculate the infusion dose of Fabrazyme [in mg] to be administered at that visit. The weight at the preceding visit may be used if current weight is not available at the time of dose preparation.)
- Collect a blood sample for serum creatinine.
- Collect plasma sample for plasma GL-3.
- Collect serum for r-hαGAL antibody titers.
- Collect blood and urine samples for biomarker assessment.
- Collect 3 consecutive first voided morning urine samples, each at least 1 week apart, for total protein, albumin, RBP, β_2-microglobulin, and creatinine levels. (***Note:*** all 3 samples must be collected regardless of the results and collected prior to visit infusion.)
- Collect a urine sample for urine GL-3.
- Assess GI symptoms.
- Administer age-appropriate self-report instruments: PedsQL Pediatric or Young Adult Quality of Life Inventory, fatigue (PedsQL Multidimensional Fatigue Scale) and pain (PedsQL PPQ or BPI [SF] if 18 years or older).
- Pre-treat with an antipyretic/antihistamine (optional).
- Administer infusion of Fabrazyme.
- Assess AEs.
- Record concomitant medications/therapies.

Patients are required to remain at the study site for approximately 1 hour after the infusion and until the Study Investigator determines the patient is stable. For those patients infused at home, the home infusion nurse must observe the patient for approximately 1 hour after

completion of the infusion and until the home infusion nurse determines the patient is stable. All applicable regional regulations and procedures must be followed.

9.1.16 Week 260/Year 5 (Study Completion) or Early Withdrawal

The following assessments/treatment will be completed for all patients at 5 years or if they withdraw from the study prior to completion (Week 260/Year 5) (all assessments begin prior to the Fabrazyme infusion):

- Conduct a physical examination and measure height.
- Conduct vital signs assessments. Measurement of BP, HR, respiratory rate, and temperature will be performed before the infusion and 1 hour ±15 minutes after the completion of the infusion.
- Measure weight before the infusion. (***Note***: the weight measurement of the current visit will be used to calculate the infusion dose of Fabrazyme [in mg] to be administered at that visit. The weight at the preceding visit may be used if current weight is not available at the time of dose preparation.)
- Collect blood samples for clinical laboratory hematology and chemistry assessments.
- Collect plasma sample for plasma GL-3.
- Conduct injection and sample collection for GFR calculation via iohexol plasma clearance (must not be on the same day as the Fabrazyme infusion).
- Collect serum for r-hαGAL antibody titers.
- Collect blood and urine samples for biomarker assessment.
- Collect a urine sample for routine urinalysis.
- Collect 3 consecutive first voided morning urine samples, each at least 1 week apart, for total protein, albumin, RBP, β_2-microglobulin, and creatinine levels. (***Note:*** all 3 samples must be collected regardless of the results and collected prior to visit infusion.)
- Collect a urine sample for urine GL-3.
- Conduct the audiology examination.
- Obtain a skin biopsy.
- Obtain a kidney biopsy (optional) after randomization but prior to Day 1.
- Perform a brain MRI (optional).
- Perform an echocardiograph.
- Take retinal imaging photographs (optional).
- Take angiokeratoma photograph(s), if present.
- Assess GI symptoms.
- Administer age-appropriate self-report instruments: PedsQL Pediatric or Young Adult Quality of Life Inventory, fatigue (PedsQL Multidimensional Fatigue Scale) and pain (PedsQL PPQ or BPI [SF] if 18 years or older).
- Perform an ECG.

- Pre-treat with an antipyretic/antihistamine (optional).
- Administer infusion of Fabrazyme.
- Assess AEs.
- Record concomitant medications/therapies.

Patients are required to remain at the study site for approximately 1 hour after the infusion until the Study Investigator determines the patient is stable. For those patients infused at home, the home infusion nurse must observe the patient for approximately 1 hour after completion of the infusion and until the home infusion nurse determines the patient is stable. All applicable regional regulations and procedures must be followed.

9.1.17 Safety Follow-up

Study sites will attempt to reach all study participants by telephone approximately 28 days after the final study procedures are completed, for a final patient safety contact.

Patients will be encouraged to enroll in the Fabry Registry.

9.2 Efficacy Measurements

9.2.1 Evaluation of Skin Biopsies

For each study site, a physician experienced in skin biopsy technique and practice in children will be identified to ensure all tissue samples are correctly obtained and are appropriately processed for shipment. Sample processing, storage, and shipment guidelines are provided in the Study Operations Manual (SOM).

Evaluation of the GL-3 accumulation in superficial skin vascular endothelium (capillary), deep vessel endothelial cells, deep vessels smooth muscle cells, and perineurium cells will be conducted using light microscopy (LM).

Masked biopsy samples will be scored for GL-3 accumulation by a group of independent pathologists with expertise in the tissue examined. Scoring will be on a none-mild-moderate-severe scale (0-1-2-3). Each pathologist will be trained on the method of scoring by the Sponsor. Each pathologist will score each specimen. Analysis includes using each pathologist's score sheets to create a majority score calculated for that slide. As necessary, discrepant scoring of the sample will be resolved by an adjudication process using the original independent pathology reviewers. If a majority score cannot be derived from the adjudication process, then the median score will be used.

Skin biopsy results will be reported to the Study Investigator.

9.2.2 Evaluation of Kidney Biopsies

Optional kidney biopsies are recommended after randomization but prior to Day 1 and at Week 260/Year 5 or discontinuation or early withdrawal, if patient is stopping study

participation to start enzyme replacement therapy, in order to assess the extent of kidney damage and GL-3 accumulation before start of treatment. A pre-biopsy evaluation to rule out contraindication for a kidney biopsy should be performed and documented according to local hospital procedures. Kidney biopsies should not be performed on Day 1.

For each study site, a physician experienced in kidney biopsy technique and practice in children or adults, depending on the patient age, will be identified to ensure all tissue samples are correctly obtained and are appropriately processed for shipment. Sample processing, storage, and shipment guidelines are provided in the SOM.

Masked biopsy samples will be scored for GL-3 accumulation by a group of independent pathologists with expertise in kidney tissue. Scoring will be on a none-mild-moderate-severe scale (0-1-2-3). Each pathologist will be trained on the method of scoring by the Sponsor. Each pathologist will score each specimen. All scoring sheets will be forwarded to the Sponsor for compilation and analysis.

Histological evaluation of the GL-3 accumulation in capillary endothelium (vasculature) in kidney will be conducted using LM. Kidney biopsy samples will be processed for LM and examined for overall kidney architecture. Special attention will be paid to the degree (if any) of glomerulosclerosis as well as GL-3 accumulation in the different cell types.

Additional evaluations for the accumulation of GL-3 inclusions in the kidney samples will also be performed in other cell types, including glomerular endothelial cells, mesangial cells, podocytes, interstitial cells, non-capillary endothelial cells, non-capillary smooth muscle cells, distal convoluted tubules and collecting ducts cells using LM. These cells will be counted and scored by each pathologist assigning each cell an overall score of 0 to 2 or 0 to 3, as appropriate.

Additionally, duplicate tissue sections from kidney biopsies will be sent to a central laboratory for quantitative structural analysis by light and electron microscopy.

Kidney biopsy results will be reported to the Study Investigator.

9.2.3 GL-3 Evaluations

Clearance of GL-3 from plasma and urine will be evaluated by measuring levels of GL-3 using tandem Mass Spectrometry. Frozen plasma samples and urine samples for GL-3 analysis will be forwarded to central laboratories where the assays will be conducted. Refer to the SOM for details regarding sample collection and shipment.

9.2.4 Renal Function

Patients will be instructed to limit the intake of high protein foods, (e.g., meats, seafood, dairy products, eggs, peanuts, and legumes) and abstain from prohibited medications for

24 hours prior to blood or urine collection. Urine collection should be delayed if the patient has a fever.

9.2.4.1 Glomerular Filtration Rate

The GFR will be evaluated by 2 methods:

- GFR will be calculated by iohexol plasma clearance[29] (central laboratory assay). Samples will be taken at Baseline and 120, 150, 180, 210, 240 minutes (2, 2.5, 3, 3.5, and 4 hours) post iohexol injection. (See the SOM for procedure.) This procedure must not be performed on the same day as a Fabrazyme infusion or PK sampling.
  The rationale for and documentation of low protein intake is as follows. Diet can have an effect on GFR and in healthy subjects, GFR assessed by clearance of 51Cr-EDTA was found to be lower by 11.5 % in vegan subjects and by 8.8% in lactovegetarians than in omnivores in a comparative study.[30] In the same study, mean value for total protein intake was 0.95 g/kg/day for vegans (as compared to 1.29 g/kg/day for omnivores). Therefore, values for $GFR_{iohexol}$ ≥80 mL/min/1.73 m^2 will be acceptable for inclusion in this study, provided that protein intake ≤0.95 g/kg/day. Protein intake (g/day) will be calculated using the following formula[31]: 6.25 x [24-hour urine urea nitrogen (g/day) + (0.031 g N/kg/day) x desirable weight (kg)] + urine protein (g/day) in excess of 5 g/day. Only urine collections of 20- to 28-hours duration containing between 9 and 31 mg/kg/day of urinary creatinine will be accepted.
- Estimated GFR (eGFR) will be calculated from serum creatinine using an age-appropriate method (e.g., $eGFR_{Schwartz}$[32], $eGFR_{MDRD}$[33,34]).

9.2.4.2 Protein Excretion

Protein excretion will be evaluated from 3 consecutive first morning urine voids, each obtained at least 1 week apart. (***Note:*** all 3 samples must be collected regardless of the results and collected prior to visit infusion.) The following parameters will be measured/calculated:

- Protein/creatinine ratio
- Albumin/creatinine ratio
- RBP
- β_2-microglobulin
- Creatinine

Patients will be instructed to abstain from eating high protein foods (e.g., meats, seafood, dairy products, eggs, peanuts, and legumes) for 24 hours prior to collection of the first morning urine voids.

The central laboratory will not assay β2-microglobulin in acidic (pH<6.0) urine specimens, as β2-microglobulin is unstable in acidic urine. Therefore, all urine samples for measurement of β2-microglobulin should be pH-tested and normalized within 1 hour of collection, as described in the Quest Diagnostics Investigator Manual. To ensure that pH normalization occurs within 1 hour of sample collection, first morning urine voids should be obtained in the clinic whenever possible (and for at least 1 of the 3 samples obtained at each timepoint). If pH normalization of a sample cannot be performed within 1 hour of collection, this step will be omitted for that specific sample, because the central laboratory will be unable to ascertain when pH normalization occurred and will proceed with assaying the sample and results may not be representative.

All renal function analyses will be conducted by central laboratories. Procedures for handling and shipment of samples will be included in the information provided by each central laboratory. Specimens will be appropriately processed by the central laboratory facilities and laboratory reports will be made available to the Study Investigator in a timely manner to assure appropriate clinical review.

9.2.5 Ear Function

The presence or absence, type (sensory, conductive, or mixed) and degree of hearing loss (decibels) as compared to normal controls for age and gender will be assessed by a head, eyes, ears, nose, and throat (HEENT) specialist or equivalent as follows:

- Pure-tone audiograms with thresholds at 0.125, 0.25, 0.5, 1, 2, 4, and 8 kHz and bone conduction thresholds as appropriate.
- Otoscopy will be performed (mainly to exclude middle ear problems that could lead to hearing loss in children such as otitis media with or without effusion).

The patient and/or parent(s)/legal guardian(s) will be questioned regarding the presence or absence of tinnitus. If clinically significant (CS) hearing changes as compared to Screening results are noted, an AE will be recorded.

9.2.6 Brain Abnormalities

A brain MRI (without contrast agent) will be obtained in as many of the patients as possible, though the procedure remains optional. Parameters of interest include standard T2, T1, and FLAIR. Also, magnetic resonance angiography (MR angio) may be done to look at the larger vessels.

Two copies of the MRI recording will be generated for each requested timepoint. One copy will be kept at the study site with the interpretation of a site physician. A site physician will assess the MRI and record an overall conclusion as normal, abnormal but not CS, or

abnormal and CS. If the Study Investigator determines the MRI is abnormal and CS and a change from Screening, the result will be documented as an AE.

The second copy will be sent to the central laboratory for analysis. The central laboratory findings will be used for statistical analyses.

Specific procedures for these tests will be supplied to each Study Investigator. See the MRI manual for procedures for sending assessments to the central analysis laboratory.

Brain MRI results will be reported to the Study Investigator.

9.2.7 Echocardiograph

A 2-dimensional Doppler echocardiograph will be performed:

- To measure LVPWTd, IVSTd, LVDd (end diastolic left ventricular diameter),
- To calculate LVMI, mean wall (MW) thickness [(LVPWTd + IVSTd)/2] and ejection fraction (EF), and
- To evaluate valve abnormalities; LVMI, MW thickness [(LVPWTd + IVSTd)/2] and EF.

Optional tissue doppler imaging (TDI) parameters will also be collected (based on the study site's technical capabilities), as many as possible, to evaluate mitral annulus velocities with

- measured assessments of systolic (Sa), early diastolic (Ea), transmitral (E), and late diastolic (Aa) velocities, and
- calculated assessments of Ea/Aa and E/Ea ratios at both corners of the mitral annulus.

Two copies of the echocardiograph recording will be generated for each study timepoint. One copy will be kept at the study site with a site physician interpretation. A site physician will assess the echocardiograph in an overall conclusion as normal, abnormal but not CS, or abnormal and CS. Wherever possible, the same physician should review all echocardiographs for a given patient. If the Study Investigator determines the echocardiograph is abnormal and CS and a change from Screening, the result will be documented as an AE.

The second copy will be sent to the central laboratory for analysis. The central laboratory findings will be used for statistical analyses. Specific procedures for these tests are supplied to each Study Investigator in the Cardiocore Manual.

9.2.8 Retinal Imaging

Optional retinal digital imaging photographs (from a retinal photo of each eye) will be read by a central laboratory. Specific procedures are provided in the SOM.

9.2.9 Angiokeratoma

Angiokeratoma (if present) and any changes thereof will be fully described. Additionally, digital photograph(s) will be taken.

9.2.10 Gastrointestinal Symptoms

Gastrointestinal symptoms in all patients will be assessed and recorded. These include the following:

- Degree and frequency of abdominal pain,
- Degree and frequency of diarrhea,
- Degree and frequency of nausea,
- Degree and frequency of vomiting,
- Maximum number of bowel movements per day in the past week,
- Stool consistency, on average, in the past week,
- Severity of abdominal pain on average, in the past week, and
- Severity of bloating, on average, in the past week.

9.2.11 Quality of Life Questionnaires

Quality of life will be assessed using age-appropriate self-report instruments designed to measure the core dimensions of health (physical, emotional and social) and role (school/work) functioning, general, sleep/rest and cognitive fatigue and current/worst recent pain.

The following questionnaires will be administered to all patients at Day 1 and every 6 months thereafter or at early withdrawal:

- For core dimensions of health and role functioning: the PedsQL Pediatric or Young Adult Quality of Life Inventory,
- For general, sleep/rest and cognitive fatigue: the PedsQL Multidimensional Fatigue Scale, and
- For current and worst recent pain: the PedsQL PPQ or BPI (SF).

At each applicable visit, the appropriate questionnaire booklet will be selected based on the patient's age on the date the questionnaires are administered:

- Patients 5 through 7 years old will receive the Young Child (ages 5 - 7 years) booklet,
- Patients 8 through 12 years old will receive the Child (ages 8 - 12 years) booklet,
- Patients 13 through 17 years old will receive the Teen (ages 13 - 18 years) booklet. (***Note:*** Although the Teen Quality of Life and Fatigue Reports and Pain Form are validated for use through age 18 years, the Young Adult/Adult booklets will be used for all patients upon reaching 18 years of age.), or

- Patients 18 years of age or older will receive the Young Adult (ages 18-25 years)/Adult (age 18+ years) booklet.

Specific procedures for the administration of questionnaires are provided in the SOM.

9.2.12 Biomarkers

Biomarkers will be assessed from blood and urine samples collected prior to infusions. Biomarkers to be tested are not yet identified. (***Note***: No genetic testing will be performed on these samples.) Specific procedures for collecting and shipping samples are provided in the Quest Diagnostics Investigator Manual.

9.2.13 Criteria for Increasing the Study Drug Dose

Any of the following may warrant a dose increase to 1 mg/kg q2w of Fabrazyme, after consultation with the DMC and agreement of the Sponsor:

- Insufficient clearance (a score of 2 or more) of GL-3 from skin vascular endothelium (superficial) after Screening.
- Stroke or TIA or, if a brain MRI has been performed, the development of bright lesions >2 mm on T2- or FLAIR-weighted images within the white matter or the basal ganglia.
- Albuminuria (first morning void urinary albumin/creatinine ratio >30 mg/g on at least 2 out of 3 samples, each at least 1 week apart) (from the central laboratory).
- Echocardiograph findings (from the central laboratory):
  - Patients ≤18 years of age:
    - LVPWTd ≥2 mm above 2 SD more than normal (using BSA normal ranges[28]) and/or
    - IVSTd ≥2 mm above 2 SD more than normal (using BSA normal ranges).
  - Patients >18 years old:
    - LVMI >50 g/m2.7 and/or
    - Any parietal wall thickness >12 mm.
- Other parameters indicating Fabry disease is progressing and/or too advanced to keep the patient at low dose Fabrazyme.

9.3 Pharmacokinetic Profiles

Blood samples for PK profiles of Fabrazyme will be collected at Day 1 and at Week 52/Year 1 at 0 minutes (pre-dose), every hour after the start of the infusion, at the end of the infusion, at 15 minutes, at 30 minutes, at 1 hour, at 2 hours, at 4 hours, at 6 hours, and at 8 hours post-infusion. Samples scheduled from 0 minutes (pre-dose) through 2 hours post-infusion must be collected ≤5 minutes from the nominal timepoint, and samples scheduled from 4 to 8 hours post-infusion must be collected ≤10 minutes from the nominal timepoint. An IV catheter will be placed in a vein of the forearm, contra-lateral to the

infusion site, to collect blood samples. Patients who are receiving infusions at home will return to the site for the Week 52/Year 1 infusion and PK profile.

Blood samples will be collected from patients at all study sites that have the capability of collecting and processing samples for PK analyses, in accordance with local regulations.

A sample of blood is drawn at each of the PK timepoints into heparinized tubes for a total of 25 mL of blood for each of the 2 PK profiles. The total amount of blood should not exceed 1 mL/kg in compliance with the European Medicines Agency (EMA) note of guidance.[35] In the instance this would be exceeded, the Study Investigator will contact the Sponsor in order to discuss the deletion of some sampling timepoints. Each blood sample will be centrifuged and the plasma removed. Aliquots of the plasma preparation will be frozen at -20 to -70°C and shipped for analysis. More detail for procedures for sample collection, handling, and shipment are provided in the SOM.

9.4 Safety Measurements

Safety will be monitored by GPS-RM for reported AEs, IARs, and SAEs. A DMC will also periodically review changes in physical examination, vital signs, clinical laboratory evaluations, and ECGs; and antibody formation to r-hαGAL. A description of DMC safety review is described in Section 9.6.

9.4.1 Physical Examination

Whenever possible, the same physician should perform the physical examination at all study visits. The findings of each examination will be recorded. Each physical examination will include the following physical observations/measurements:

- General Appearance
- Skin
- HEENT
- Lymph Nodes
- Heart
- Lungs
- Extremities/Joints
- Abdomen
- Neurological
- Mental Status
- Measurement of height
- External Genitalia (including Tanner stage, see Appendix 14.3)[36]

If CS worsening from the Screening assessment is noted on physical examination, the changes will be documented as AEs. Clinical significance is defined as any variation in physical findings from the patient's pre-treatment findings that has medical relevance. The Study Investigator will continue to monitor the patient until the parameter returns to screening assessment levels or until the Study Investigator determines that follow-up is no longer medically necessary.

9.4.2 Vital Signs and Weight

The following measurements will be obtained and recorded at all visits:

- Systolic and diastolic BP
- HR
- Respiratory rate
- Temperature
- Weight

Three resting BP measurements will be taken for each measurement (the child is to sit and relax, 3 measurements are taken one after the other). The third BP measurement will be recorded on the eCRF. Vital signs will be measured both before and 1 hour ±15 minutes after completion of each infusion.

Weight will be measured before each infusion. (***Note***: the weight measurement of the current visit will be used to calculate the infusion dose of Fabrazyme [in mg] to be administered at that visit. The weight at the preceding visit may be used if current weight is not available at the time of dose preparation.)

If, in the Study Investigator's opinion, CS vital sign changes (compared to the Screening assessment) occur, the changes will be documented as AEs. Clinical significance is defined as any variation in vital signs from the patient's pre-treatment findings, which has medical relevance. The Study Investigator will continue to monitor the patient until the parameter returns to Screening assessment levels or until the Study Investigator determines that follow-up is no longer medically necessary.

9.4.3 Clinical Laboratory Evaluations

9.4.3.1 Clinical Laboratory Assessments

The following serum hematology, chemistry, and urinalysis assessment samples will be obtained <u>prior</u> to administration of the scheduled infusion:

- Complete blood count (CBC including, but not limited, to red blood cell [RBC] count, white blood cell [WBC] count, platelet count, hemoglobin [Hgb], and Hematocrit [Hct]) with differential.
- Blood urea nitrogen (BUN), glucose, uric acid, calcium, phosphorus, magnesium, albumin, total protein, creatinine, electrolyte panel (including but not limited to sodium, potassium, and chloride), serum glutamic oxaloacetic transaminase (SGOT), serum glutamic pyruvic transaminase (SGPT), total bilirubin, alkaline phosphatase, and C-reactive protein.
- Routine urinalysis (including but not limited to urine microscopy, metered pH, specific gravity), creatinine, total protein, and albumin.

Analysis of all safety laboratory parameters will be conducted by a central laboratory. Procedures for handling and shipment of all central laboratory samples will be included in

the information provided by the facility. Specimens will be appropriately processed by the central laboratory facility and laboratory reports will be made available to the Study Investigator in a timely manner to assure appropriate clinical review.

The Study Investigator must score all abnormal laboratory values as either CS or not clinically significant (NCS). It is anticipated that some laboratory values may be outside of the normal value range due to the underlying disease. As in routine practice, the Study Investigators should use clinical judgment when considering clinical significance. Clinical significance is defined as any change in laboratory parameters from Screening, which has medical relevance. If CS worsening of laboratory values from Screening levels is noted, the changes will be documented as an AE. The Study Investigator will continue to monitor the patient with additional laboratory assessments until (1) values have reached normal range and/or Screening levels, or, (2) in the judgment of the Study Investigator, abnormal values are not related to the administration of study medication or other Protocol-specific procedures.

9.4.3.2 IgG Antibody Testing

Blood samples for evaluation of IgG antibody to r-hαGAL must be obtained prior to study drug infusion on the collection schedule outlined in Table 9-1 and Table 9-2.

Additional testing for IgG antibodies may be performed for patients who experience a moderate to severe IAR or a recurrent IAR of any intensity. If the IAR occurs at an infusion when a pre-infusion IgG sample was not collected, the patient will be asked to return to the site after the reaction, at least 3 days post infusion or prior to the next infusion, for serum sample collection for IgG antibody testing.

See the SOM for guidelines on collection of serum samples for r-hαGAL antibody assessment. These blood samples will be analyzed by a central laboratory. Detailed procedures for handling and shipment of these samples are provided in the SOM.

9.4.3.3 IgE Antibody, Serum Tryptase and Complement Activation Testing

Patients with moderate or severe IARs may be tested for serum tryptase levels and plasma complement activation. Serum and plasma samples (for tryptase and complement, respectively) should be drawn within 1 to 2 hours of the onset of the event. If a patient experiences a moderate or severe IAR during a home infusion, the home infusion nurse should draw the serum and plasma samples within 1 to 2 hours of the onset of the reaction for complement activation and tryptase testing, whenever possible and where permitted by local regulations.

Should a patient experience a moderate to severe or recurrent IAR suspected to be IgE-mediated, GPS-RM Department will be contacted to confirm the need for

product-specific IgE testing. If confirmed and a pre-infusion serum sample was collected, it will be tested for IgE antibodies. If a pre-infusion sample was not collected, the patient will be asked to return to the site after the reaction, either at least 3 days post infusion or prior to the next infusion, for serum sample collection for IgE antibody testing.

These serum and blood samples will be shipped to a central laboratory, where they will be analyzed. Procedures for collection and shipment of these samples are detailed in the SOM. Reports for IgE antibodies to r-hαGAL will be generated and forwarded to GPS-RM Department. The Study Investigator will be notified of the IgE antibody results or other findings which may call into question the patient's ability to safely receive Fabrazyme and continue in the study.

9.4.3.4 Skin Testing

If a patient experiences a moderate to severe IAR or recurrent IARs and has no serological evidence of IgE antibodies, then the patient should be skin tested. Detailed instructions for the skin test procedure are provided in the SOM. Fabrazyme 5 mg for skin testing will be supplied by the Sponsor.

9.4.3.5 Immune Complex and Inhibitory Antibody Testing

For patients with symptoms (including but not limited to nephrosis, nephritis, or arthritis) of concern for immune complex disease, the patients' plasma samples may be obtained and evaluated for circulating immune complexes (CIC). For patients in whom lack of Fabrazyme efficacy is considered, inhibitory antibody testing may be performed. Global Patient Safety and Risk Management will be contacted for possible CIC and inhibitory antibody testing.

9.4.4 Electrocardiogram

A standard 12-lead ECG will be performed. All ECG recordings will be performed prior to phlebotomy and infusion. Two original ECG tracings will be generated for each requested timepoint. One tracing will be kept at the study site with a site physician interpretation. A site physician will assess the ECG and determine an overall conclusion as normal, abnormal but not CS, or abnormal and CS. If the Study Investigator determines the ECG is abnormal and CS and a change from Screening, the result will be documented as an AE.

The following ECG data will be generated from the central laboratory findings: RR interval, PR interval, QT interval, QTc (Bazett and Fredericia formulae) interval, QRS duration, and HR. Furthermore, rhythm, conduction, morphology, ST segment, T waves, and U waves will be assessed.

The second original tracing will be sent to the central laboratory for analysis. The central laboratory findings will be used for statistical analyses. Specific procedures for these tests are supplied to each Study Investigator in the Cardiocore Manual.

9.4.5 Adverse Events

The Investigator is responsible for monitoring the safety of patients who have enrolled in the study and for accurately documenting and reporting information as described in this section.

Adverse event assessments will be conducted at each visit. As part of the assessment, the patient and/or the patient's parent(s)/legal guardian(s) will be queried with the following question: "Since your last questioning or visit, has the patient (or have you) experienced any health problems?"

An AE is any undesirable physical, psychological, or behavioral effect experienced by a patient during their participation in an investigational study, in conjunction with the use of the drug or biologic, whether or not product-related. This includes any untoward signs or symptoms experienced by the patient from the time of signing of the informed assent/consent until completion of the study.

Adverse events may include, but are not limited to the following:

- Subjective or objective symptoms spontaneously offered by the patient and/or observed by the Study Investigator or medical staff.
- Findings at physical examinations.
- Laboratory abnormalities of clinical significance.

Disease signs, symptoms, and/or laboratory abnormalities already existing prior to the use of the product are not considered AEs after treatment unless they reoccur after the patient has recovered from the pre-existing condition or, in the opinion of the Study Investigator, they represent a CS exacerbation in intensity or frequency.

Any signs and symptoms experienced by the patient from the time of signing of the informed assent/consent through approximately 28 days post last dose of study medication will be recorded.

If CS worsening from pre-treatment is noted, the changes will be documented as AEs. Clinical significance is defined as any variation from the patient's pre-treatment that has medical relevance. The Study Investigator will continue to monitor the patient until the parameter returns to pre-treatment or until the Study Investigator determines that follow-up is no longer medically necessary.

The Study Investigator will assess the relationship of all AEs as being unrelated, unlikely/remote, possible, probable, or definitely related to the study medication. The AEs

classified by the Study Investigator as definite, probable, or possibly related are considered drug-related. Those classified as unlikely/remote related or unrelated are considered drug-unrelated.

9.4.5.1 Infusion-Associated Reactions

An IAR is defined as an AE that occurs on the day of the infusion (during the infusion or following completion of the infusion) and is considered by the Study Investigator to be related to Fabrazyme.

Notification procedures and guidelines for the management of IARs are provided in Appendix 14.1. Patients experiencing an IAR will be managed based on standard medical care practices and according to the guidelines in Appendix 14.2. Patients experiencing a moderate to severe IAR may be tested for serum IgE antibodies, serum tryptase, plasma complement activation, and/or skin test positivity based on guidelines in Appendix 14.2.5. Patients may be pre-treated according to the IAR management guidelines summarized in Appendix 14.2.4 before subsequent dosing of study medication. Patients experiencing an IAR during a home infusion will return to the study site. Details of the procedures for patients experiencing IARs during home infusion are located in Section 7.3.

A consultant allergist, independent from the DMC, will be available to confer with GPS-RM regarding IARs and skin testing results on an as needed basis. These recommendations and processes will be included for DMC review.

9.4.5.2 Serious Adverse Events

Serious adverse events will be captured from signed consent through approximately 28 days post last dose of study medication. For patients who discontinue prematurely from the study, SAEs will be reported from signed consent through approximately 28 days after last study contact. An SAE is defined as any AE that results in any of the following outcomes:

- Death.
- Life-threatening experience.
- Required or prolonged inpatient hospitalization.
- Persistent or significant disability/incapacity.
- Congenital anomaly.
- Important medical events.

Life-threatening experience: Any AE that places the patient, in the view of the reporter, at immediate risk of death from the AE as it occurred, i.e., does not include an AE that might have caused death had it occurred in a more severe form.

Persistent or significant disability/incapacity: The AE that resulted in a substantial disruption of a person's ability to conduct normal life functions.

Important medical events: The AEs that may not result in death, be life-threatening, or require hospitalization may be considered an SAE when, based upon appropriate medical judgment, may jeopardize the patient and may require medical or surgical intervention to prevent 1 of the outcomes listed above.

The Study Investigator will be asked to assess the severity of the adverse drug/biologic experience using the following categories: Mild, Moderate, and Severe. This assessment is subjective and the Study Investigator should use medical judgment to compare the reported AE to similar type events observed in clinical practice. Below are listed guidelines for severity assessment:

- Mild: Symptom(s) barely noticeable to the patient or does not make the patient uncomfortable. The AE does not influence performance or functioning. Prescription drugs are not ordinarily needed for relief of symptom(s).
- Moderate: Symptom(s) of a sufficient severity to make the patient uncomfortable. Performance of daily activities is influenced. Treatment of symptom(s) may be needed.
- Severe: Symptom(s) of a sufficient severity to cause the patient severe discomfort. Severity may cause cessation of treatment with the study drug. Treatment for symptom(s) may be given.

Severity is not equivalent to seriousness.

9.4.5.3 Adverse Event, Infusion-Associated Reaction, and Serious Adverse Event Reporting

The necessity and time requirements for reporting of AEs to the Sponsor or designee are as follows:

- All SAEs and disease progression requiring dose increase will be documented and reported within 24 hours of the Study Investigator's first knowledge of the event, even if the experience does not appear to be related to the study drug. All IARs will be documented and reported within 24 hours of the Study Investigator's first knowledge of the event. The Sponsor's GPS&RM Fax is available for SAE reporting on a 24-hour basis and is reviewed during normal business hours:

<table>
<tr><th colspan="2">Genzyme Global Patient Safety and
Risk Management</th></tr>
<tr><td colspan="2">Fax: +1 (617) 761-8506 (Global Triage)
E-mail: pharmacovigilancesafety@genzyme.com</td></tr>
<tr><td>United States & Non-European Countries
Phone: +1 (617) 768-9000 option 2
or +1 (800) 745-4447</td><td>Europe
Phone: +31(0) 35 699 1299</td></tr>
</table>

- For all SAEs, a detailed written description that includes copies of relevant patient records, autopsy reports, and other documents will be sent to GPS-RM.
- For all IARs, a written description with relevant information that includes infusion volume, dose (mg), infusion rate at the time of the event, and infusion rate adjustments and corrective medication will be sent to GPS-RM.
- If a patient has an SAE or IAR in the homecare setting, the home infusion will immediately report the details of the SAE or IAR to the Study Investigator (or his/her designee), regardless of the SAE or IAR severity. The Study Investigator is responsible for notifying GPS-RM.
- The Study Investigator must follow patients with adverse experiences until their condition resolves or stabilizes. Certain conditions that are not expected to resolve, such as metastatic cancer, need not be followed indefinitely by the Study Investigator.

The Sponsor is responsible for notifying Study Investigators of any expedited SAEs (AEs that are serious, unexpected, and related to study drug). The Sponsor will report all expedited SAEs to the appropriate regulatory agencies by the required timelines.

It is the responsibility of the non-EU Study Investigator to notify their Institutional Review Board (IRB) or Research Ethics Board (REB) and the responsibility of GPS-RM to notify the EU Independent Ethics Committees (IECs) of SAEs according to local requirements.

All AEs will be recorded, with a full description including the nature, date of onset and resolution, determination of seriousness, severity, action taken, outcome, and relationship to study drug.

9.4.6 Concomitant Medications

A concomitant medication is any medication, prescription or over-the-counter, taken by a patient during their participation in an investigational study. All medications taken by the patient from the time of signing the assent/consent through to study completion will be recorded. In addition, at every visit, the site personnel will record any medication used by the patient since a previous visit.

9.4.6.1 Medications Affecting Creatinine

Patients will be instructed to withdraw and abstain from medications and foods that are known to increase/decrease serum creatinine levels and/or urinary creatinine secretion for 24 hours prior to creatinine testing. The following medications are known to increase/decrease serum creatinine levels:

- Acetohexamide
- Ascorbic acid
- H-2 blockers
- Acute diuretics

- Cephalosporins
- Flucytosine
- Sulfonamides
- Trimethoprim

Patients will also be instructed to abstain from eating high protein foods (e.g., meats, seafood, dairy products, eggs, peanuts, and legumes) for 24 hours prior to creatinine testing.

9.4.6.2 Angiotensin Converting Enzyme Inhibitors/Angiotensin Receptor Blockers Therapy

Patient treatment with ACEI/ARB medication is an enrollment exclusion criterion. If, during the study, it becomes medically appropriate to institute ACEI/ARB therapy, the Medical Monitor must be notified in advance, as these drugs may affect the evaluation of albumin excretion rate.

9.4.7 Concomitant Therapies

A concomitant therapy is any medical procedure or non-medicinal treatment (e.g., cold soaks) received by the patient for the treatment of Fabry disease, and which could influence the assessment of the efficacy of Fabrazyme.

All concomitant therapies will be recorded from the time of signing the assent/consent through to study completion. In addition, at every visit, the site personnel will record any concomitant therapy received by the patient since a previous visit.

9.4.8 Pregnancy

If a sexual partner of a patient becomes pregnant any time after patient randomization, the progress of the pregnancy must be followed until the outcome of the pregnancy is known (i.e., delivery, elective termination, or spontaneous abortion). If the pregnancy results in the birth of a child, additional follow-up information may be requested. The Study Investigator must notify the Sponsor within 24 hours of first learning of the occurrence of pregnancy using the all appropriate Pregnancy Notification Forms (PNF) and faxing to the Sponsor providing as much information as possible. Since study patient is male, the Study Investigator must complete and provide both PNF A and B. When the Study Investigator becomes aware of the pregnancy outcome this information must also be reported to the Sponsor within 24 hours using the Pregnancy Outcome Form (POF) C. For reporting additional information about the pregnancy use PNF A and/or B and indicate "follow-up" on the form. Note that pregnancy in and of itself is not an AE or an SAE.

9.4.9 Safety Follow-Up

Study sites will attempt to reach all study participants by telephone approximately 28 days after the final study procedures are completed, to conduct concomitant medication/therapy and adverse experience/event assessment.

9.5 Other Assessments

9.5.1 Alpha-Galactosidase A

Leukocyte and plasma samples for αGAL activity will be collected to verify diagnosis of Fabry disease. Leukocyte αGAL activity level testing will be considered definitive. If the leukocyte αGAL activity assay is difficult to obtain, the patient may be enrolled based on documented plasma αGAL <1.5 nmol/hr/mL, with the agreement of the Medical Monitor. Blood collection, processing, and shipping instructions are provided in the SOM.

9.5.2 Genotyping

If the nucleotide (and amino-acid, if relevant) sequence of the αGAL mutation is unavailable, a blood sample will be collected for determination of the genotype. Blood collection, processing, and shipping instructions are provided in the SOM.

No other genetic testing will be conducted.

9.6 Data Monitoring Committee

An independent DMC appointed by the Sponsor will periodically monitor safety and other confidential data from this study. This committee will be comprised of experts in relevant biomedical fields who have no direct relationship with the study. Outcome data will be privileged and shared only with the DMC members during conduct of the study.

The purpose of the DMC in this clinical study is to make recommendations based on ongoing safety data, including AEs, and for possible dose increase for a specific patient's disease progression. All expedited (serious, unexpected, related) SAEs will be sent to the DMC for review as they occur. All other AEs will be compiled and sent to the DMC prior to a scheduled meeting date. The DMC will make its recommendations by periodically monitoring data, outcomes, toxicity, safety, and other confidential data. Any interim analyses results will be submitted to the DMC. Data concerning drug discontinuation for safety reasons or for potential dose increase due to disease progression will be reviewed by the DMC on an ad hoc basis.

It is expected that the DMC will meet to review the study data biannually and as needed.

10. DATA COLLECTION, QUALITY ASSURANCE, AND MANAGEMENT

10.1 Recording of Data

Clinical data will be captured using electronic data capture (EDC) technology unless otherwise specified in this document. Required data for this study are to be recorded in the patient's medical notes/source documents first and then entered into the eCRF by authorized site personnel. Any changes made to the data after initial entry into the eCRF will be captured via an electronic audit trail. Clinical data that are not recorded on the eCRF will be captured by electronic means and transferred to the Sponsor. The Study Investigator must provide direct access to the source documents to the Sponsor or its designee.

Home infusion nurses will work with the site personnel to ensure data is transferred and transcribed to the Study Investigator site in an accurate and timely manner based on procedures described in the country- or site-specific home infusion manual. All eCRF data entry related to home infusions will be performed by appropriately trained staff at the study site.

10.2 Data Quality Assurance

The eCRFs will be reviewed by a clinical monitor from the Sponsor or its designee for completeness and accuracy. Source document verification will be performed. The data will also be reviewed internally by data management or its designee and if necessary, the investigational sites will be queried for corrections and/or clarifications. Upon completion of data entry and cleaning, all user access privileges to the EDC system will be changed to read-only.

For each patient an electronic representation of the eCRFs, including all queries and the audit trail, will be created and sent to the sites upon completion of a quality assurance check by the Sponsor or its designee. Copies of pertinent records in connection with the study, including patient charts, laboratory data, etc, will also be maintained at the study site as part of source documentation (see Record Retention, Section 12.3.3).

10.3 Data Management

The format and content of the eCRF will be approved by the Sponsor or its designee prior to the start of the study. The Sponsor or its designee will be responsible for EDC database creation and management of data from sources other than the EDC database (e.g., non-safety specialty laboratory data).

Prior to finalizing and locking the database, all decisions concerning the inclusion or exclusion of patient data for analysis will be determined by appropriate clinical and statistical personnel. Any exclusions will be documented. Protocol deviations will be tracked by the Sponsor or its designee.

11. STATISTICAL METHODS AND PLANNED ANALYSES

A detailed statistical analysis plan will be developed prior to database lock.

11.1 Study Populations

The statistical analysis will be conducted on the full analysis set (FAS) and per-protocol (PP) Populations. These populations are defined as follows:

- FAS Population: All randomized patients who receive at least 1 infusion of study treatment.
- PP Population: All patients from the FAS Population who are at least 80% compliant with study drug dosing and who do not have any other major protocol violation.
- Safety Population: Safety analyses will be conducted for all randomized patients receiving at least 1 infusion of Fabrazyme.

11.2 Power and Sample Size

The determination to enroll approximately 35 male patients was chosen on medical grounds as a compromise between the need to assess safety, efficacy, and PK in the largest population and the limitation for recruitment of this pediatric population in the context of this orphan disease. No formal statistical sample size calculation has been performed.

11.3 Missing or Invalid Data

Missing data will not be imputed.

11.4 Demographics and Baseline Characteristics

Demographic and Baseline (last pre-treatment value) characteristics including gender, ethnicity, age of study treatment initiation, age of initial symptoms, age of diagnosis, genotype, and αGAL activity level will be summarized descriptively. For categorical variables, frequencies and percentages will be presented. For continuous variables, descriptive statistics (N, mean, median, SD, and range) will be presented. Prior and concomitant medications will be summarized descriptively.

11.5 Patient Accountability

All patients enrolled in the study will be included in the summary of patient disposition and accountability. A listing of patients indicating enrollment into the study, discontinuation from the study, and completion of the study will be generated.

11.6 Study Drug Treatment Usage and Compliance

The patient's compliance with the treatment regimen will be monitored in terms of the patient receiving the study drug infusion within the time period allowed in the protocol, missed or incomplete infusions, and dose received. Missed or incomplete infusions will be

clearly documented and considered in the analysis plan. Under certain circumstances, study drug dose may be increased to the approved dose of 1 mg/kg q2w. These criteria are described in detail in Section 9.2.13 of this protocol and will be documented and considered in the analysis plan.

11.7 Objectives and Endpoints

The objectives of this open-label study are to evaluate the efficacy (GL-3 clearance), PK, and safety parameters (including immunogenicity) for 2 alternative dose regimens of Fabrazyme (0.5 mg/kg q2w and 1.0 mg/kg q4w). The endpoints to evaluate these objectives and the statistical methodology to be used to analyze these endpoints are described below.

Unless stated otherwise, the results of continuous efficacy variables will be summarized by treatment group using summary statistics (N, mean, SD, median, range). Additionally, for change from Baseline (last observation prior to treatment) analyses, the change from Baseline for all timepoints and by treatment group will be calculated and presented. For categorical variables, frequencies and percentages will be presented by treatment group. Changes from Baseline will be summarized as appropriate by treatment group. Graphs may be presented as appropriate.

11.7.1 Efficacy Endpoints

Primary, secondary, and exploratory efficacy analyses are described below. The evaluations will be performed on the schedule described in Section 9.1 of this protocol.

11.7.1.1 Primary Efficacy Endpoint

The primary efficacy endpoint will be histological evaluation of GL-3 inclusions in the superficial skin vascular endothelium conducted using LM.

Scoring of GL-3 inclusions will reported as 0, 1, 2 or 3 where 0 = none (normal), and non-zero (1 [mild], 2 [moderate] or 3 [severe]) = abnormal. Scores will be categorized as normal (score = 0) or abnormal (score = 1, 2 or 3).

Summary statistics (N, %) for skin GL-3 at final visit will be presented by category (zero, non-zero) for each treatment group. A binomial matched pair procedure will be used to compute a p-value for each treatment group based on shifts from Baseline to the timepoint for a zero-nonzero score.

Shift tables showing skin GL-3 scores at Baseline and at each timepoint will be produced. A listing of skin GL-3 scores by patient and timepoint will be produced which summarize individual patient changes.

Additional evaluations of deep vessel endothelium, arteriolar smooth muscle cells, and perineurium may be conducted if present in the biopsy sample. These additional evaluations will be summarized by timepoint and treatment group and will be listed.

11.7.1.2 Secondary Efficacy Endpoints

The secondary efficacy endpoint is the effect of Fabrazyme treatment on GL-3 clearance in plasma and urine. These endpoints will be reported by a central laboratory. Change from Baseline will be presented and the percent of patients with normal/abnormal plasma or urine GL-3 at each timepoint will be presented by treatment group. Graphs will be prepared showing the mean and the median plasma or urine GL-3 value over time. The 95% confidence intervals (CIs) will be calculated and included on the graph. Similar graphs will be prepared for urine GL-3 data.

11.7.1.3 Exploratory Efficacy Endpoints

The following exploratory efficacy endpoints will be summarized using summary statistics (N, % for categorical values; N, mean, SD, median, range for continuous variables) by treatment group for each timepoint. Where appropriate, change from Baseline to on-study visit assessments will be prepared. Graphs will be prepared where appropriate.

1. Renal Function: GFR will be calculated by iohexol plasma clearance. Estimated GFR will be calculated using an age-appropriate algorithm (e.g., $eGFR_{Schwartz}$ or $eGFR_{MDRD}$). Urine total protein/creatinine and albumin/creatinine ratios and RBP and β_2-microglobulin levels will be calculated. All renal function parameters will be summarized by timepoint and change from Baseline.
2. Audiology: Standard hearing examinations and audiograms will be summarized by timepoint and change from Baseline.
3. BMI: BMI will be calculated from weight and height values collected at Screening, every 3 months through Week 52/Year 1, and every 6 months thereafter or at early withdrawal.
4. MRI: MRI and MR angio results will be summarized by timepoint by treatment group using summary statistics.
5. Echocardiography: Echocardiography results (all parameters) will be summarized by timepoint by treatment group.
6. Retinal Imaging: Results will be summarized by timepoint by treatment group.
7. Angiokeratoma: Changes noted in angiokeratoma (if present) will be summarized by timepoint by treatment group.
8. GI Symptoms: GI symptoms will be summarized.
9. Quality of Life: All quality of life data will be summarized separately by questionnaire

and timepoint see Section 9.2.11 for details.

10. Kidney biopsy: Optional kidney biopsy data will be summarized.
11. Biomarkers: Results from blood and urine data collected for biomarkers will be summarized.
12. IgG AUC/titers: Efficacy data may be summarized by IgG area under the curve (AUC)/titers.
13. Comparison: Natural history patient cohorts may be used as comparator groups, as appropriate.

11.7.2 Pharmacokinetic Endpoints

Blood samples for PK profiles of Fabrazyme will be collected at 2 timepoints (Day 1 and at Week 52/Year 1). Samples will be collected at pre-dose, during infusion, end of the infusion, and post-infusion timepoints. The PK sampling details are described in Section 9.3 of this protocol.

The PK concentration data will be summarized by treatment group using descriptive statistics at each visit and timepoint. All concentrations less than the limit of quantification will be set to missing. Fabrazyme PK will be characterized using compartmental methods within the framework of a nonlinear mixed effects model using NONMEM (version 6 or higher, Globomax ICON LLC). Standard model building techniques will be used. First, a structural model will be used to determine the appropriate compartmental structure of the model. Model selection will be based on overall goodness of fit, residual plots, precision of the parameter estimates, and the likelihood ratio test (LRT) using a p-value of 0.05 for significance in the case of nested models or on the Akaike Information Criteria in the case of non-nested models. However, because of the pediatric nature of this study, weight will be built into the model as a covariate a priori in accordance with allometric principles. Further, the allometric exponents for clearance terms will be fixed to 0.7 and for volume terms fixed to 1.0 at the outset. Inter-occasion variability will be tested as part of the structural model development. Once the appropriate compartmental model is identified, the following covariates will be examined for further improvement in the goodness of fit: age, sex, and antibody titer. Covariates will be included in the model if the p-value for the LRT is less than or equal to 0.01. Covariates will be tested using a stepwise regression approach. Once the final model is identified, model validation will consist of bootstrap testing and predictive checks. Because of the limited size of the study, data splitting will not be done for model development. When the final model has been validated, each patient's PK parameters will be estimated using maximum a posteriori estimation using the POSTHOC option in NONMEM. From the primary PK parameters, the following derived parameters will be calculated: area under the plasma concentration-time curve extrapolated to infinity

($AUC_{0-\infty}$), maximum plasma concentration (C_{max}), time to maximum plasma concentration (T_{max}), steady-state volume of distribution (Vss), alpha-half-life, and beta-half-life.

Plasma concentrations and PK parameters will be summarized by infusion (Day 1 and Week 52/Year 1) using descriptive statistics and graphical displays. The mean plasma concentration-time plots by infusion will be presented in a graph. Friedman's test will be used to test for differences between visits using a significance level of 0.05. This test will be done on clearance (CL),Vss, C_{max}, $AUC_{0\text{-}inf}$, and beta-half-life to examine patient differences between visits.

11.7.3 Safety Endpoints

The schedule for the collection of all safety endpoints can be found in Section 9.1 of this protocol. Safety parameter results will be provided to GPS-RM and the DMC on a regular basis. The following safety parameters will be evaluated:

- Physical examination.
- Vital signs (BP, HR, respiratory rate, body temperature).
- Laboratory parameters.
- ECG measurements.
- Antibody development to r-hαGAL (***Note***: that additional testing (IgE, skin testing, complement activation, serum tryptase, inhibitory antibody, immune complex) may be performed when/if appropriate.)
- AEs (including IARs and disease progression requiring dose increase).
- Concomitant medication and therapies.

All randomized patients who receive at least 1 infusion of Fabrazyme will be included in the safety population. Safety variables will be summarized by treatment group. For all safety variables that are continuous, summary statistics (N, mean, median, SD, range) will be presented along with their change from pre-treatment to final study visit. For categorical variables, the N and percent will be displayed at appropriate timepoints.

11.7.3.1 Vital Signs and Physical Examinations

Vital signs and physical examination results will be summarized for appropriate timepoints. Additionally, the change from Baseline to final study visit will be summarized.

11.7.3.2 Clinical Laboratory Parameters

Clinical laboratory parameters (hematology, chemistry, and urinalysis) will be summarized by timepoint by treatment group using descriptive statistics. Change from Baseline and shifts (normal, low, high) will be calculated and displayed by treatment group. Listings of CS abnormal laboratory values will be provided. Graphical displays will be prepared and presented as appropriate.

11.7.3.3 Electrocardiogram Measurements

Study Investigator-determined ECG overall results (normal, abnormal/NCS, abnormal/CS) will be summarized. Centrally-read ECG results will be summarized by treatment group. Listings of patients with CS abnormal ECG findings will be prepared. Graphical displays may be presented as appropriate.

11.7.3.4 Antibody Development to Fabrazyme

Antibody development (IgG) to Fabrazyme will be summarized descriptively by treatment group for each timepoint. Additional related data (e.g., IgE, skin testing, complement activation, serum tryptase, inhibitory antibody, immune complex), if performed (see Sections 9.4.3.3, 9.4.3.4, and 9.4.3.5 of this protocol for additional information), will also be summarized by treatment group. Graphical displays may be presented as appropriate.

11.7.3.5 Adverse Events

Medical Dictionary for Regulatory Activities (MedDRA™)-coded AE verbatim terms will be summarized by primary system organ class (SOC) and preferred term. Listings will include lower level terms (LLT).

The incidence of treatment emergent AEs will be tabulated by treatment group. Adverse events will also be summarized by severity and by relationship to treatment and presented by treatment group. Additional tabulations by treatment group will include AEs considered to be IARs (see Section 9.4.5.1 for further information on IARs), AEs leading to study discontinuations, dose increase due to disease progression, and SAEs. A detailed listing of patients who experience AEs and SAEs will be presented.

11.7.3.6 Concomitant Medications and Therapies

Concomitant medications and therapies are described in Section 9.4.6 and Section 9.4.7, respectively.

Concomitant medications will be summarized by frequency for all patients and for each dose group. Pre-treatment medications will be summarized separately.

11.8 Planned Interim Analysis

After all patients have been treated for 1 year, an interim analysis will be performed. Demographics, Baseline characteristics, and disposition will be summarized by treatment group. The primary endpoint (skin GL-3 clearance) will be summarized for both treatment groups. The relevant secondary and exploratory endpoints may also be summarized for each treatment group. Safety related parameters (possibly including but not limited to AEs, SAEs, vital signs, IgG titers, and study drug exposure data) may also be summarized for each treatment group.

12. SPECIAL REQUIREMENTS AND PROCEDURES

This protocol was designed and will be conducted, recorded, and reported in compliance with the principles of Good Clinical Practice (GCP) regulations. These requirements are stated in international regulations such as the International Conference on Harmonization (ICH) Guideline E6 Good Clinical Practice and local regulations such as the US Code of Federal Regulations (CFR) as well as the "Guidance for Good Clinical Practice," ICH of Technical Requirements for Registration of Pharmaceuticals for Human Use, Part C, Division 5 of Health Canada's Food and Drugs Act, and the EU Clinical Trial Directive (2001/20/EC) and EU GCP Directive 2005/28/EC.

12.1 Institutional and Ethical Review

This protocol and patient informed assent/consent form must be reviewed and approved by an IRB, an IEC, a REB, or any other appropriate regulatory authority in compliance with ICH and the local requirements of 21 CFR 50 and 56 in the US before the enrollment of patients. The letter or certificate of approval from the appropriate ethical and/or regulatory authority and the approved assent/consent form must be received by the Sponsor prior to delivery of clinical supplies.

12.2 Changes to the Conduct of the Study or Protocol

No change in the study procedures shall be effected without the mutual agreement of the Study Investigator and the Sponsor. All changes must be documented by signed protocol amendments. If significant change to the design of the study is made, the amendment must be submitted to, and approved by the appropriate ethical and/or regulatory authority, signed by the Study Investigator, and returned to the Sponsor.

12.3 Investigator's Responsibilities

12.3.1 Patient Informed Assent/Consent

Written informed assent/consent is required prior to enrollment in the study. It is the responsibility of the Study Investigator to obtain such assent/consent. Study Investigators must comply with local guidelines and regulations when developing the patient informed assent/consent.

The Study Investigator must furnish the Sponsor with a copy of the proposed assent/consent forms prior to submitting to the appropriate ethical and/or regulatory authority so that the Sponsor may ensure that all appropriate elements are incorporated into the document. Upon approval by the appropriate ethical and/or regulatory authority, the Study Investigator must furnish: (1) a copy of the approved informed assent/consent, and (2) the letter stating formal approval has been granted by the institution, prior to release of clinical supplies.

12.3.2 Case Report Forms

Data will be entered by the site onto the eCRFs. ***Note:*** eCRFs must not be used as source documents.

Copies of pertinent records in connection with the study will be made available to the Sponsor or designee on request, with due precaution towards protecting the privacy of the patient. Pertinent records include, but are not limited to, patient charts and laboratory data and will be available in a timely manner throughout the course of the study.

12.3.3 Record Retention

Essential documents should be retained until at least 2 years after study completion. These documents should be retained for a longer period however if required by the applicable regulatory requirements (for example, 25 years in Canada) or by an agreement with the Sponsor. It is the responsibility of the Sponsor to inform the Study Investigator/institution as to when these documents no longer need to be retained.

Essential documents are those documents, which individually and collectively, permit evaluation of the conduct of a study and the quality of the data produced. These documents serve to demonstrate the compliance of the Study Investigator, Sponsor and monitor with the standards of GCP and with all applicable international and local regulatory requirements.

Any or all of the documents must be available for, audit by the Sponsor’s auditor and inspection by the regulatory authority(ies).

12.3.4 Monitoring

A representative of the Sponsor will visit the Study Investigator periodically for the purpose of monitoring the progress of this study in accordance with GCP regulations. It is the responsibility of the Study Investigator to be present or available for consultation during such scheduled monitoring visits. During these routine visits, all data pertaining to a patient's participation in this clinical investigation must be made available to the monitor.

An audit may be performed at any time during or after completion of the clinical study by the Sponsor personnel or their designee. All study-related documentation must be made available to the designated auditor.

In addition, a representative of a regulatory agency may choose to inspect a study site at any time prior to, during, or after completion of the clinical study. A representative of the Sponsor will be available to assist in the preparation for such an inspection. All pertinent study data should be made available to the regulatory authority for verification, audit, or inspection purposes.

12.3.5 Materials Control

12.3.5.1 Receipt of Clinical Supplies

Upon receipt of any study medication, the study site personnel will open the shipment, verify the contents as stated on the enclosed shipping form, sign and date the form, retain a copy, and fax the form to the Sponsor or designee. Records will be kept of all drug received, administered and returned on the forms provided. The Study Investigator must insure that the study treatment is maintained under required conditions (2 to 8°C) in a secure area with restricted access. See the Investigational Product Handling Manual for further instructions.

12.3.5.2 Disposition of Unused Clinical Supplies

Return/Destruction Authorization Forms must be completed by the Study Investigator or designee each time material is returned or destroyed, but only when authorized by the Sponsor. If any unused material is remaining at the site pharmacy upon completion of the study, the pharmacy will be instructed to destroy or return the material to the Sponsor. See the Investigational Product Handling Manual for further instructions.

12.3.5.3 Tracking and Disposition of Clinical Supplies

The responsible pharmacist or designee must perform accountability of the clinical supplies using the forms provided each time an infusion is prepared. Used vials may be stored at room temperature and must be retained at the pharmacy until further notice by the Sponsor. Unused vials must be retained at the pharmacy, under adequate storage conditions (2 to 8°C), until further notice by the Sponsor. See the Investigational Product Handling Manual for further instructions.

12.3.5.4 Product Handling and Complaints Reporting

If there are any issues during the course of the study related to the quality of the Investigational Product, the pharmacist or pharmacy designee is to contact Genzyme CPRS. Where possible, store the product as instructed in a limited access area until otherwise instructed by the Sponsor. The Sponsor's CPRS facsimile number is provided below and is available for product handling and complaint reporting on a 24-hour basis and is reviewed during normal business hours.

Genzyme CPRS Product Handling and Complaint Reporting	
**North America / International**	**Europe**
Phone: +1 (800) 326-7002<br>Fax: +1 (508) 424-4484 or +1 (877) 237-1292 (toll-free)<br>Email: CPRSProductComplaints@genzyme.com	Phone: +31(0) 35 699 1200<br>Fax: +31(0) 35 699 1403<br>Email: CPRSProductComplaints@genzyme.com

12.3.6 Warnings, Precautions, Contraindications

For specific information concerning warnings, precautions, and contraindications, the Study Investigator is asked to refer to the appropriate section of the IB or Product Labeling. Because of the possibility of AEs, appropriate emergency response measures should be available.

12.3.7 Clinical Study Report

If deemed appropriate by the Sponsor, a Study Investigator(s) shall be designated to sign the completed clinical study report at the end of this study.

The signatory Study Investigator(s) shall be identified by the Sponsor upon the completion of the study, based upon:

- Patient enrollment (i.e., the individual who enrolls the largest number of patients).
- The individual's participating in the design of the study.

12.3.8 Disclosure of Data

All information obtained during the conduct of this study will be regarded as confidential, and written permission from the Sponsor is required prior to disclosing any information relative to this study. Manuscripts prepared for publication will be in accordance with the policy established and presented to the Study Investigator by the Sponsor. Submission to the Sponsor for review and comment prior to submission to the publisher will be required. This requirement should not be construed as a means of restricting publication, but is intended solely to assure concurrence regarding data, evaluations, and conclusions and to provide an opportunity to share with the Study Investigator any new and/or unpublished information of which he/she may be unaware.

13. REFERENCES

[1] Pierides AM, Holti G, Crombie AL, Roberts DF, Gardiner SE, Colling A, Anderson J. Study on a family with Anderson-Fabry's disease and associated familial spastic paraplegia. *J Med Gen.* 1976;13:455-461.

[2] Lidove O, Joly D, Rossignol P, Robino C, N'Guessau K, Grünfeld JP. Nephroquiz for the Beginner A man with proteinuria, familial history of kidney disease, painful extremities and cutaneous lesions. *Nephrol Dial Transplant*. 2000;15:433-435.

[3] Verovnik F, Benko D, Vujkovac B, Linthorst GE. Remarkable variability in renal disease in a large Slovenian family with Fabry Disease. *Eur J Hum Genet*. 2004;12:678-681.

[4] Ferrans VJ, Hibbs RG, Burda CD. The heart in Fabry's disease. A histochemical and electron microscopic study. *Am J Cardiol*. 1969 July;24:95-110.

[5] Gubler MC, Lenoir G, Grünfeld JP, Ulmann A, Droz D, Habib R. Early renal changes in hemizygous and heterozygous patients with Fabry's disease. *Kidney Int*. 1978;13:223-235.

[6] Meuwissen SGM, Dingemans KP, Strijland A, Tager JM, Ooms BCM. Ultrastructural and biochemical liver analyses in Fabry's disease. *Hepatology*. 1982;2(2):263-268.

[7] Aivazian AA, Trofimov IB, Seredniakova NI. Ultrastructural changes in the skin of patients with Fabry's angiokeratoma. *Arkh Patol*. 1985;47(7):60-63.

[8] Drachenberg CB, Schweitzer EJ, Bartlett ST, Behrens MT, Papadimitriou JC. Polyarteritis nodosa-like necrotizing vasculitis in Fabry Disease. *J Inher Metab Dis*. 1993;16:901-902.

[9] Desnick RJ, Ioannou YA, Eng CM. α-Galactosidase A deficiency: Fabry Disease. In: Scriver CR, Beaudet AL, Sly WS, Valle D, eds. The Metabolic and Molecular Bases of Inherited Disease. 8th edn. Vol 1. New York: McGraw-Hill; 2001:3733–3734.

[10] Breunig F, Wanner C. Enzyme replacement therapy for Fabry Disease: proving the clinical benefit. *Nephrol Dial Transplant*. 2003;18:7-9.

[11] Tsutsumi A, Uchida Y, Kanai T, Tsutsumi O, Satoh K, Sakamoto S. *Acta Ophthalmol (Copenh)*. 1984;62:923-931.

[12] Vedder AC, Strijland A, vd Bergh Weerman MA, Florquin S, Aerts JMFG, Hollak CEM. Manifestations of Fabry Disease in placental tissue. *J Inherit Metab Dis*. 2006;29:106-111.

[13] Ries M, Ramaswami U, Parini R, Lindblad B, Whybra C, Willers I, Gal A, Beck M.. The early clinical phenotype of Fabry Disease: a study on 35 European children and adolescents. *Eur J Pediatr*. 2003 Nov;162(11):767-772.

[14] Ries M, Clarke JTR, Whybra C, Timmons M, Robinson C, Schlaggar BL, et al. Enzyme-replacement therapy with agalsidase alfa in children with Fabry Disease. *Peds*. 2006;118(3):924-932.

[15] Taddeucci G, Tarantino E, Bonuccelli A, Barsotti G, Polacco P. Ischemic stroke in childhood: transient cerebral arteriopathy. *Ital J Pediatr*. 2004;30:11-13.

[16] Ries M, Gupta S, Moore DF, Sachdev V, Quirk JM, Murry GJ, et al. Pediatric Fabry Disease. *Pediatrs*. 2005 Mar;115(3):e344-355.

[17] Linhart A, Palecek T, Bultas J, Ferguson JJ, Hrudova J, Karetova D, et al. New insights in cardiac structural changes in patients with Fabry's Disease. *Am Heart J*. 2000;139(6):1101–1108.

[18] Moore DF, Altarescu G, Ling GSF, Jefferies N, Frei KP, Weibel T, et al. Elevated cerebral blood flow velocities in Fabry Disease with reversal after enzyme replacement. *Stroke*. 2002;33:525-531.

[19] Thadhani R, Wolf M, West ML, Tonelli M, Ruthazer R, Pastores GM, Obrador GT. Patients with Fabry Disease on dialysis in the United States. *Kidney Int*. 2002;61:249–255.

[20] Desnick RJ, Astrin KH, Bishop DF. Fabry Disease: molecular genetics of the inherited nephropathy. *Adv Nephrol*. 1989;18:113-127.

[21] Banikazemi M, Bultas J, Waldek S, Wilcox WR, Whitley CB, McDonald M, et al. Agalsidase-beta therapy for advanced Fabry Disease. *Ann Intern Med*. 2007;146:77-86.

[22] Wilcox WR, Banikazemi M, Guffon N, Waldek S, Lee P, Linthorst GE, et al. Long-term safety and efficacy of enzyme replacement therapy for Fabry Disease. *Am J Hum Genet*. 2004;75:65-74.

[23] Germain DP, Waldek S, Banikazemi M, Bushinsky DA, Charrow J, Desnick RJ, et al. Sustained, long-term renal stabilization after 54 months of agalsidase β therapy in patients with Fabry Disease. *J Am Soc Nephrol*. 2007 doi:10.1681/ASN.2006080816;1-11.

[24] Eng CM, Guffon N, Wilcox WR, Germain DP, Lee P, Waldek S, et al. Safety and efficacy of recombinant human α-galactosidase A replacement therapy in Fabry's Disease. *N Engl J Med*. 2001;345:9-16.

[25] Linthorst GE, Hollak CEM, Donker-Koopman WE, Strijland A, Aerts JMFG. Enzyme therapy for Fabry Disease: neutralizing antibodies toward agalsidase alpha and beta. *Kidney Int*. 2004;66:1589-1595.

[26] Vedder AC, Linthorst GE, Houge G, Groener JEM, Ormel EE, Bouma BJ, et al. Treatment of Fabry Disease: outcome of a comparative trial with agalsidase alfa or beta at a dose of 0.2 mg/kg. PLoS ONE 2007;2(7);e598. doi:0.1371/journal.pone.0000598

[27] Ohashi T, Sakuma M, Kitagawa T, Suzuki K, Ishige N, Eto Y. Influence of antibody formation on reduction of globotriaosylceramide (GL-3) in urine from Fabry patients during agalsidase beta therapy. *Mol Genet Metab* (2007), doi:10.1016/j.ymgme.2007.06.013.

[28] Kampmann C, Wiethoff CM, Wenzel A, Stolz G, Betancor M, Wippermann C-F, et al. Normal values of M mode echocardiographic measurements of more than 2000 healthy infants and children in central Europe. *Heart*. 2000;83;667-672.

[29] Gaspari F, Perico N, Ruggenenti P, Mosconi L, Amuchastegui CS, Guerini E, et al. Plasma clearance of nonradioactive Iohexol as a measure of glomerular filtration rate. *J Am Soc Nephrol*. 1995;6(2):257-263.

[30] Wiseman MJ, Hunt R, Goodwin A, Gross J, Keen H, Viberti GC. Dietary composition and renal function in healthy subjects. *Nephron*. 1987;46:37-42.

[31] Maroni BJ, Steinman TI, Mitch WE. A method for estimating nitrogen intake of patients with chronic renal failure. *Kidney Int*. 1985;27:58-65.

[32] Schwartz GJ, Haycock GB, Edelmann Jr CM, Spitzer A. Simple estimate of glomerular filtration rate in children derived from body length and plasma creatinine. *Pediatrics*. 1976 Aug;58(2):259-263.

[33] Wetzels JFM, Kiemeney LALM, Swinkels DW, Willems HL, den Heijer M. Age- and gender-specific reference values of estimated GFR in Caucasians: The Nijmegen Biomedical Study. *Kidney Int*. 2007;72(5):632–637.

[34] Wetzels JFM, Willems HL, den Heijer M. Age- and gender-specific reference values of estimated GFR in Caucasians: The Nijmegen Biomedical Study. *Kidney Int*. 2008;73(5):657–658.

[35] EMA note of guidance (ICH Topic E11) for clinical investigation of medicinal products in the paediatric population. April 2006. (www.ifpma.org/pdfifpma/E11step4.pdf).

[36] Tanner JM. Growth at Adolescence. 2nd ed. Oxford: Blackwell Scientific Publications; 1962.

14. APPENDICES

14.1 Notification Procedures and Guidelines for the Management of Infusion-Associated Reactions

Patient experiences infusion-associated reaction (IAR) symptoms

⇓

Study site and/or home care nurse manages patient according to the general standards of care. Study site determines the severity of the infusion-associated reactions (IARs). For moderate-severe IARs, or recurrent IARs suspected to be IgE-mediated, the site should obtain serum and plasma samples for serum tryptase and plasma complement testing within 1 to 2 hours after the onset of the event and immediately send these with the pre-infusion serum sample for IgG and possibly IgE testing. For home infusions, the home infusion nurse will draw the specimens for complement activation and tryptase whenever possible and where permitted by local regulations. If a pre-infusion serum sample was not obtained, the patient will be asked to return to the site after the reaction, either at least 3 days post infusion or prior to the next infusion, for serum sample collection. Global Patient Safety and Risk Management may be contacted (see Section 9.4.5.3 and SOM for contact information) at the discretion of the study site to discuss the events.

⇓ ⇓

Americas	Europe
Study Investigator from study site notifies Global Patient Safety and Risk Management (GPS-RM) Department US<br>⇓<br>Site faxes (**Fax** +1 (617) 761-8506) IAR or SAE form (downloaded from eCRF) to GPS-RM US within 24 hours of the event<br>⇓<br>GPS-RM notifies the central laboratory that sample for testing is en route and whether testing should be performed<br>⇓<br>GPS-RM US instructs specialty laboratory to ship skin test supplies to site if appropriate<br>⇓<br>the central laboratory provides results of testing to GPS-RM<br>⇓<br>GPS-RM US notifies PI of results prior to subsequent infusion	Study Investigator from study site notifies Global Patient Safety and Risk Management (GPS-RM) Department US<br>⇓<br>Site faxes (**Fax** +1 (617) 761-8506) IAR or SAE form (downloaded from eCRF) to GPS-RM US within 24 hours of the event<br>⇓<br>GPS-RM EU notifies the central laboratory that sample for testing is en route and whether testing should be performed<br>⇓<br>GPS-RM Europe instructs specialty laboratory to ship skin test supplies to site if appropriate<br>⇓<br>the central laboratory provides results of testing to GPS-RM<br>⇓<br>GPS-RM Europe notifies PI of results prior to subsequent infusion

⇓ ⇓

If patient is **IgE^+ (positive)** or has elevated tryptase the decision to continue or discontinue therapy will be evaluated by the DMC and an independent allergist in collaboration with the Study Investigator and the Sponsor.

If patient is **IgE^- (negative)**, patient may be skin tested (see SOM for details) prior to receiving the next infusion:

If results of **skin test are positive**, the decision to continue or discontinue therapy will be evaluated by the DMC and an independent allergist in collaboration with the Study Investigator and the Sponsor.

14.2 Suggested Guidelines for Management of Infusion-Associated Reactions During the Event

Patients experiencing symptoms suggestive of an IAR should be managed according to the general standards of care consistent with the treatment of the reaction. Following are guidelines for the management of these symptoms, grouped by the severity of the reaction.

14.2.1 Suggested Management of Mild Reactions

**Mild Reactions:** Symptom(s) barely noticeable to the patient or does not make the patient uncomfortable. The AE does not influence performance or functioning. Prescription drugs are not ordinarily needed for relief of symptom(s).

1. Slow infusion rate to ½ the original rate (**be sure to record rate change and time of change**).
2. Administer diphenhydramine HCl (ages 5 to 12 years: 1.25 mg/kg, IV; patients 12 years or older: 25 to 50 mg, IV) or pharmacological equivalent such as chlorpheniramine (ages 5 to 12 years: 2 mg; 12 years or older: 4 mg). Paracetomol/acetaminophen (ages 5 to 12 years: 15 mg/kg; 12 years or older: 650 to 1000 mg) may be administered based on the nature of the symptoms experienced.
3. Resume previous infusion rate if symptoms subside by first increasing infusion rate by 50% for 15 to 30 minutes and if no symptoms reoccur, increase to the previous infusion rate.
4. Stop infusion if symptoms increase in severity (**be sure to record rate change and time of change**).
5. Refer to Section 14.2.5 for collection of required antibody testing/samples.

14.2.2 Suggested Management of Moderate Reactions

**Moderate Reactions:** Symptom(s) of a sufficient severity to make the patient uncomfortable. Performance of daily activities is influenced. Treatment of symptom(s) may be needed.

1. To assess symptoms/severity, consider stopping infusion. Alternatively, slow infusion rate to 1/2 the original infusion rate (**be sure to record rate change and time of change**).
2. Administer diphenhydramine HCl, (ages 5 to 12 year: 1.25 mg/kg, IV; 12 years or older: 25 to 50 mg, IV) or pharmacological equivalent such as chlorpheniramine (ages 5 to 12 years: 2 mg; 12 years or older: 4 mg).
3. For respiratory symptoms, administer inhaled β agonist (e.g., albuterol).
4. If respiratory symptoms severity warrants, administer epinephrine, 1:1000, 0.01 cc/kg (maximum 0.3 cc), subcutaneously (SC) in upper extremity or thigh.
5. Consider addition of prednisolone 1-2 mg/kg IV or hydrocortisone 4-8 mg/kg IV, or a pharmacological equivalent.
6. Consider the addition of paracetamol/acetaminophen (ages 5 to 12 years: 15 mg/kg; 12 years or older: 650 to 1000 mg) or ibuprofen (ages 5 to 12 years: 10 mg/kg; 12 years or older: 400 to 800 mg).

7. Stop infusion if symptoms increase in severity or persist (**be sure to record rate change and time of change**).
8. If symptoms subside, resume previous infusion rate by first increasing infusion rate by 50% for 15 to 30 minutes. If no symptoms reoccur, increase to previous infusion rate.
9. Refer to Section 14.2.5 for collection of required antibody testing/samples.

14.2.3 Suggested Management of Severe Reactions

**Severe Reactions:** Symptom(s) of a sufficient severity to cause the patient severe discomfort. Severity may cause cessation of treatment with the study drug. Treatment for symptom(s) may be given.

1. Immediately terminate infusion.
2. Administer epinephrine, 1:1000, 0.01 cc/kg (maximum 0.3 cc), SC in upper extremity or thigh.
3. Administer diphenhydramine HCl (ages 5 to 12 years: 1.25 mg/kg, IV; 12 years or older: 25 to 50 mg) or a pharmacological equivalent such as chlorpheniramine (ages 5 to 12 years: 2 mg; 12 years or older: 4 mg).
4. Administer prednisolone 1 to 2 mg/kg IV or hydrocortisone 4 to 8 mg/kg IV or a pharmacological equivalent.
5. For respiratory symptoms, administer β agonist (e.g., albuterol) via metered dose inhaler or nebulizer.
6. For significant dyspnea, cyanosis, or wheezing, administer moderate to high-flow oxygen by mask or nasal catheter.
7. Manage fluid volume by intravenous fluids.
8. Institute advanced cardiopulmonary resuscitation (ACLS) measures as appropriate.
9. Refer to Section 14.2.5 for collection of required antibody testing/samples.

14.2.4 Management of Recurrent Infusion-Associated Reactions

For patients who experience recurrent IARs, infusion rate adjustment and pre-treatment measures should be taken prior to subsequent infusions. Pre-treatment regimens should be determined based on the nature and severity of the symptoms. The Sponsor will review all reported IARs and, in conjunction with an outside immunology consultant, discuss IAR management strategies with the site as needed in order to maximize benefit and minimize risk to the patient. The following pre-treatment measures should be considered:

Severity of Event	Mild to Moderate Recurrent Event	Severe Recurrent Event
Infusion Rate Adjustment	• If symptoms occur shortly after increasing the infusion rate, consider prolonging the infusion time of the previous step during next infusion	• Consider halving the infusion rate step that led to the event
Pre-Treatment Regimen for Next Infusion	**Consider adding the following agents based on the nature of symptoms experienced**:	
	1 hour prior to infusion:<br><br>**Antipyretics:**<br>▪ Paracetamol/Acetaminophen (orally [PO]):<br>**5 to 12 yr old**: 15 mg/kg (not to exceed [NTE] 1000 mg)<br>**12 years or older**: 650 to 1000 mg<br>OR<br>▪ Ibuprofen (PO):<br>**5 to 12 years old**: 10 mg/kg (NTE 800 mg)<br>**12 years or older**: 400 to 800 mg<br><br>**H1-histamine blocker:**<br>▪ Diphenhydramine HCl (IV/PO):<br>**5 to 12 years old:** 1.25 mg/kg (NTE 50 mg)<br>**12 years or older:** 25 to 50 mg<br>OR<br>▪ Chlorpheniramine (IV/PO):<br>**5 to 12 years old**: 2 mg<br>**12 years or older**: 4 mg<br>OR<br>▪ Hydroxyzine (PO)<br>**5 to 12 years old**: 12.5 to 25 mg<br>**12 years or older**: 25 to 50 mg	7 hours prior to infusion:<br><br>**Steroids:**<br>▪ Prednisone/Prednisolone (PO):<br>**5 to 12 years old:** 1 to 2 mg/kg (NTE 50 mg)<br>**12 years or older:** 50 mg<br>1hr prior to infusion:<br><br>**Antipyretics:**<br>As suggested for mild to moderate recurrent events.<br><br>**H1-histamine blockers:**<br>As suggested for mild to moderate recurrent events.<br><br>**Steroids:**<br>▪ Repeat dose of prednisone/prednisolone<br>OR<br>▪ Methylprednisolone (PO/IV):<br>**5 to 12 years old**: 0.8 mg/kg to 1.6 mg/kg (NTE 40 mg)<br>**12 years or older**: 40 mg
IV = intravenous; PO = by mouth; NTE = not to exceed		

Refer to Section 14.2.5 for collection of required antibody testing/samples.

14.2.5 Routine Antibody Sampling and Sample Collection and Skin Testing in case of Infusion-Associated Reactions

IgG

Serum samples will be collected for assessment of IgG antibody formation to Fabrazyme starting Day 1 and every 4 weeks for the first 6 months (through Week 28/Month 6/Year 0.5); then every 3 months for 6 months (e.g., twice; through Week 52/Year 1) then every 6 months thereafter.

Additional testing for IgG antibodies may be performed for patients who experience a moderate to severe IAR or a recurrent IAR of any intensity. If the IAR occurs at an infusion when a pre-infusion IgG sample was not collected, the patient will be asked to return to the site after the reaction, at least 3 days post infusion or prior to the next infusion, for serum sample collection for IgG antibody testing.

All serum samples for IgG testing will be shipped to a central laboratory and can be batched. Samples should be shipped at least every 2 to 3 months. However, in case an IAR occurs that requires IgG or IgE analysis, samples should be shipped immediately.

Refer to the SOM for detailed instructions on collection, processing, and shipment of samples.

IgE

Serum samples will be collected for assessment of IgE antibody formation to Fabrazyme in case of moderate to severe hypersensitivity-type reactions. The sample could be the pre-infusion sample as taken for IgG testing, if available. Should a pre-infusion sample not be available, the patient will be asked to return to the study site, at least 3 days post infusion or prior to the next infusion, for serum sample collection for IgE antibody testing. Samples should be shipped immediately.

Refer to the SOM for detailed instructions on collection, processing, and shipment of samples.

Global Patient Safety and Risk Management should be contacted in case of moderate-severe hypersensitivity-type reactions to determine the need for IgE testing.

Complement Activation and Serum Tryptase Sample

Plasma samples for complement activation and serum samples for tryptase levels will be collected in case of moderate to severe hypersensitivity-type reactions. The samples should be drawn within 1 to 2 hours after the onset of the symptoms. Therefore, the study site should be prepared to draw specimens for complement activation and tryptase as described in the protocol, should a moderate or severe IAR occur during the infusion. For home infusions, the home infusion nurse may need to draw the specimens for complement activation and tryptase, as permitted by local regulations. Samples should be shipped immediately.

Refer to the SOM for detailed instructions on collection, processing, and shipment of samples.

GPS-RM Department should be contacted in case of moderate-severe hypersensitivity-type reactions to determine the need for testing.

Skin Testing

In case of moderate to severe IARs in patients that were tested negative for IgE or with inconclusive results, a skin test should be performed according to the procedures detailed in the SOM. The Sponsor will supply the study drug for skin testing.

Summary of Hypersensitivity Laboratory Tests to be Obtained (Based on IAR Experienced)

	**IgG[a] (serum)**	**IgE[b] (serum)**	**Tryptase[c] (serum)**	**Complement[c] (plasma)**
**No Event/Routine**	X			
**Mild Event**				
**Recurrent Mild Events**	X			
**Moderate Event**	X	**X[de]**	**X[d]** Within 1 to 2 hours of the onset of the reaction	**X[d]** Within 1 to 2 hours of the onset of the reaction
**Recurrent Moderate Events**	X	**X[de]**	**X[d]** Within 1 to 2 hours of the onset of the reaction	**X[d]** Within 1 to 2 hours of the onset of the reaction
**Severe Event**	X	**X[e]**	**X** Within 1 to 2 hours of the onset of the reaction	**X** Within 1 to 2 hours of the onset of the reaction
**Recurrent Severe Events**	X	**X[e]**	**X[d]** Within 1 to 2 hours of the onset of the reaction	**X** Within 1 to 2 hours of the onset of the reaction

a If no pre-infusion serum sample for antibody testing is available and IgG should be tested, then the patient will be asked to return to the site at least 3 days post infusion or prior to the next infusion for serum sample collection for antibody testing.

b For IgE testing pre-infusion serum samples for IgG could be used if taken at that specific visit. If no pre-infusion serum sample for antibody testing is available and IgE should be tested, then the patient will be asked to return to the site at least 3 days post infusion or prior to the next infusion for serum sample collection for antibody testing. Patients will need to have serum IgE results **prior** to their next infusion.

c For home infusions, the home infusion nurse will draw and store a serum sample (for serum tryptase) and plasma sample (for complement activation) at the specified timepoints, whenever possible and where permitted by local regulations.

d Only performed in specific cases at the Study Investigator's discretion or in discussion with Global Patient Safety and Risk Management.

e Consider whether skin testing is needed.

14.3 Tanner Stages

Classification of genitalia maturity stages in boys

Stage	Pubic hair	Penis	Testes
**I**	Preadolescent - No pubic hair present; a fine vellus hair covers genital area	Preadolescent- Same as in childhood	Preadolescent -Testes and scrotum the same as in childhood - Testes less than 3 mL in volume
**II**	Sparse distribution of long, slightly pigmented hair appears at base of penis	Slight enlargement	The testes enlarge - Scrotum enlarges, developing a reddish hue and altering in skin texture
**III**	Hair pigmentation increases; begins to curl and spread laterally in a scanty distribution	Longer	Testes and scrotum continue to grow
**IV**	Resembles adult type in being coarse and curly but less in quantity; adult type of distribution is attained	Grows in width, and the glans penis develops.	Testes and scrotum continue to grow; the scrotal skin darkens
**V**	Mature - adult distribution; spread to medial surface of thighs	Adult size and shape	Mature - Testes and scrotum are adult size

Adapted from Tanner IM: Growth at Adolescence. Oxford, Blackwell, 1962.
